# Assessing the impact of caregiving on informal caregivers of adults with a mental disorder in OECD countries: A systematic literature review of concepts and their respective questionnaires

**DOI:** 10.1371/journal.pone.0270278

**Published:** 2022-07-08

**Authors:** Leonarda G. M. Bremmers, Isabelle N. Fabbricotti, Eleonora S. Gräler, Carin A. Uyl-de Groot, Leona Hakkaart-van Roijen

**Affiliations:** Erasmus School of Health Policy & Management, Erasmus University Rotterdam, Rotterdam, The Netherlands; Cardiff University, UNITED KINGDOM

## Abstract

We conducted a systematic literature review to identify and review the concepts and questionnaires used to assess the impact of caregiving on caregivers for adults with a mental disorder. With our study, we aimed to provide an overview and categorize the conceptualization and operationalization of the impact of caregiving, with special attention for the complexity and multi-conceptualization of concepts. Embase, Medline, PsycInfo, Web of Science Core Collection, Cochrane Central Register of Trials, Cinahl Plus, Econlit and Google Scholar were systematically searched for articles from 1 January 2004 to 31 December 2019. Eligible articles were peer-reviewed studies that assessed the impact of caregiving for informal caregivers of adults with a reported mental disorder by means of a questionnaire. The complete study protocol can be found on PROSPERO (CRD42020157300). A total of 144 questionnaires were identified that assessed the impact of caregiving. Based on similarities in meaning, concepts were classified into 15 concept clusters. The most frequently assessed concept clusters were mental health, caregiving burden, other caregiving consequences, family impact, and overall health-related outcomes. The use of concept clusters differed per diagnosis group, with diagnoses, such as schizophrenia, using a wide range of caregiving impact concepts and other diagnoses, such as personality disorders, only using a limited range of concepts. This is the first study that identified and reviewed the concepts and questionnaires that are used to assess the impact of caregiving. Caregiving is researched from a broad array of perspectives, with the identification of a variety of concepts and dimensions and use of non-specific questionnaires. Despite increasing interest in this field of research, a high degree of variability remains abundant with limited consensus. This can partially be accredited to differences in the naming of concepts. Ultimately, this review can serve as a reference to researchers who wish to assess the impact of caregiving and require further insight into concepts and their respective questionnaires.

## Introduction

The mid-twentieth century saw a rise in the international consensus on the need for decentralized psychiatric care and new policy strategies for mental health patients. This consensus resulted in a radical deinstitutionalization movement across the USA, England, Continental Europe, and Scandinavia, with other countries later following suit [1]. The movement was characterized by a shift of care from the institutions to community-based services, with a strong focus on the reintegration and rehabilitation of patients [1, 2]. However, fragmented community-based services often fail to address patients’ complex health needs [2], as suggested by the high prevalence of incarceration, homelessness, loneliness, victimization, and poor physical health outcomes of patients [2–6]. Consequently, patients are increasingly reliant on the care and support provided by their loved ones, hereinafter referred to as *informal care* [7, 8]. The health care sector relies heavily on informal care, as it complements and substitutes services provided by formal care providers [9–12].

The provision of informal care is often characterized as a significant source of distress for the loved ones of patients and can have a detrimental impact on their daily lives and wellbeing [13]. Hence, the impact of caregiving should be considered in healthcare practice and policy [14, 15]. Perspectives on the impact of caregiving and mental illness have evolved with the introduction of deinstitutionalization [16, 17]. Before the turn of the century, caregiver research centered on two concepts, the negative impact of the patient on the caregiver (i.e., caregiving burden) and the negative impact of the caregiver on the patient (i.e., expressed emotion). This research was largely concentrated on caring for patients with schizophrenia; however, burden was also assessed for caregivers of patients with mood disorders. Over the decades, additional concepts have been developed to assess the rewarding aspects of caregiving, such as caregiving reward [18]. However, Harvey et al. found that caregiver outcomes reported in peer-reviewed articles are still restricted in scope and primarily focus on wellbeing, the caregiving experience, and need for professional support [19].

Despite the impact of caregiving being studied since the start of deinstitutionalization [18], the operationalization and conceptualization of these concepts has received limited academic attention [20]. There are a limited number of conceptual frameworks grounded in psychological and social theories for this caregiving population, with the existing frameworks primarily focused on familial responses to mental disorders [21]. Consequently, researchers report an inconsistent use of theoretical definitions and operationalization across the same concepts [21, 22]. Ergo, the conceptualization and operationalization of the impact of caregiving may vary greatly between studies. To the best of our knowledge, no systematic literature review has yet investigated the conceptualization and operationalization of the impact of caregiving in this caregiver population. A literature review conducted by Schene, Tessler, and Gamache compiled caregiving questionnaires and their respective domains; however, this was limited to one concept, namely caregiving burden [23].

A complete overview of the conceptualization and operationalization of the impact of caregiving could improve the understanding of these concepts [24] and aid in determining how they are used in scientific research. By systematically identifying the similarities and discrepancies of concepts and their respective dimensions across questionnaires, an in-depth insight can be gained into the perspectives that are used in caregiver research. These insights may help researchers to select the appropriate concepts and questionnaires and improve comparability of results across studies. Therefore, we conducted a systematic literature review to identify and review the concepts and questionnaires that are used to assess the impact of caregiving on caregivers for adult patients with mental disorders in OECD countries. With our study, we aimed to provide an overview and categorize the conceptualization and operationalization of the impact of caregiving, with special attention for the complexity and multi-conceptualization of concepts.

## Methods

This systematic literature review was reported in accordance with the Preferred Reporting Items for Systematic Reviews and Meta-Analyses (PRISMA) guidelines. Refer to S1 Table for the completed PRISMA checklist [25]. The complete study protocol is registered on PROSPERO (CRD42020157300).

### Search strategy and data sources

The search strategy was constructed a priori with an information specialist using terms related to “informal caregivers,” “mental disorders,” and “questionnaires” [26]. On December 6, 2019, Embase, Medline, PsycInfo, Web of Science Core Collection, Cochrane Central Register of Trials, Cinahl Plus, Econlit, and Google Scholar were searched. The search was restricted to include articles published from January 1, 2004, onwards. For the complete search strategy refer to S1 File.

### Selection criteria

We included quantitative and mixed-method studies published in scientific journals, which reported original data and assessed the impact of caregiving by means of a questionnaire. The informal caregivers had to provide care and support to adults with a reported mental disorder. Relevant mental disorders were identified with the Fifth Edition of the Diagnostic and Statistical Manual of Mental Disorders [27]. Neurocognitive disorders and delirium were not considered, because the nature of these disorders and conditions is not comparable to other mental disorders [28] and thus has a significant impact on the reported caregiving experience [29, 30]. Additionally, care recipients with a physical comorbidity were excluded because they have different care needs and their caregivers are at a higher risk for adverse outcomes and events [31–34]. Care recipients and caregivers had to be at least 18 years of age. Studies needed to be conducted in countries within the Organization for Economic Co-operation and Development (OECD) region [35] to avoid cultural specificity that could be caused by differing cultural norms and perceptions [19]. Lastly, the review was restricted to empirical and peer-reviewed studies that were published in English.

### Selection of studies

Prior to the formal screening of hits, the selection criteria were piloted and adjusted amongst the research team (LB, LH, IF) using a randomly selected sample of hits (n = 50). A four-stage screening process was implemented using the selection criteria. First, all search hits were imported into Endnote X6, and duplicates were removed using a reproducible de-duplication method [36]. Second, title and abstract screening were conducted by two independent reviewers (LB, EG). Any disagreements concerning title and abstract eligibility were discussed with the other members of the research team (IF, LH). Third, the full-text articles were retrieved if the review criteria were met or if there was insufficient information in the abstract to assess eligibility. Fourth, full texts were independently screened by two reviewers (LB, IF) and those that met the inclusion criteria were included [25]. Any disagreements concerning article eligibility were discussed with a third reviewer from the research team (LH).

### Data extraction

Data were extracted by the primary researcher (LB) using a data extraction matrix. Relevant data included: country, study design, disorder of care recipient, questionnaire name, questionnaire author, concept studied, dimensions, operationalization of each dimension, and the original target population of the questionnaire. Given that some of the questionnaire data were not reported in the articles, it was sometimes necessary to refer to the questionnaires’ reported source article(s).

### Data analysis

All concepts were clustered according to the common phenomenon that they assessed. These clusters formed concept clusters which were then titled using the higher-order concept that they assessed. The concept clusters were generated by LB and then reviewed by the other co-authors (IF, CU and LH).

Meta summaries [37] were generated for each concept cluster and reported the dimensions of each questionnaire, including their operationalization. If the operationalization of the dimensions could not be found, then this was reported in the meta-summary as “not reported” (NR). For each meta summary, dimensions were grouped by theme. An overview and explanation of all relevant terms can be found in Table 1.

**Table 1:** Overview of relevant terms and their respective explanations.

Term	Explanation
**Concept clusters**	A collection of constructs based on the same abstract ideas and common phenomenon (e.g., all mental health conditions were classified under the concept cluster “mental health”)
**Concepts**	Constructs that assess the impact of caregiving
**Dimensions**	The internal attributes of a concept
**Operationalization of dimensions**	The definition of dimensions into measurable factors (i.e., questions)
**Themes**	Overarching ideas across dimensions

To investigate trends, the extracted data were grouped by concept clusters and graphed against the number of times it was assessed from 2004–2019. Additionally, the assessment of concept clusters was determined per diagnosis group.

## Results

### Literature review and study characteristics

**Fig 1:**
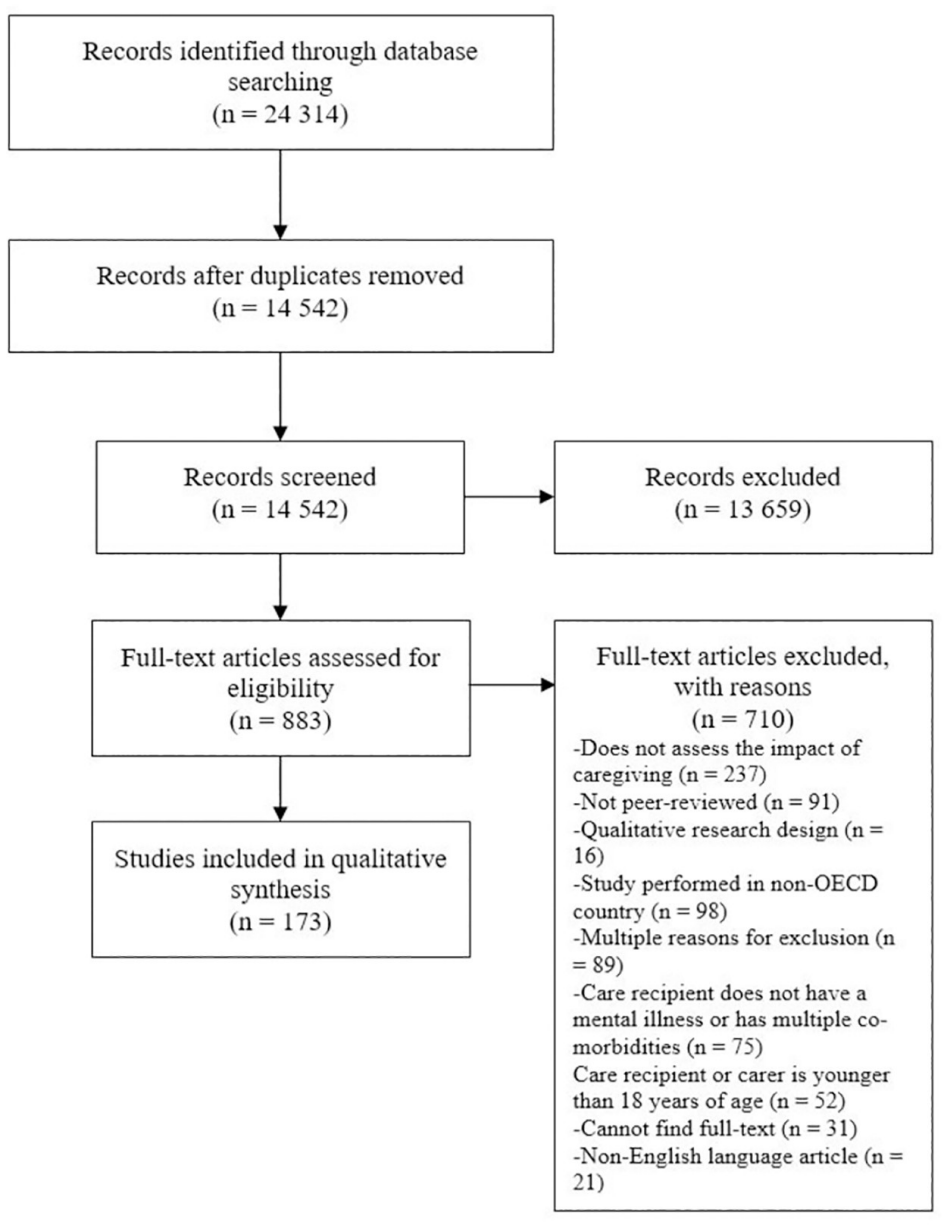
PRISMA flow chart.

The systematic search yielded a total of 24,314 reference with 9,772 duplicates. Title and abstract screening resulted in the exclusion of 13,659 papers. A total of 883 full-text articles were reviewed. The main reasons for full-text exclusion were, as follows: did not assess the impact of caregiving (n = 236), performed in non-OECD country (n = 98) or was not a peer-reviewed article (n = 91). A total of 173 papers fulfilled the eligibility criteria and were included (Fig 1).

All articles reported observational study designs, including cross-sectional (n = 131, 75%), case-control (n = 21, 12%), prospective cohort study designs (n = 19, 11%), and case-control and cross-sectional (n = 3, 2%). These studies were conducted in OECD countries across Asia, Australia, Europe, North America, and South America, with a majority of the studies being conducted in the United States of America (n = 33, 19%), the United Kingdom (n = 30, 17%), and Spain (n = 26, 15%). A variety of mental disorders were studied; however, the most common disorders were schizophrenia and other primary psychotic disorders (n = 72, 41%), depressive disorders (n = 19, 11%) and eating disorders (n = 19, 11%). Forty-seven of the articles (27%) did not specify the mental disorder that was studied. For a comprehensive list of study characteristics refer to S2 Table.

### Description of questionnaires

A total of 144 questionnaires were identified that assessed the impact of caregiving. Impact of caregiving concepts were categorized into 15 concept clusters, namely *caregiving burden*, *caregiving needs*, *caregiver service use*, *characteristics of caregivers*, *conceptions of mental illness*, *family impact*, *mental health*, *overall caregiving situation*, *physical health*, *overall health*, *quality of life*, *satisfaction*, *social impact*, *work impact*, and *other caregiving consequences*, (Table 2). Three types of questionnaires were identified based on the original target population, namely “specific mental disorder” (n = 32; 22%) “non-specified mental disorder” (n = 46; 32%) and “other” (n = 67; 46%). The specific mental disorders were autism spectrum disorder (n = 3), eating disorders (n = 3), mood disorders (n = 4), personality disorders (n = 4), and primary psychotic disorders (n = 15), and primary psychotic disorders and personality disorders (n = 2). A total of 20 non-validated questionnaires (13%) were identified that were specifically developed for the purpose of those studies.

**Table 2:** Overview of concept clusters, concepts, and their respective questionnaires, including questionnaires categorization and original target population.

Concept cluster	Concept	Questionnaire (ref.)	Questionnaire type	Specific mental disorder
**Caregiving burden**	Caregiving burden	Caregiver Burden Inventory [38]	Other	-
		Caregiver Strain Questionnaire [39]	Non-specified mental disorder	-
		Eating Disorder Impact Scale [40]	Specific mental disorder	Eating disorders
		Perceived Burden Scale [41]	Non-specified mental disorder	-
		Self-developed visual analogue scale by Heru & Ryan [42]	Specific mental disorder	Mood disorders
		Zarit Burden Interview [43–47]	Other	-
	Caregiver strain	Caregiver Strain Index [48]	Other	-
		Caregiver Strain Questionnaire- Short Form 7 [49]	Non-specified mental disorder	-
		Strain Scale [50]	Other	-
	Objective burden	Care-ED [51]	Specific mental disorder	Eating disorders
		Self-developed questionnaire by Hielscher et al. [52]	Non-specified mental disorder	-
	Self-perceived pressure	Self-Perceived Pressure by Informal Care Scale [53]	Other	-
	Subjective burden	1992–1993 Family Impact Study [54]	Non-specified mental disorder	-
		Burden Assessment Schedule [55]	Specific mental disorder	Primary psychotic disorders
		Caregiver Burden Scale [56]	Other	-
		Schizophrenia Caregiver Questionnaire [57]	Specific mental disorder	Primary psychotic disorders
	Family burden	Burden Assessment Scale [58]	Non-specified mental disorder	-
		Entrevista de Carga Familiar Objetiva y Subjetiva/Objective and Subjective Family Burden Interview [59, 60]	Specific mental disorder	Primary psychotic disorders
		Family Burden and Care Participation Instrument [61]	Non-specified mental disorder	-
		Family Burden Interview Schedule [62]	Non-specified mental disorder	-
		Family Burden Questionnaire [63]	Non-specified mental disorder	-
		Family Burden Questionnaire [64]	Non-specified mental disorder	-
		Family Burden Scale [65]	Specific mental disorder	Primary psychotic disorders
		Interview for Measuring the Burden on the Family [66]	Non-specified mental disorder	-
		Interview Schedule for Families and Relatives of Severely Mentally Ill Persons [67]	Non-specified mental disorder	-
		Perceived Family Burden Scale [68]	Non-specified mental disorder	-
		Self-developed questionnaire Goodman et al. [69]	Specific mental disorder	Personality disorders
	Family burden^a^/ Expressed emotion	Family Problems Questionnaire [70]	Non-specified mental disorder	-
**Caregiving needs**	Caregiver needs	Caregivers’ Needs Assessment for Schizophrenia [71]	Specific mental disorder	Primary psychotic disorders
		Relative’s Cardinal Needs Schedule [72]	Specific mental disorder	Primary psychotic disorders
	Caregiver need for assertive community treatment	Self-developed questionnaire by Sono, Oshima, & Ito [73]	Non-specified mental disorder	-
	Caregiver support service need	Behavioral Risk Factor Surveillance System [74]	Other	-
		General Social Survey Questionnaire [75]	Other	-
	Caregiver unmet needs	Self-developed questionnaire by Chamba et al. [76]	Specific mental disorder	Autism spectrum disorder
	Relative’s need for psychosocial interventions	Relative’s Urgent Needs Schedule- Early Intervention [77]	Specific mental disorder	Primary psychotic disorders
	Family needs	Caregiver Needs Survey [78]	Specific mental disorder	Autism spectrum disorder
		Family Needs Questionnaire [79]	Other	-
**Caregiver service use**	Caregiver service use	Self-developed questionnaire by Perlick, Hohenstein, Clarkin, Kaczynski, & Rosenheck [80]	Specific mental disorder	Personality disorders
	Health care utilization	2010, 2011, and 2013 EU5 National Health and Wellness Survey [81]	Other	-
		Insurance-Medicine-All-Sweden (IMAS) study [82]	Other	-
	Medical care use and expenditure	Medical Expenditure Panel Survey [83]	Other	-
	Mental health service utilization and costs	Client Service Receipt Inventory- Service Receipt section [84]	Other	-
	Utility of possible sources of support	Family Support Scale [85]	Other	-
**Characteristics of caregivers**	Faith behaviors/ practices	Christian Faith Practices Scale [86]	Other	-
	Future intention to care	Intention to Care Scale [87]	Non-specified mental disorder	-
	Prioritization of personal needs	Self- and Sibling-Care Measure [88]	Non-specified mental disorder	-
	Sense of coherence	Sense of Coherence Index [89]	Other	-
**Conceptions of mental illness**	Cognitive representations of mental health problems	Illness Perceptions Questionnaire for Schizophrenia- Relatives’ version [90]	Specific mental disorder	Primary psychotic disorders
	Knowledge about mental illness	Knowledge Measure [91]	Non-specified mental disorder	-
		Mental Health Knowledge Schedule [92]	Other	-
	Mental illness and disorder understanding	Mental Illness and Disorder Understanding Scale [93]	Other	-
	Parent’s assessment of eating behaviors and attitudes	Anorectic Behavior Observation Scale [94]	Specific mental disorder	Eating disorders
	Public conception of mental illness	Self-developed vignette by Link et al. [95]	Other	-
**Family impact**	Adult sibling relationship	Adult Sibling Relationship Questionnaire [96]	Other	-
	Expressed emotion	Family Questionnaire [97]	Specific mental disorder	Primary psychotic disorders
		Family Attitudes Scale [98]	Non-specified mental disorder	.-
	(Patient’s perception of) expressed emotion	Level of Expressed Emotion [99]	Non-specified mental disorder	-
	Family burden/ Expressed emotion^a^	Family Problems Questionnaire [70]	Non-specified mental disorder	-
	Family attitudes towards schizophrenia	Attitudes Towards Schizophrenia Questionnaire for Relatives [100]	Specific mental disorder	Primary psychotic disorders
	Family communication	Family Communication Scale [101]	Specific mental disorder	Primary psychotic disorders / Personality disorders
	Family empowerment	Family Empowerment Scale [102]	Other	-
	Family experiences	Family Experiences Interview Schedule [103]	Non-specified mental disorder	-
	Family functioning	Family Assessment Device [104]	Other	-
		Self-developed visual analogue scale by Heru & Ryan [42]	Specific mental disorder	Mood disorders
		Self-developed visual analogue scale by Heru, Ryan & Vlastos [105]	Specific mental disorder	Mood disorders
	Family quality of life	Family Quality of Life Survey [106]	Non-specified mental disorder	-
	Family role	Role Behavior Inventory [107]	Other	-
	Family strengths	Family Strengths Scale [108]	Other	-
	Parent adjustment	Parent Experience of Chronic Illness [109]	Other	-
**Mental health**	Anxiety symptomology	Beck Anxiety Inventory [110]	Other	-
	Burnout syndrome	Maslach Burnout Inventory- Human Services Survey [111]	Other	-
	Depression	Center for Epidemiologic Studies- Depression [112]	Other	-
		WHO World Health Survey [7]	Other	-
	Depressive symptomology	Beck Depression Inventory [113, 114]	Other	-
		Geriatric Depression Scale [115]	Other	-
	Diagnosable psychiatric disorder	Composite International Diagnostic Interview [116, 117]	Other	-
		General Health Questionnaire [118–122]	Non-specified mental disorder	-
		Goldberg Anxiety and Depression Scale [123]	Other	-
		Mental Health Inventory-5 [124]	Other	-
	Emotional health	General Social Survey Questionnaire [75]	Other	-
	Emotion dysregulation	Difficulties in Emotion Regulation Scale [125]	Other	-
	Emotional disorders/ Depression and anxiety	Hospital Anxiety and Depression Scale [126]	Non-specified mental disorder	-
	Feelings and expression of anger	State-Trait Anger Scale [127]	Other	-
	Grief connected to having a loved one with mental illness	Grief Scale [128]	Non-specified mental disorder	-
	Mental disorder	Techniker Krankenkasse [129]	Other	-
	Memory errors	Prospective and Retrospective Memory Questionnaire [130]	Other	-
	Mental health	Behavioral Risk Factor Surveillance System [74]	Other	-
	Mental wellbeing	Warwick-Edinburgh Mental Wellbeing Scale [131]	Other	-
	Mood state	Profile of Mood States [132]	Non-specified mental disorder	-
	Perceived stress	Perceived Stress Scale [133]	Other	-
	Psychological distress	Brief Symptom Inventory [134]	Other	-
		Depression, Anxiety and Stress Scale [135]	Other	-
		General Symptom Index [136]	Non-specified mental disorder	-
		Kessler Psychological Distress Scale [137]	Non-specified mental disorder	-
		Non-Specific Psychological Distress and Positive Emotions Scale [138]	Other	-
		Symptom Check List Revised [139, 140]	Non-specified mental disorder	-
	Psychological wellbeing	Psychological Wellbeing (PwB) Scale [141]	Other	-
	Stress	General Stress Scale [142]	Other	-
	Subjective distress caused by traumatic events	Impact of Event Scale- Revised [143]	Other	-
	Unresolved grief	Mental Illness Version of the Texas Inventory of Grief [144]	Non-specified mental disorder	-
		Texas Inventory of Grief- Early Intervention [77]	Non-specified mental disorder	-
	Worry	Penn State Worry Questionnaire [145]	Non-specified mental disorder	-
**Overall caregiving situation**	Appraisal of caregiving experience	Caregivers’ and Users’ Expectations of Services- Caregiver version [146]	Non-specified mental disorder	-
		Experience of Caregiving Inventory [22]	Non-specified mental disorder	-
	Awareness of care	Nursing Awareness [147]	Other	-
	Caregiving stress	General Social Survey Questionnaire [75]	Other	-
	Experiences of violence and aggression	Perceptions of Prevalence of Aggression Scale [148]	Non-specified mental disorder	-
	Interaction guilt	Well Sibling Guilt Index (WSIGI) of the Well Sibling Guilt Questionnaire [149]	Non-specified mental disorder	-
	Involvement of caregivers in the consumer’s hospital admission	Self-developed survey by the Private Mental Health Consumer Caregiver Network [150]	Specific mental disorder	Personality disorders
	Safety fears	Self-developed survey by Labrum & Solomon [151]	Non-specified mental disorder	-
	Self-efficacy	General Self-Efficacy Scale [152]	Other	-
**Overall health**	Adverse health status	Insurance-Medicine-All-Sweden (IMAS) Study [82]	Other	-
	Caregiver wellbeing^a^/ Caregiver satisfaction with the support they receive	Caregiver Well-Being and Support (CWS) Questionnaire [153]	Non-specified mental disorder	-
	Disability status	Self-developed questionnaire by Csoboth et al. [154]	Specific mental disorder	Primary psychotic disorders
	General medical symptoms	Wisconsin Longitudinal Study (WLS) Survey [155]	Other	-
	Overall health status	Behavioral Risk Factor Surveillance System [74]	Other	-
		Cornell Medical Index [156]	Other	-
		Medical Outcomes Study Short-Form Health Survey [157–159]	Other	-
		Self-developed questionnaire by Ali, Krevers, & Skärsäter [160]	Non-specified mental disorder	-
	Wellbeing	1992–1993 Family Impact Study [54, 128]	Non-specified mental disorder	-
**Physical health**	Physical health	Behavioral Risk Factor Surveillance System [74]	Other	-
		Physical Health Rating [161]	Other	-
		Self-developed scale by Greenberg et al. [162]	Other	-
	Risk of developing diabetes	Australian Type 2 Diabetes Risk Assessment Tool [163]	Other	-
	Sleep problems	WHO World Health Survey [7, 164]	Other	-
	Somatic symptoms without organic cause	Somatic Symptom Scale [165]	Non-specified mental disorder	-
**Quality of life**	Care-related quality of life	CarerQoL [166]	Other	-
	Health-related quality of life	EuroQoL [167]	Other	-
		Health Utilities Index [168]	Other	-
	Quality of life	Quality of Life Index [169]	Other	-
		Quality of Life Measure [170]	Specific mental disorder	Autism spectrum disorder
		World Health Organization Quality of Life [171–175]	Other	-
**Satisfaction**	Caregiver wellbeing/ Caregiver satisfaction with the support they receive^a^	Caregiver Well-Being and Support (CWS) Questionnaire [153]	Non-specified mental disorder	-
	Caregivers’ perceptions of support from health professionals for them as caregivers	Self-developed survey by the Private Mental Health Consumer Caregiver Network [150]	Specific mental disorder	Personality disorders
	Family satisfaction	Family Satisfaction Scale [176]	Specific mental disorder	Primary psychotic disorders / Personality disorders
		Family Satisfaction Scale [177]	Other	-
	Global life satisfaction	Satisfaction with Life Scale [178]	Other	-
	Marital satisfaction	Marital Adjustment Test [179]	Other	-
		Marital Satisfaction Questionnaire for Older Persons [180]	Other	-
**Social impact**	Affiliate stigma	Affiliate Stigma Scale [181]	Non-specified mental disorder	-
	Depression-related stigma	Self-developed scale by Griffiths et al. [182]	Specific mental disorder	Primary psychotic disorders
	Social isolation	Friendship Scale [183]	Other	-
	Social network	Social Network Questionnaire [70]	Non-specified mental disorder	-
	Social participation	Wisconsin Longitudinal Study (WLS) Survey [155]	Other	-
	Social rejection	Kreisman’s Family Rejection Scale [184]	Specific mental disorder	Primary psychotic disorders
**Work impact**	Labor force participation	Self-developed scale by Csoboth et al. [154]	Specific mental disorder	Primary psychotic disorders
	Paid and unpaid work impairment	Work Productivity and Impairment Questionnaire [185]	Other	-
	Work productivity loss	Insurance-Medicine-All-Sweden (IMAS) study [82]	Other	-
**Other caregiving consequences**	Caregiving consequences	Additional Involvement Evaluation Questionnaire Modules [186]	Specific mental disorder	-
		Involvement Evaluation Questionnaire [186–188]	Specific mental disorder	Primary psychotic disorders
	Caregiving reward	Self-developed visual analogue scale by Heru & Ryan [42]	Specific mental disorder	Mood disorders
	Difficulty and adversity that caregivers experience in trying to manage social and family life, finances, and control over their personal lives	Family Life Difficulty Scale [184]	Non-specified mental disorder	-
	Experienced challenges	Self-developed questionnaire by Corsentino et al. [189]	Non-specified mental disorder	-
	Financial difficulty	Wisconsin Longitudinal Study (WLS) Survey [155]	Other	-
	Instrumental costs	Self-developed questionnaire by Lohrer, Lukens, & Thorning [190]	Non-specified mental disorder	-
	Psychiatric patient’s social behavior and its impact upon significant others	Social Behavior Assessment Scale [191]	Non-specified mental disorder	-
	Subjective perception of negative and positive aspects of caregiving	COPE Index [192, 193]	Other	-
	Stress-related growth	Stress-Related Growth Scale-Revised [194]	Other	-

### Impact of caregiving

#### Conceptualization and operationalization

The concept clusters are described in detail below. The dimensions and operationalization of each concept (cluster), including all references, can be found in the meta-summaries reported in the S2 File.

*Caregiving burden*. *Caregiving burden* assesses the strain and negative consequences of caregiving, with objective and subjective burden emerging as two distinct conceptualizations. Objective burden is the negative occurrences that resulted from caregiving, including the interruption of personal time, missing work, and financial strain. Subjective burden are the affective responses exhibited by the informal caregiver due to their caregiving, including subjective worry and distress. *Caregiving burden* was assessed for individual caregivers and families that took on a caregiving role. From 28 questionnaires, a total of 70 dimensions were identified. *Caregiving burden* was operationalized into overall caregiving burden, the impact of caregiving on their daily lives and wellbeing, the caregiver-care recipient relationship, and self-rated incompetence. Overall caregiving burden was assessed as non-specific evaluations of objective and subjective burden and the duration of various caregiving tasks. The impact of caregiving included negative and positive consequences that affected the caregiver’s appraisal of their caregiving situation, their care recipient, and their everyday lives. The effect of caregiving on the caregiver’s everyday life was widespread and included their health, wellbeing, financial situation, work, leisure, and relationships. The effect of caregiving on the family focused on the dynamics of the household, the relationship with their partner, and the impact on individual family members, especially the children. The framing of the caregiver-care recipient relationship was negative and focused on tensions that existed due to the care recipient’s condition and the caregiving situation. Caregiver incompetence was operationalized as the caregiver’s valuation of their caregiving abilities.

*Caregiving needs*. *Caregiving needs* refers to the desires and necessities of the informal caregiver due to their caregiving responsibilities. These needs were identified for the family, relatives and other individuals that took on the caregiving role. A total of nine questionnaires operationalized *caregiving needs* into 25 dimensions. Needs were identified in relation to the caregiving situation and the personal life of the caregiver. Caregiving situation needs were the needs for caregiver support and other needs related to the care recipient’s symptoms and behavior. Caregiver support was identified for different caregiving tasks and caregiver support services. Additionally, caregiver needs in their personal life were identified and operationalized for the caregiver’s social life, work/study, and finances.

*Caregiver service use*. *Caregiver service use* is conceptualized as the informal caregiver’s utilization of informal and formal services due to their informal care provision. *Caregiver service use* was operationalized by six questionnaires into six dimensions. Overall service use was identified as a general service use dimension that considered service use from medical services, community-based and criminal justice service contacts, and different forms of caregiver support use. Caregiver support services included assistance provided to the caregiver on behalf of a variety of informal and formal community-based sources. Medical care use were dimensions that assessed specialized health service utilization (i.e., mental, and physical health services) and primary care service utilization.

*Characteristics of caregivers*. *Characteristics of caregivers* are concepts that defined the daily lives of informal caregivers and were impacted by caregiving. Four questionnaires operationalized these concepts into 19 dimensions. These dimensions assessed different aspects of the informal caregiver’s daily life, their caregiving intentions for the future, and sense of coherence. The informal caregiver’s daily life concerned stressful events that could occur, their religion, their involvement in the community, and self-care priorities. The intention to provide care was assessed for different caregiving tasks that the individual would be willing to perform in the future. Sense of coherence refers to the adaptive dispositional orientation of a person that enables them to cope with adverse experiences.

*Conceptions of mental illness*. *Conceptions of mental illness* is defined as the informal caregiver’s personal understanding and opinions of mental illness and their care recipient and considered how this was affected by caregiving. This was conceptualized as the informal caregiver’s overall knowledge and their assessment of disease-related behaviors and attitudes. A total of six questionnaires was operationalized into 25 dimensions. Knowledge and understanding of mental disorders were the caregiver’s understanding of the different stages of the patient’s disease trajectory. Stigma emerged as a separate dimension, which concerned the negative or false personal beliefs that the caregiver may have about mental illness or individuals suffering from a mental illness. Personal blame assessed the caregiver’s attribution of blame directed towards themselves and the care recipient for the mental disorder.

*Family impact*. *Family impact* is conceptualized as the positive and negative consequences that caregiving and the care recipient have on the family unit. These concepts assessed the family’s dynamics and the family caregiver’s attitudes towards specific mental disorders. Sixteen questionnaires operationalized *family impact* into 42 dimensions. The dimensions assessed family functioning and communication, expressed emotion, and characterized the family’s caregiving situation. Different aspects relating to family functioning were identified, such as the family’s ability to problem solve and family cohesion. Expressed emotion is a measure of the family environment based on how family members spontaneously talk about their mentally ill relative [195]. The caregiving situation was characterized by the caregiving tasks that were performed and the family’s responses to caregiving and the care recipient.

*Mental health*. *Mental health* refers to informal caregiver’s diagnosable psychiatric disorders, psychological wellbeing and distress, and emotional wellbeing measures that were impacted by caregiving. Thirty-three questionnaires assessed mental health concepts and operationalized them into 65 dimensions. Several psychiatric disorders were operationalized, namely burnout, mood disorders, anxiety disorders, obsessive compulsive disorders, and primary psychotic disorders. Dimensions assessing subjective sense of personal worth were found that assessed the informal caregiver’s purpose in life and personal growth. Negative dimensions relating to emotional wellbeing were identified, such as grief and stress. Environmental mastery is a dimension that assesses the informal caregiver’s self-rated sense of control and competence in managing their external environment and making effective use of their surrounding opportunities. Overall psychological measures were operationalized as either negative (i.e., psychological distress) and positive dimensions (i.e., psychological wellbeing).

*Overall caregiving situation*. *Overall caregiving situation* refers to the informal caregiver’s appraisal of their caregiving experience and their involvement in the care recipient’s care. A total of 9 questionnaires assessed the *overall caregiving situation*. From these questionnaires, 29 dimensions were identified. These dimensions assessed the informal caregiver’s appraisal of their caregiving abilities and situation, caregiver support, and care recipient characteristics. The informal caregiver’s appraisal of their caregiving abilities was largely comprised of self-efficacy. Self-efficacy is the informal caregiver’s perceived ability to succeed in specific situations. Caregiver’s appraisal of their caregiving situation was operationalized into negative and positive dimensions that assessed specific aspects of their caregiving situation, such as interaction guilt and good aspects of the relationship. Caregiving support is the availability and quality of particular caregiver support services. The informal caregiver’s appraisal of the care recipient included negative behaviors, symptoms, and aggression exhibited by the care recipient.

*Physical health*. *Physical health* is conceptualized as the caregiver’s overall physical health and specific physical ailments that were impacted by caregiving. From six questionnaires, a total of 14 dimensions were identified. *Physical health* was operationalized into general health-related characteristics, overall physical health, and physical conditions. General health-related characteristics are factors that may influence the caregiver’s overall physical health, including lifestyle and demographic measures. Overall physical health is the caregiver’s self-rated poor physical health days. Physical conditions are a range of disorders across the major human bodily systems.

*Overall health*. *Overall health* is conceptualized as the informal caregiver’s general health status, functioning, and wellbeing due to caregiving. A total of 9 questionnaires assessed *overall health* and was operationalized into 41 dimensions. The dimensions included the caregiving situation and the informal caregiver’s overall health status. In relation to the caregiving situation, negative characteristics of the care recipient, day-to-day life as a caregiver, safety, and the caregiver-care recipient relationship were identified as relevant domains. Overall health was operationalized as the caregiver’s overall functioning, health, and social wellbeing.

*Quality of life*. *Quality of life* is the overall quality of life measures that were impacted by caregiving. Quality of life was conceptualized as general quality of life measures and quality of life measures related to the care and health domains. Six questionnaires operationalized *quality of life* into 24 dimensions. The domains assessed the caregiver’s environment, which refers to their financial resources, residence, socioeconomic status, and physical environment. The family of the caregiver was evaluated, wherein the dimensions considered the interactions between family members and their overall happiness. Caregiver health was operationalized into domains that assessed their ability to function in terms of their mental, physical, and overall health.

*Satisfaction*. *Satisfaction* is defined as a measure of the informal caregiver’s overall fulfilment of their expectations, needs, and wishes in relation to their caregiving situation and other aspects of their life. The concepts were evaluated for families and other individuals that took on the caregiving role. Seven questionnaires operationalized *satisfaction* into 21 dimensions. Satisfaction with life was operationalized as the informal caregiver’s life being close to ideal, having the important things that they want in life, and having no desire to change anything if they could live their life over. Satisfaction with caregiver support was the caregiver’s satisfaction with respite care, their support from different health providers, and caregiver’s involvement in the care recipient’s treatment. Family satisfaction is satisfaction relating to the functioning of the family as a whole and between spouses.

*Social impact*. *Social impact* are the consequences of caregiving on the informal caregiver’s social life and was conceptualized as experienced stigma, social participation, and negative social impact. The concepts were operationalized by six questionnaires with a total of 16 dimensions. The dimensions included the nature of social contacts, social support and participation, and stigma. The nature of social contacts was framed as negative social consequences and the frequency of contact. Negative social consequences included social isolation and rejection. Two different types of social support were identified, namely emotional and practical social support. Social participation evaluated engagement in activities and community-based organizations, such as charitable organizations.

*Work impact*. *Work impact* refers to the impact that caregiving had on the informal caregiver’s paid and unpaid work. Three questionnaires assessed work impact-related concepts. From these questionnaires, eight dimensions were identified. These dimensions included productivity loss, labor force participation, and sources of income. Two types of productivity loss were operationalized, namely absenteeism and presenteeism.

*Other caregiving consequences*. *Other caregiving consequences* includes impact of caregiving measures that were not domain specific. A total of 10 questionnaires were identified. These concepts were operationalized into 30 dimensions. These dimensions classified consequences based on who was affected by the caregiving situation. Other consequences for the caregiver were operationalized by questionnaires as negative and positive framing of consequences and included consequences for their daily lives, self-development, the relationship with the care recipient, and the caregiving situation.

#### Trends in concept cluster use

The five most frequently assessed concept clusters were *mental health* (n = 75), *caregiving burden* (n = 65), *other caregiving consequences* (n = 30), *family impact* (n = 22), and *overall health* (n = 22). *Mental health* and *caregiving burden* had distinct increases in assessment over the years compared to other concept clusters. The other concept clusters had no clear assessment trends, with some random assessment spikes.

#### Use of concept clusters per diagnosis group

Concept use was determined for all diagnosis groups (Table 3). The distribution of concept use differed per diagnosis group. Select diagnosis groups, namely schizophrenia and other primary psychotic disorders, eating disorders, bipolar disorders, depressive disorders, and autism spectrum disorders, employed a broad scope in impact of caregiving. The other diagnosis groups only used a limited number of concept clusters. For anxiety disorders, autism spectrum disorder, bipolar and related disorders, eating disorders, personality disorders, schizophrenia and other primary psychotic disorders, and trauma- and stressor-related disorders, the most assessed concept cluster was *mental health*. *Quality of life* was the most assessed concept cluster for anxiety disorders and obsessive compulsive and related disorders. *Caregiving burden* was the top concept cluster for attention deficit hyperactivity disorder, depressive disorders, and substance-related and addictive disorders.

**Table 3:** Use of concept clusters per diagnosis group.

Diagnosis group	Number of times assessed *n*															
	Caregiving burden	Caregiving needs	Caregiver service use	Characteristics of caregivers	Conceptions of mental illness	Family impact	Mental health	Overall caregiving situation	Physical health	Overall health	Quality of life	Satisfaction	Social impact	Work impact	Other caregiving consequences	Total
**Anxiety disorders**	0	0	0	0	0	0	1	0	0	0	1	0	0	0	0	2
**ADHD**	1	0	0	0	0	0	0	0	0	0	0	0	0	0	0	1
**ASD**	2	2	2	0	0	1	10	0	2	1	1	0	1	0	1	23
**Bipolar and related disorders**	8		2	0	0	5	9	1	0	6	0	1	0	0	7	39
**Depressive disorders**	6	0	1	0	0	3	5	1	0	5	3	0	0	0	6	30
**ED**	9	2	0	0	1	2	10	2	0	4	1	0	0	0	4	35
**Obsessive- compulsive and related disorders**	1	0	0	0	0	1	0	0	0	0	2	0	0	0	0	4
**Personality disorders**	5	0	0	1	0	0	6	1	0	0	0	1	0	0	0	14
**Schizophrenia and other primary psychotic disorders**	27	3	5	0	5	8	34	7	2	8	11	2	5	6	7	130
**Substance-related and addictive disorders**	2	0	0	0	0	1	1	1	0	0	1	0	0	0	1	7
**Trauma- and stressor-related disorders**	0	0	0	0	0	0	2	0	0	1	0	1	0	0	0	4

*Notes*. ADHD = attention deficit hyperactivity disorder; ASD = autism spectrum disorder; ED = eating disorders

## Discussion

This is the first systematic literature review to generate an overview of the questionnaires and concepts used to assess the impact of caregiving. We found that caregiving has a widespread impact on the lives of informal caregivers; however, the assessment of impact was often limited to domain specific measures. Moreover, there was a high degree of variability in the conceptualization and operationalization of the impact of caregiving. Despite the increasing number of publications in this field of research, there is no clear consensus on the use of concepts and questionnaires. The results of the review indicate that over the last 15 years, a variety of concepts were used to assess the impact of caregiving, irrespective of the type of mental disorder and timeframe. The variability can partly be accredited to the terminology used to define the respective area of impact. When concepts were clustered, the impact of caregiving was conceptualized into 15 concept clusters.

In our study, we found that the current conceptualization and operationalization of caregiving impact does not align with theoretical frameworks in the field. The current caregiving research paradigm aims to understand the experience of having a relative with a mental disorder [21] and allows for the negative and positive assessment of informal caregiving [196]. These theoretical models include theories of resilience [197, 198] and stress-coping approaches [199] and form the basis of some of the questionnaires that were identified in the review, such as the Experience of Caregiving Inventory [22]. These respective concepts were classified as *other caregiving consequences* and *overall caregiving situation* and address the shortcomings of concepts that are not grounded in psychological and social theories (i.e., *caregiving burden*). *Caregiving burden* is critiqued for being difficult to operationalize [21, 22] and unable to recognize the rewarding aspects of caregiving [200]. However, as evidenced by our review, concepts such as *caregiving burden* remain popular in caregiving research. This could be due to the historical use of this concept in caregiving research [18] and methodological limitations of studies that support the negative assessment of informal caregiving [196].

The assessment of the caregiving impact differed across disease groups, with certain disease groups assessing a range of concepts and others only assessing a limited number of concepts. Further research is needed to determine whether the impact of caregiving is truly less widespread for particular disease groups. This trend appeared to correspond with the number of times that a disease group was studied. Schizophrenia and other primary psychotic disorders were the most studied disease group in our review and have received academic attention since the start of deinstitutionalization [18]. This may be due to the symptomology of primary psychotic disorders [201] and disease-related stigma [202, 203]. Symptomology of disorders can have a significant impact on caregivers, regardless of diagnosis [204]. For example, positive symptoms of schizophrenia patients are received differently by caregivers than negative symptoms [201]. Similarly, *caregiver burden* has been found to fluctuate due to varying behavior exhibited by bipolar patients across manic and hypomanic episodes [205]. Nonetheless, peer-reviewed literature is generally focused on investigating the impact of caregiving for specific mental disorders and not symptoms [206–208].

The sensitivity of identified questionnaires may not be sufficient to detect the impact of caregiving for this study population, because almost half of the questionnaires were not originally developed for psychiatric disorders. The lived experiences of caregivers for patients with mental disorders are complex [209] and differ to that of other informal caregivers [33, 210]. They are often left vulnerable to structural discrimination, which can adversely affect their social interactions and access to certain social roles [211–214]. Likewise, the symptoms of severe mental disorders have been identified as strong predictors of depression and anxiety [215]. Caregivers state that they often have difficulties understanding the symptoms and behavior of their loved ones [216]. They are also required to navigate fragmented medical, legal, and governmental systems to ensure that their loved ones receive adequate medical care. These formal systems often neglect the informal caregiver and undervalue their role [209, 217]. Currently, limited data is available to determine the acceptability, reliability, and validity of questionnaires for this caregiving population [19]. However, the comparability of questionnaires across studies and conditions should also be considered when selecting a questionnaire.

### Future research recommendations

The results of this review give an initial insight into the operationalization and conceptualization of the impact of caregiving; however, further research is needed to: (a) ensure the completeness of concepts and dimensions, (b) validate the formulation of our concept clusters, (c) explore the prioritization of concepts by informal caregivers, (d) determine whether the lived experiences of this caregiving population warrant the use of specific questionnaires, and (e) investigate how the conceptualization and operationalization of caregiving impact may differ across diagnosis groups.

### Methodological limitations

There are some limitations that should be explored. Firstly, the paper should be scrutinized for categorical bias. Categorical bias could have occurred during the generation of the concept clusters because the process required a degree of personal interpretation. Secondly, the transferability of our findings to other cultural settings is limited, due to the exclusion of non-English publications and non-OECD research. The cultural norms and perceptions concerning informal caregiving has been found to vary greatly across countries and could have impacted our identification of concepts [19]. Thirdly, studies and questionnaires could not be identified for some mental disorders. These factors may have affected the selection of concepts and their respective operationalization. Lastly, the generalizability of our study was limited to adult caregivers and care recipients. The age of the care recipient and caregiver is a factor that not only alters the caregiving experience, but also plays a role in the impact of caregiving. For example, concepts such as parentification are not relevant for adults and was not included in our concept list but should be considered for minors [218].

## Supporting information

S1 Table PRISMA checklist. (DOCX)

S2 Table Study characteristics of component articles. (DOCX)

S1 File Complete search string per database. (DOCX)

S2 File Impact of caregiving meta-summaries. NR = not reported.
(DOCX)
