# Supplementary material for: Assessing the impact of caregiving on informal caregivers of adults with a mental disorder in OECD countries: A systematic literature review of concepts and their respective questionnaires
Source: PLoS One. 2022 Jul 8;17(7):e0270278. doi: 10.1371/journal.pone.0270278 (PMC9269485; doi:10.1371/journal.pone.0270278)
Supplement: S2 Table — (DOCX) [file pone.0270278.s002.docx]

| **Article ref.** | **First author, year** | **Country** | **Study population** | **Care receiver diagnosis** | **Study design** | **Questionnaires employed** |
| --- | --- | --- | --- | --- | --- | --- |
| **[1]** | **Heru, 2004** | U.S.A. | Relatives of inpatients at a psychiatric hospital | Bipolar and related disorders; Depressive disorders | Prospective cohort study | Family Assessment Device;  Caregiver Strain Index;  Self-developed visual analogue scales by Heru & Ryan [41]; Medical Outcomes Study Short-Form Health Survey |
| **[2]** | **Ozlu, 2015** | Turkey | Relatives of patients who have been followed up as outpatients and were hospitalized during acute aggravations | Schizophrenia and other primary psychotic disorders | Cross-sectional study | Zarit Burden Interview |
| **[3]** | **Padierna, 2013** | Spain | Relatives of outpatients in specialized eating disorder clinics | Eating disorders | Cross-sectional study | Involvement Evaluation Questionnaire |
| **[4]** | **Zendjidjian, 2012** | France | Relatives or friends who provided the most support or assistance to patients in the psychiatric departments of a French public teaching hospital | Bipolar and related disorders; Depressive disorders | Cross-sectional study | Medical Outcomes Study Short-Form Health Survey |
| **[5]** | **Pérez, 2018** | Spain | Caregivers of people included in the public system of Mental Health care in the Valencian Community | Non-specified mental disorders | Cross-sectional study | Zarit Burden Interview |
| **[6]** | **Winn, 2007** | England | Relatives, friends, and others involved in providing care to adolescents (aged 13-20 years) who received care and were referred for treatment for bulimia nervosa between September 2000 and May 2003 | Eating disorders | Cross-sectional study | General Health Questionnaire |
| **[7]** | **Flyckt, 2013** | Sweden | Caregivers of patients above the age of 18 and in need of continuous long-term antipsychotic medication for functional psychoses | Schizophrenia and other primary psychotic disorders | Cross-sectional study | EQ-5D; Work Productivity and Impairment Questionnaire; CarerQoL; COPE Index |
| **[8]** | **Foldemo, 2005** | Sweden | Parents of outpatients aged 18 years and older who visited the psychiatric outpatient clinic at the University Hospital of Linköping, Sweden in 2001 | Schizophrenia and other primary psychotic disorders | Cross-sectional study | Quality of Life Index |
| **[9]** | **Gonçalves-Pereira, 2013** | Portugal | Primary caregivers of outpatients from mental health facilities | Schizophrenia and other primary psychotic disorders | Cross-sectional study | Experience of Caregiving Inventory; General Health Questionnaire; Involvement Evaluation Questionnaire |
| **[10]** | **Hanzawa, 2009** | Korea; Japan | Participants of local family groups for families of people with mental disorders | Schizophrenia and other primary psychotic disorders | Cross-sectional study | Impact of Event Scale- Revised; Mental Illness and Disorder Understanding Scale; Zarit Burden Interview; Self-developed scale by Griffiths et al. [181]; Self-developed vignette by Link et al. [94] |
| **[11]** | **Khanna, 2011** | U.S.A. | Primary caregivers of children (≥18 years) that were registered with the West Virginia Autism Training Center | Autism spectrum disorder | Cross-sectional study | Medical Outcomes Study Short-Form Health Survey |
| **[12]** | **Magliano, 2005** | Italy | Key relatives (i.e., those relatives who had spent the highest number of hours in contact with patients during the last year) of the first consecutive 25 subjects who had received a DSM-IV diagnosis of schizophrenia | Schizophrenia and other primary psychotic disorders | Cross-sectional study | Social Network Questionnaire ; Family Problems Questionnaire |
| **[13]** | **Mizuno, 2012** | Japan | Families of persons with schizophrenia from two family associations of persons with mental illness and the outpatient departments of three psychiatric hospitals in two prefectures near Tokyo, Japan | Schizophrenia and other primary psychotic disorders | Cross-sectional study | World Health Organization Quality of Life Questionnaire |
| **[14]** | **Möller-Leimkühler, 2005** | Germany | Relatives of inpatients first hospitalized for a schizophrenic or depressive episode within the Research Networks of Schizophrenia and Depression, who lived with them or had a facial contact of at least 15 hours per week | Schizophrenia and other primary psychotic disorders | Prospective cohort study | Family Burden Questionnaire |
| **[15]** | **Molyneux, 2008** | Ireland | Primary caregivers of patients who were consecutively referred to the Psychiatry of Old Age Service over a 24-month period and lived at home in the general community | Non-specified mental disorders | Cross-sectional study | Geriatric Depression Scale; Strain Scale |
| **[16]** | **Kingston, 2016** | England | Self-identified caregivers that had regular weekly contact with the care recipient | Schizophrenia and other primary psychotic disorders | Cross-sectional study | Impact of Event Scale- Revised |
| **[17]** | **Wingrove, 2019** | Australia | Partners, parents, and siblings providing care to young people (12-25 years) from youth mental health services in the region, local caregivers groups’ newsletters, and relevant Facebook pages | Non-specified mental disorders | Cross-sectional study | Perceived Stress Scale; Caregiver Strain Questionnaire- Short Form 7: Adult Caregiver |
| **[18]** | **Ranieri, 2017** | Ireland | Caregivers of involuntarily and voluntarily admitted patients | Non-specified mental disorders | Cross-sectional study | General Health Questionnaire; Involvement Evaluation Questionnaire |
| **[19]** | **Magliano, 2009** | Italy | Relatives of outpatients with a clinical diagnosis of bipolar disorder according to DSM-IV who had been in contact with the local mental health center for at least 6 months | Bipolar and related disorders | Cross-sectional study | Family Problems Questionnaire |
| **[20]** | **Mitsonis, 2012** | Greece | Caregivers of patients with chronic schizophrenia, who underwent a monthly follow-up at the outpatients’ clinic of the Psychiatric Hospital of Athens | Schizophrenia and other primary psychotic disorders | Cross-sectional study | Symptom Check List Revised |
| **[21]** | **Elmståhl, 2018** | Sweden | Caregivers from a general population study ‘Good Aging in Skåne’, which consisted of data from 4459 men and women 60 years and older from rural and urban areas | Depressive disorders | Cross-sectional study | Caregiver Burden Scale |
| **[22]** | **Babinski, 2020** | U.S.A. | Mothers of adolescents and young adults who were diagnosed with ADHD in childhood at the Western Psychiatric Institute and Clinic in Pittsburgh, Pennsylvania from 1987 to 1996 | Attention deficit hyperactivity disorder | Prospective cohort study | Caregiver Strain Questionnaire |
| **[23]** | **Bailey, 2014** | Australia | Caregivers for patients with borderline personality disorder who were actively engaged in seeking support, education, and information from local and international forums | Personality disorders | Cross-sectional study | Mental Health Inventory-5; Grief Scale; Difficulties in Emotion Regulation Scale; Burden Assessment Scale |
| **[24]** | **Caqueo-Urizar, 2006** | Chile | Family members caring for patients with schizophrenia who attended Mental Health Outpatient Centers in the city of Arica, Chile | Schizophrenia and other primary psychotic disorders | Cross-sectional study | Zarit Burden Interview |
| **[25]** | **Caqueo-Urizar, 2012** | Chile | Caregivers of patients with schizophrenia that were receiving treatment at the Mental Health Services in the city of Arica, Chile | Schizophrenia and other primary psychotic disorders | Case-control study | Zarit Burden Interview |
| **[26]** | **Scheirs, 2007** | Netherlands | Caregivers participating in support groups for relatives and caregivers of patients with borderline personality disorder | Personality disorders | Case-control study | Symptom Check List Revised |
| **[27]** | **Blanthorn-Hazell, 2018** | U.K.; Germany; Spain | Caregivers of patients who were currently being treated in a community-based setting and experienced at least one episode of agitation within the last 12 months | Bipolar and related disorders; Schizophrenia and other primary psychotic disorders | Cross-sectional study | Involvement Evaluation Questionnaire |
| **[28]** | **Van Wijngaarden, 2004** | Netherlands | Relatives, friends, partners, and other caregivers of patients from the inpatient and outpatient department of an Academic Medical Center and the outpatient departments of two mental hospitals specialized in the treatment of depression | Depressive disorders | Cross-sectional study | Involvement Evaluation Questionnaire |
| **[29]** | **Jansen, 2015** | Denmark | Caregivers for people recently diagnosed with schizophrenia and enrolled for treatment within Region Zealand in Denmark | Schizophrenia and other primary psychotic disorders | Cross-sectional study | General Health Questionnaire |
| **[30]** | **Ostman, 2004** | Sweden | Spouses, parents, other family members, and non-relatives of patients who had recently been involuntarily and voluntarily admitted to a hospital for their mental health condition | Non-specified mental disorders | Cross-sectional study | Family Burden and Care Participation Instrument |
| **[31]** | **Parks, 2018** | Spain | Parents of children and adolescents recruited from inpatient and outpatient services | Eating disorders; Substance-related and addictive disorders | Cross-sectional study | Experience of Caregiving Inventory |
| **[32]** | **Perlick, 2007** | U.S.A. | Family members or friends who served as the primary caregiver- supported the patient financially, played an active role in the patient´s treatment, and would be contacted by treatment staff in case of an emergency | Bipolar and related disorders | Prospective cohort study | Social Behavior Assessment Scale |
| **[33]** | **Kızılırmak, 2016** | Turkey | Primary caregivers living with the patient who were outpatients or inpatients from two psychiatric research hospitals in Istanbul | Non-specified mental disorders | Cross-sectional study | General Health Questionnaire; Burden Assessment Scale |
| **[34]** | **Koutra, 2014** | Greece | Primary caregivers of patients from the Psychiatric Clinic of the University Hospital of Heraklion, Crete, Greece. The patients had to have been out of the hospital for at least 6 weeks and considered stabilized by their treating psychiatrist | Schizophrenia and other primary psychotic disorders; Bipolar and related disorders | Cross-sectional study | General Health Questionnaire; Family Questionnaire; Family Burden Scale; Family Communication Scale; Family Satisfaction Scale |
| **[35]** | **Las Hayas, 2014** | Spain | Primary caregivers of patients that received treatment from the Eating Disorders Outpatient Clinic of the Psychiatric Services at Galdakao-Usansolo Hospital and Ortuella Mental Health Center in Bizkaia, Spain | Eating disorders | Prospective cohort study | Medical Outcomes Study Short-Form Health Survey |
| **[36]** | **de la Rie, 2005** | Netherlands | Family caregivers of eating disorder patients | Eating disorders | Cross-sectional study | Medical Outcomes Study Short-Form Health Survey |
| **[37]** | **Sagut, 2016** | Turkey | Caregivers of patients with first episode psychosis and chronic psychosis who were followed-up at the psychiatric outpatient unit of a university hospital, psychiatric outpatient center of a training and research hospital, and the outpatient unit of a psychiatric hospital | Schizophrenia and other primary psychotic disorders | Cross-sectional study | Caregiver Burden Inventory |
| **[38]** | **Salaberria, 2015** | Spain | Adult immigrants and relatives of patients with a mental disorder that attended a psychological support program | Non-specified mental disorders | Cross-sectional study | Symptom Check List Revised |
| **[39]** | **Rane, 2012** | England | Primary caregivers of patients discharged from a tertiary in-patient service specializing in the management of treatment-resistant mood disorders | Depressive disorders | Case-control study | Involvement Evaluation Questionnaire |
| **[40]** | **Reinares, 2006** | Spain | Family caregivers of patients that participated in the Bipolar Disorders Program of the Hospital Clinic of Barcelona. Patients were required to fulfill DSM-IV criteria for bipolar I or bipolar II disorder, being euthymic for at least 3 months and aged between 18 and 65 years. Caregivers had to be a significant and influential person in the care recipient´s life and have daily contact with them and be involved in their care | Bipolar and related disorders | Cross-sectional study | Social Behavior Assessment Schedule |
| **[41]** | **Ribé, 2017** | Spain | Primary caregiver who was defined as the person that spent the most time caring for the care recipient | Schizophrenia and other primary psychotic disorders | Cross-sectional study | World Health Organization Quality of Life Questionnaire |
| **[42]** | **Sücüllüoğlu Dikici, 2019** | Turkey | Caregivers of patients who lived with the patients for at least one year | Obsessive- compulsive and related disorders | Cross-sectional study | World Health Organization Quality of Life Questionnaire |
| **[43]** | **Nordstroem, 2017** | Australia; Czech Republic; France; Italy; Russian Federation; Spain; Turkey | Family members, relatives, or friends aged ≥18 years acting in an informal or unpaid capacity and spent at least 4 hours week with the patient | Schizophrenia and other primary psychotic disorders | Cross-sectional study | Schizophrenia Caregiver Questionnaire |
| **[44]** | **Schwarte, 2017** | Germany | Parents of female patients aged 11-18 years with first-onset anorexia nervosa. | Eating disorders | Prospective cohort study | Beck Depression Inventory; Family Questionnaire |
| **[45]** | **Martín, 2015** | Spain; The Netherlands; Denmark; The United Kingdom | Caregivers of patients diagnosed with mental disorders that were being treated in psychiatric hospitals and centers | Eating disorders; Depressive disorders; Schizophrenia and other primary psychotic disorders | Cross-sectional study | Involvement Evaluation Questionnaire |
| **[46]** | **Martorell, 2011** | Spain | Caregivers of patients with ICD-10 diagnosis of schizophrenia from Community Mental Health Sites in Barcelona, Madrid, Granada, and Navarra | Schizophrenia and other primary psychotic disorders | Cross-sectional study | Entrevista de Carga Familiar Objetiva y Subjetiva/Objective and Subjective Family Burden Interview |
| **[47]** | **Skundberg-Kletthagen, 2016** | Norway | Relatives of inpatients hospitalized in a hospital trust in Norway | Depressive disorders | Cross-sectional study | Burden Assessment Scale |
| **[48]** | **Lloyd, 2010** | England | British North Indian Sikh and white British parents of a child diagnosed with schizophrenia who served as the primary caregiver (i.e., parent with the most contact) | Schizophrenia and other primary psychotic disorders | Cross-sectional study | Perceived Family Burden Scale |
| **[49]** | **Chen, 2011** | U.S.A. | Caregivers of a family member with a DSM-III-R diagnosis of schizophrenia or affective disorder | Schizophrenia and other primary psychotic disorders; Depressive disorders; Bipolar and related disorders | Cross-sectional study | 1992-1993 Family Impact Study; Center for Epidemiologic Studies- Depression |
| **[50]** | **Coomber, 2013** | Australia | Participants were 20 matched pairs of individuals diagnosed with an eating disorder and their caregiver | Eating disorders | Cross-sectional study | General Health Questionnaire; Eating Disorder Symptom Impact Scale |
| **[51]** | **Cummings, 2014** | U.S.A. | Family caregivers of older clients who had been diagnosed with a severe mental illness and provided instrumental or emotional support to them within the last month | Schizophrenia and other primary psychotic disorders; Bipolar and related disorders; Depressive disorders | Cross-sectional study | Brief Symptom Inventory |
| **[52]** | **Ruiz-Robledillo, 2016** | Spain | Parents of individuals clinically diagnosed with an eating disorder. Parents were primary caregivers from a clinic in Valencia specialized in the treatment of people with eating disorders and lived in the same home as them for at least 2 years before the study | Eating disorders | Cross-sectional study; Case control study | General Health Questionnaire; Zarit Burden Interview |
| **[53]** | **Day, 2020** | Australia | Family caregivers were recruited through invitations posted on various mental health websites that provide information and support on narcissism | Personality disorders | Cross-sectional study | Mental Health Inventory- 5; Grief Scale; Perceived Burden Scale; Burden Assessment Scale |
| **[54]** | **Depestele, 2016** | Belgium | Parents of female patients aged 14-25 years that were admitted to a specialized treatment unit for eating disorders | Eating disorders | Cross-sectional study | Experience of Caregiving Inventory |
| **[55]** | **Dols, 2018** | Netherlands | Primary caregivers of patients aged 60 years and older that were recruited from a mental health organization in 2012 | Bipolar and related disorders | Cross-sectional study | Zarit Burden Interview; Self-Perceived Pressure by Informal Care Scale |
| **[56]** | **Kirtley, 2019** | England | Caregivers (i.e., parents or partners) of a family member who met the diagnostic criteria for borderline personality disorder or another mental health problem. Caregivers were defined as those that have regular contact with their relative and are involved in offering support financially, emotionally, socially, and/or physically. | Non-specified mental disorders | Cross-sectional study | Burden Assessment Scale |
| **[57]** | **Linacre, 2015** | England | Participants were members of a caregiver support group | Eating disorders | Cross-sectional study | Medical Outcomes Study Short-Form Health Survey |
| **[58]** | **Velligan, 2019** | U.S.A. | Adult caregivers for a patient diagnosed with schizophrenia (time since diagnosis ≥1 year(s) and aged ≥19 years old). Caregivers were required to care for the recipient for ≥1 years, and they had to spend ≥20 hours per week providing care of which 4 hours consisted of direct care | Schizophrenia and other primary psychotic disorders | Cross-sectional study | Schizophrenia Caregiver Questionnaire |
| **[59]** | **Yu, 2018** | U.S.A. | Caregivers of patients with autism spectrum disorder that were two years pre- or post-graduation from high school | Autism spectrum disorder | Cross-sectional study | Caregiver Strain Questionnaire |
| **[60]** | **Wancata, 2008** | Austria | Mothers and the fathers of patients suffering from schizophrenia according to ICD-10, at least 16 years of age, and live in the same household of at least one of their parents or have personal contact with them several times per week | Schizophrenia and other primary psychotic disorders | Cross-sectional study | Caregivers’ Needs Assessment for Schizophrenia |
| **[61]** | **Hayes, 2015** | Australia | Caregivers of an immediate family member with a diagnosis of schizophrenia, each attending one of five community mental health clinics located in suburban Melbourne, Australia | Schizophrenia and other primary psychotic disorders | Cross-sectional study | World Health Organization Quality of Life Questionnaire; Friendship Scale |
| **[62]** | **Herrema, 2017** | U.K. | Family members of autistic adults | Autism spectrum disorder | Cross-sectional study | Penn State Worry Questionnaire; Depression, Anxiety and Stress Scale; Quality of Life Measure |
| **[63]** | **Heru, 2004** | U.S.A. | Caregivers of relatives with mood disorders that were recruited during their relative’s in-patient psychiatric hospitalization between 1997–1999 | Depressive disorders; Bipolar and related disorders | Cross-sectional study | Medical Outcomes Study Short-Form Health Survey |
| **[64]** | **Kaya, 2019** | Turkey | Primary caregivers of schizophrenic patients receiving outpatient treatment at psychiatry polyclinics in hospitals located within Ankara. | Schizophrenia and other primary psychotic disorders | Cross-sectional study | Zarit Burden Interview |
| **[65]** | **Lemoine, 2005** | Canada | Caregivers that offer any kind of non-professional assistance to someone close to them once every two weeks in the last month | Non-specified mental disorders | Cross-sectional study | Experience of Caregiving Inventory |
| **[66]** | **Lerner, 2018** | U.S.A. | Caregivers of a person diagnosed with schizophrenia or schizoaffective disorder, or both, who provided unpaid help in the past 12 months to a relative or friend (or arranged for such help), including assisting with household chores, finances, and personal or medical needs | Schizophrenia and other primary psychotic disorders | Cross-sectional study | Perceived Stress Scale |
| **[67]** | **Ruiz-Robledillo, 2013** | Spain | Parents of relatives with a diagnosis of autism spectrum disorder from an association of relatives of people with autism spectrum disorder in the region of Valencia. | Autism spectrum disorder | Case-control study | Somatic Symptom Scale |
| **[68]** | **Schiffman, 2014** | U.S.A. | Parents who participated in a randomized controlled trial investigating the effectiveness of a Family-to-Family program | Non-specified mental disorders | Cross-sectional study | Experience of Caregiving Inventory; Family Empowerment Scale; Brief Symptom Inventory; Family Assessment Device; Family Experiences Interview Schedule; Knowledge Measure |
| **[69]** | **Tarricone, 2006** | Spain; England | Primary caregiver (i.e., the relative spending the highest number of hours in contact with the patient during the last year) of patients suffering from functional psychotic disorders and were treated in the community mental health care setting | Non-specified mental disorders | Cross-sectional study | Experience of Caregiving Inventory |
| **[70]** | **Wong, 2008** | U.S.A. | Families of clinical high-risk and recent onset psychosis patients that were recruited from the New York State Psychiatric Institute and Yale’s Department of Psychiatry. | Schizophrenia and other primary psychotic disorders | Cross-sectional study | Family Experiences Interview Schedule |
| **[71]** | **Sapouna, 2015** | Greece | Caregivers of patients with a diagnosis of a major mental disorder and attended inpatient, outpatient, or day care specialist mental health services in the previous 5 years at the Psychiatry Clinic of University Hospital of Heraklion, Crete or the Hellenic Center for Mental Health and Research, Branch of Heraklion, Crete | Non-specified mental disorders | Cross-sectional study | Involvement Evaluation Questionnaire |
| **[72]** | **Arciszewska, 2015** | Poland | Spouses of bipolar patients | Bipolar and related disorders | Cross-sectional study | General Health Questionnaire; Involvement Evaluation Questionnaire |
| **[73]** | **Goossens, 2008** | Netherlands | Caregivers of psychiatric outpatients. The patients originated from five psychiatric outpatient clinics in the Netherlands | Bipolar and related disorders | Cross-sectional study | Involvement Evaluation Questionnaire; General Health Questionnaire |
| **[74]** | **Ak, 2012** | Turkey | Relatives of patients who were living with them and served as their primary caregiver. Patients were admitted to an inpatient clinic between July and December 2007 and followed-up from an outpatient clinic and had at least a 4-year history of disease. | Bipolar and related disorders; Schizophrenia and other primary psychotic disorders | Cross-sectional study | Zarit Burden Interview |
| **[75]** | **Angermeyer, 2006** | Germany | Spouses of patients diagnosed with schizophrenia, depression, or anxiety disorder and were in treatment at the inpatient and outpatient services of three psychiatric hospitals or the outpatient facilities of the community mental health services in Leipzig | Schizophrenia and other primary psychotic disorders; Depressive disorders; Anxiety disorders | Cross-sectional study | Maslach Burnout Inventory- Human Services Survey; World Health Organization Quality of Life Questionnaire |
| **[76]** | **Athay, 2012** | U.S.A. | Caregivers of youth receiving mental health treatment | Non-specified mental disorders | Prospective cohort study | Satisfaction with Life Scale |
| **[77]** | **Caqueo-Urizar, 2016** | Chile | Family members of patients with schizophrenia treated by the mental health service in Arica, Chile | Schizophrenia and other primary psychotic disorders | Cross-sectional study | Entrevista de Carga Familiar Objetiva y Subjetiva/Objective and Subjective Family Burden Interview |
| **[78]** | **Chartier-Otis, 2009** | Canada | Partners of civilian individuals that had a primary diagnosis of post-traumatic stress disorder | Trauma- and stressor-related disorders | Cross-sectional study | Medical Outcome Survey-Short Form; Beck Anxiety Inventory; Beck Depression Inventory; Marital Adjustment Test |
| **[79]** | **Mittendorfer-Rutz, 2019** | Sweden | Parents of patients with schizophrenia | Schizophrenia and other primary psychotic disorders | Prospective cohort study | Insurance-Medicine-All Sweden (IMAS) study |
| **[80]** | **Cicek, 2013** | Turkey | First-degree relatives of patients with obsessive compulsive disorder | Obsessive-compulsive and related disorder | Case-control study | Zarit Burden Interview; World Health Organization Quality of Life Questionnaire |
| **[81]** | **Cicek, 2015** | Turkey | Relatives of patients with heroin dependence admitted to a psychiatry outpatient clinic | Substance-related and addictive disorders | Case-control study | Zarit Burden Interview; World Health Organization Quality of Life Questionnaire |
| **[82]** | **Cleary, 2008** | Australia | Caregivers (i.e., partner, parent, relative or friend) of adult inpatients from a mental health service in New South Wales | Non-specified mental disorders | Cross-sectional study | Hospital Anxiety and Depression Scale; Involvement Evaluation Questionnaire |
| **[83]** | **Lawn, 2015** | Australia | Family caregivers of people diagnosed with borderline personality disorder seeking and receiving support from public and private health systems. Caregivers were recruited from 20 clinical mental health and non‐government community organizations | Personality disorders | Cross-sectional study | Self-developed survey by the Private Mental Health Consumer Caregiver Network |
| **[84]** | **Lindenbaum, 2014** | Germany | Insured caregivers providing informal care to frail elderly persons at home | Non-specified mental disorders | Cross-sectional study | Techniker Krankenkasse |
| **[85]** | **Loughland, 2009** | Australia | First-degree relatives of patients with psychosis and listed on the Schizophrenia Research Register | Schizophrenia and other primary psychotic disorders | Cross-sectional study | Perceptions of Prevalence of Aggression Scale; Impact of Event Scale- Revised |
| **[86]** | **Magliano, 2006** | Italy | Primary family caregivers of patients with a DSM-IV diagnosis of schizophrenia | Schizophrenia and other primary psychotic disorders | Case-control study | Social Network Questionnaire; Family Problems Questionnaire |
| **[87]** | **Manor-Binyamini, 2012** | Israel | Parents of children with conduct disorder from special education schools in three school districts in Israel | Personality disorders | Case-control study | Caregiver Strain Index; Sense of Coherence Index |
| **[88]** | **Möller-Leimkühler, 2006** | Germany | Primary family caregivers (i.e., lived with patient or had at least 15 hours of facial contact per week) of inpatients first hospitalized for a schizophrenic or depressive episode from the German Research Networks of Schizophrenia or Depression | Schizophrenia and other primary psychotic disorders; Depressive disorders | Prospective cohort study | Family Burden Questionnaire |
| **[89]** | **Poon, 2018** | Australia | Family caregivers of young adults (14-25 years) with first episode psychosis treated at the Bondi Early Psychosis Program, Southeastern Local Health District in Sydney. | Schizophrenia and other primary psychotic disorders | Cross-sectional study | Kessler Psychological Distress Scale; Australian Type 2 Diabetes Risk Assessment Tool |
| **[90]** | **Shahly, 2013** | Belgium; France; Germany; Israel; Italy; Netherlands; Northern Ireland; Portugal; Spain; United States; Brazil; Bulgaria; Lebanon; Mexico; Romania; Colombia; India; Iraq; Nigeria; People´s Republic of China | First-degree relatives of patients | Non-specified mental disorders | Cross-sectional study | Zarit Burden Interview |
| **[91]** | **Slaunwhite, 2017** | Canada | Caregivers for patients with a long-term health issue, disability or ageing that were non-institutionalized, 15 years or older, and residing in Canada | Non-specified mental disorders | Cross-sectional study | General Social Survey Questionnaire |
| **[92]** | **Weimand, 2010** | Norway | Active members of the Norwegian National Association for Families of Mentally Ill Persons | Non-specified mental disorders | Cross-sectional study | Medical Outcomes Short-Form Health Survey; Burden Assessment Schedule |
| **[93]** | **Storch, 2008** | U.S.A. | Parents/guardians of children and adolescents who were evaluated for treatment of the child’s obsessive-compulsive disorder. | Obsessive- compulsive and related disorders | Cross-sectional study | Parent Experience of Chronic Illness |
| **[94]** | **Lovell, 2014** | England | Parents that cared for at least one child aged 3-19 years with a clinically verified diagnosis of autism | Autism spectrum disorder | Case-control study | Prospective and Retrospective Memory Questionnaire |
| **[95]** | **Lohrer, 2007** | U.S.A. | Siblings (≥ 18 years) of patients with a severe mental illness | Non-specified mental disorders | Cross-sectional study | Self-developed questionnaire by Lohrer et al. [189] |
| **[96]** | **Kronenberg, 2016** | Netherlands | Caregivers of treatment-seeking substance-related and addictive disorders who either had a comorbid diagnosis of ADHD, ASD, or not such comorbid diagnosis | Substance-related and addictive disorders | Cross-sectional study | Level of Expressed Emotion; Involvement Evaluation Questionnaire |
| **[97]** | **Grandón, 2008** | Chile | Primary caregivers (i.e., the member of the family who was most involved with the care recipient) of outpatients with schizophrenia who attended the Psychiatry and Mental Health unit of the Hospital “Las Higueras” | Schizophrenia and other primary psychotic disorders | Cross-sectional study | Entrevista de Carga Familiar Objetiva y Subjetiva/Objective and Subjective Family Burden Interview |
| **[98]** | **Greenberg, 2004** | U.S.A. | Mothers of adults with down syndrome, schizophrenia, and autism | Schizophrenia and other primary psychotic disorders; Autism spectrum disorder | Cross-sectional study | Psychological Wellbeing (PWB) Scale; Center for Epidemiologic Studies-Depression; Self-developed scale by Greenberg et al. [161] |
| **[99]** | **González, 2012** | Spain | Primary caregiver of an outpatient diagnosed with an eating disorder attending the Eating Disorders Outpatient Clinic of the Psychiatric Services at Galdakao-Usansolo Hospital and Ortuella Mental Health Center in Bizkaia, Spain | Eating disorders | Prospective cohort study | Involvement Evaluation Questionnaire |
| **[100]** | **Goodman, 2011** | U.S.A. | Parents of children with borderline personality disorder from the National Education Alliance for Borderline Personality Disorder | Personality disorders | Cross-sectional study | Self-developed questionnaire Goodman et al. [68] |
| **[101]** | **Graap, 2008** | Germany | Primary caregivers (i.e., live with the patient or must have personal contact with the patient at least several times a week) of patients with anorexia nervosa, bulimia nervosa or schizophrenia from the outpatient, day patient, and inpatient services of the Department of Psychosomatic Medicine and Psychotherapy and the Department of Psychiatry and Psychotherapy at the University Hospital of Erlangen | Eating disorders; Schizophrenia and other primary psychotic disorders | Cross-sectional study | Caregivers´ Needs Assessment for Schizophrenia; General Health Questionnaire; Zarit Burden Interview |
| **[102]** | **Graap, 2007** | Germany | Primary caregivers (i.e., the caregiver with the most frequent contact with the patient) of patients that were recruited from the outpatient, day patient and inpatient service of the Department for Psychosomatic Medicine and Psychotherapy of the University of Erlangen | Eating disorders | Cross-sectional study | Caregivers´ Needs Assessment for Schizophrenia; General Health Questionnaire; Zarit Burden Interview |
| **[103]** | **Grootscholten, 2018** | Netherlands; Denmark; United Kingdom | Relatives, friends, partners, or other caregivers of patients | Depressive disorders; Schizophrenia and other primary psychotic disorders; Autism spectrum disorder | Cross-sectional study | Involvement Evaluation Questionnaire |
| **[104]** | **Gupta, 2015** | Germany; France; Italy; Spain; United Kingdom | Adult caregivers of patients | Schizophrenia and other primary psychotic disorders | Case-control study | 2010, 2011, and 2013 EU5 National Health and Wellness Survey; Work Productivity and Impairment Questionnaire |
| **[105]** | **Joling, 2019** | Netherlands | Caregivers from the Netherlands Mental Health Survey and Incidence Study-2, a psychiatric epidemiological cohort study in the Dutch general population aged 18 to 64 years at baseline | Non-specified mental disorders | Prospective cohort study | Composite International Diagnostic Interview |
| **[106]** | **Martín, 2013** | Spain | Family caregivers (i.e., parent, partner, child sibling or other relative) of outpatients diagnosed with, and treated for, an eating disorder in the Eating Disorders Outpatient Clinic of the Psychiatric Services at a hospital and mental health center, both in Bizkaia, Spain. The caregiver must maintain frequent contact with the patient, provide significant financial support to the patient, often be present during the patient’s treatment, is aware of the severity of the patient’s illness, and is the person whom the therapy team is asked to contact in the event of an emergency. | Eating disorders | Prospective cohort study | Medical Outcome Survey-Short Form; Hospital Anxiety and Depression Scale; Anorectic Behaviour Observation Scale; Involvement Evaluation Questionnaire |
| **[107]** | **Raenker, 2013** | England | Caregivers of patients admitted to the National Health Service specialist eating disorder services | Eating disorders | Cross-sectional study | World Health Organization Quality of Life Questionnaire; Eating Disorder Symptom Impact Scale; Care-ED; Depression, Anxiety and Stress Scale |
| **[108]** | **Rogers, 2012** | U.S.A. | Adult caregivers that completed the Church Census | Non-specified mental disorders | Cross-sectional study | Christian Faith Practices Scale; Family Strengths Scale |
| **[109]** | **Caqueo-Urizar, 2011** | Chile | Primary family caregivers (i.e., spent the most time supporting and taking care of the patient) of patients with schizophrenia who attended public mental health centers in Arica | Schizophrenia and other primary psychotic disorders | Cross-sectional study | Zarit Burden Interview; Attitudes Towards Schizophrenia Questionnaire for Relatives |
| **[110]** | **Hanzawa, 2010** | Japan; Korea | Members that attended the general meeting of the Federation of Families of People with Mental Illness in Nagasaki, Seoul, and Daegu | Schizophrenia and other primary psychotic disorders | Cross-sectional study | Zarit Burden Interview |
| **[111]** | **Yıldırım, 2018** | Turkey | Family caregivers who stayed with the inpatient in the psychiatry clinic during treatment at the psychiatry clinic of Ege University Medical Faculty Hospital | Schizophrenia and other primary psychotic disorders; Depressive disorder; Bipolar and related disorders; Substance-related and addictive disorders | Cross-sectional study | State-Trait Anger Scale; Caregiver Burden Inventory |
| **[112]** | **Poon, 2017** | Australia | Caregivers from the Survey of High Impact Psychosis | Schizophrenia and other primary psychotic disorders | Prospective cohort study | Mental Illness Version of the Texas Inventory of Grief; Kessler Psychological Distress Scale; World Health Organization Quality of Life Questionnaire; Friendship Scale |
| **[113]** | **Boydell, 2014** | England | Caregivers (i.e., considered by the patient to be closest to them and who were involved in their lives) of patients suffering from first-episode psychosis in South London, Nottingham, and Bristol between 1997 and 1999 | Schizophrenia and other primary psychotic disorders | Cross-sectional study | General Health Questionnaire; Experience of Caregiving Inventory |
| **[114]** | **Onwumere, 2017** | England | Caregivers of patients under the care of an Early Intervention in Psychosis Service in the Central and Northwest London National Health Service Foundation Trust | Schizophrenia and other primary psychotic disorders | Cross-sectional study | Maslach Burnout Inventory-Human Services Survey |
| **[115]** | **Cummings, 2008** | U.S.A. | Family caregivers of older clients who were diagnosed a severe mental illness. Caregivers had to fulfill the following criteria (1) being a familial caregiver of an older client, 55 years and over, who had received a diagnosis of schizophrenia, schizoaffective disorder, bipolar disorder, or major recurrent depression and (2) having provided instrumental or emotional support to the care recipient within the last month | Non-specified mental disorders | Cross-sectional study | Family Burden Interview Schedule |
| **[116]** | **Van Wijngaarden, 2009** | Netherlands; Denmark; United Kingdom | Caregivers and patients that were recruited from primary studies. | Schizophrenia and other primary psychotic disorders; Depressive disorders | Cross-sectional study | Involvement Evaluation Questionnaire |
| **[117]** | **Shivers, 2017** | U.S.A.; Canada; Republic of Ireland | Family caregivers over the age of 18 of individual with autism spectrum disorder | Autism spectrum disorder | Cross-sectional study | Caregiver Strain Questionnaire |
| **[118]** | **Kokurcan, 2015** | Turkey | Caregivers of patients diagnosed with schizophrenia according to the DSM-IV-TR criteria | Schizophrenia and other primary psychotic disorders | Cross-sectional study | Maslach Burnout Inventory-Human Services Survey |
| **[119]** | **Lauber, 2005** | Switzerland | Members of a Swiss self-help organization for relatives of patients with schizophrenia | Schizophrenia and other primary psychotic disorders | Cross-sectional study | Interview for Measuring the Burden on the Family |
| **[120]** | **Hanzawa, 2013** | Korea | Caregivers from general meetings of the Federation of Families of People with Mental Illness in Seoul and Daegu | Schizophrenia and other primary psychotic disorders | Cross-sectional study | Impact of Event Scale- Revised; Mental Illness and Disorder Understanding Scale; Zarit Burden Interview |
| **[121]** | **Angermeyer, 2006** | Germany | Partners of patients with depression or schizophrenia from four psychiatric hospitals between March 2002 and May 2004. Partners shared the household with the ill person | Depressive disorders; Schizophrenia and other primary psychotic disorders | Cross-sectional study | World Health Organization Quality of Life Questionnaire; Maslach Burnout Inventory-Human Services Survey |
| **[122]** | **Gutiérrez-Maldonado, 2005** | Chile | Primary family caregivers’ patients with schizophrenia who attended Mental Health Outpatient Centers in Arica from June to August 2003 | Schizophrenia and other primary psychotic disorders | Cross-sectional study | Medical Outcomes Study Short-Form Health Survey; Zarit Burden Interview |
| **[123]** | **Aylaz, 2017** | Turkey | Primary family caregiver (i.e., parent, sibling, spouse, child) that provided-home care to a psychiatric patient for at least 6 months | Non-specified mental disorders | Cross-sectional study | Zarit Burden Interview |
| **[124]** | **Barker, 2011** | U.S.A. | Families of adolescents  and adults with an autism spectrum disorder | Autism spectrum disorder | Prospective cohort study | Center for Epidemiologic Studies-Depression; Profile of Mood States |
| **[125]** | **Bowman, 2017** | Australia | Siblings of patients with first episode psychosis that attended the Early Psychosis Prevention and Intervention Centre, Orygen Youth Health in Melbourne | Schizophrenia and other primary psychotic disorders | Cross-sectional study | World Health Organization Quality of Life Questionnaire; Adult Sibling Relationship Questionnaire; Experience of Caregiving Inventory |
| **[126]** | **Butterworth, 2010** | Australia | Primary caregivers that provide more than 5 hours of care per week | Non-specified mental disorders | Cross-sectional study | Goldberg Anxiety and Depressions Scales |
| **[127]** | **Ohara, 2016** | Japan | Primary caregivers of outpatients with anorexia nervosa from the Institute of Women’s Health, Tokyo Women’s Medical University, between August 2012 and March 2014. The primary caregiver was defined as a member of the patient’s family who was providing the most care for the patient, as recognized by both the patient and the caregiver | Eating disorders | Cross-sectional study | General Health Questionnaire; Zarit Burden Interview |
| **[128]** | **Boyer, 2012** | Chile; France | Caregivers of patients using public mental health outpatient services in Arica and Marseille. | Schizophrenia and other primary psychotic disorders | Cross-sectional study | Medical Outcomes Study Short-Form Health Survey |
| **[129]** | **Coomber, 2013** | Australia | Caregivers (i.e., partners, siblings, parents, and friends) of patients diagnosed with eating disorders | Eating disorders | Prospective cohort study | Eating Disorder Symptom Impact Scale |
| **[130]** | **Sruamsiri, 2018** | Japan | Caregivers of schizophrenia patients. Patients were diagnosed for at least 1 year and were currently receiving at least one schizophrenia treatment | Schizophrenia and other primary psychotic disorders | Cross-sectional study | Work Productivity and Impairment Questionnaire |
| **[131]** | **Bravo-Ortiz, 2011** | Spain | Caregivers of outpatients | Schizophrenia and other primary psychotic disorders | Prospective cohort study | Zarit Burden Interview |
| **[132]** | **Labrum, 2018** | U.S.A. | Caregivers of an adult with a serious to moderate mental health issue | Non-specified mental disorders | Cross-sectional study | Self-developed survey by Labrum & Solomon [150] |
| **[133]** | **Mackay, 2011** | Australia | Caregivers of an adult with a mental illness from one of two caregiver support organizations in Queensland and two government mental health services in Brisbane | Non-specified mental disorders | Prospective cohort study | Depression, Anxiety and Stress Scale; Positive and Negative Affect Scale-Expanded Form; Satisfaction with Life Scale (SWLS); Physical Health Rating; Stress Related Growth Scale-Revised |
| **[134]** | **McCrone, 2005** | England | Primary caregivers (i.e., the person who was identified by both patient and the caregiver themselves as the one most involved in a caring role) of patients suffering from a psychotic disorder as diagnosed by their consultant being treated by two community mental health teams in Camberwell and Peckham. A caregiver was defined as someone in at least monthly face-to-face contact in a supportive role toward the patient and was considered to be in such a role by the patient and themselves | Schizophrenia and other primary psychotic disorders; Bipolar and related disorders; Depressive disorders | Cross-sectional study; Case control study | Client Service Receipt Inventory |
| **[135]** | **Ostman, 2005** | Sweden | Caregivers (i.e., parents, spouses, grown-up children, siblings, and non-relatives) of inpatients using psychiatric services with a comprehensive responsibility in two counties in Sweden | Non-specified mental disorders | Prospective cohort study | Family Burden Questionnaire |
| **[136]** | **Page, 2006** | Australia | Inpatient-caregiver dyads for inpatients admitted to one of three psychiatric clinics- Delmont Hospital, Victoria; Perth Clinic, Western Australia; Toowong Private Hospital, Queensland | Non-specified mental disorders | Cross-sectional study | Depression, Anxiety and Stress Scale; Burden Assessment Scale |
| **[137]** | **Sepulveda, 2014** | Spain | Primary caregiver (i.e., the caregiver with the most contact with the patient) of patients diagnosed with an eating disorder. Most of the caregivers were voluntarily recruited from several Spanish Eating Disorders Services-consecutive admissions or outpatient services at two public hospitals, the Eating Disorders Service at the Marques of Valdecilla Hospital and the Child and Adolescent Psychiatric Section at the Nino Jesus University Hospital- and from the Spanish Eating Disorders Caregivers Association. | Eating disorders | Cross-sectional study | Eating Disorder Symptom Impact Scale; General Symptom Index; Level of Expressed Emotion |
| **[138]** | **Smith, 2010** | U.S.A. | Mothers of children clinically diagnosed with autism spectrum disorder that lived at home with their mother | Autism spectrum disorder | Case-control study | Non-Specific Psychological Distress and Positive Emotions Scale |
| **[139]** | **Fujino, 2009** | Japan | Individuals receiving home care nursing services and family caregivers who met the following eligibility criteria: (a) family caregiver is living with a family member with mental illness; (b) both the patient with mental illness and the family member are capable of filling out the questionnaire and engaging in verbal communication; and (c) patient with mental illness is over 20 years old and with a history of admission to a psychiatric hospital or department but without mental retardation, dementia, alcohol addiction, drug addiction, or other organic abnormalities | Non-specified mental disorders | Cross-sectional study | Zarit Burden Interview |
| **[140]** | **Ghosh, 2009** | U.S.A. | Fathers aged 55 years or older of adults with schizophrenia | Schizophrenia and other primary psychotic disorders | Case-control study | Ryff´s Measure of Psychological Wellbeing; Center for Epidemiologic Studies-Depression; Medical Outcome Survey-Short Form; Martial Satisfaction Questionnaire for Older Persons |
| **[141]** | **Gonzalez-Bono, 2011** | Spain | Primary family caregivers from the local Association of Relatives of Mental Illness Patients among voluntary participants | Schizophrenia and other primary psychotic disorders | Case-control study | General Stress Scale |
| **[142]** | **Hastrup, 2011** | Netherlands | Caregivers from Dutch regional support centers for informal caregivers | Non-specified mental disorders | Case-control study | Caregiver Strain Index |
| **[143]** | **Hare, 2004** | England | Family caregivers of individuals clinically diagnosed with autism spectrum disorder that lived with their families | Autism spectrum disorder | Cross-sectional study | General Health Questionnaire; Family Support Scale; Client Service Receipt Inventory; Self-developed questionnaire by Chamba et al. [75] |
| **[144]** | **Ishizaki, 2004** | Japan | Caregivers and their care recipients that consisted of inpatients and outpatients with schizophrenia or depression according to ICD-10F from four public hospitals in Nagasaki from 1999 to 2000. | Schizophrenia and other primary psychotic disorders; Depressive disorders | Case-control study | World Health Organization Quality of Life Questionnaire |
| **[145]** | **Mulligan, 2013** | England | Primary family caregivers (i.e., provided more care to the patient than other members of the family).to patients diagnosed with psychosis and were within 3 years of the first treated episode. Caregivers were required to have a minimum of 10 hours face-to-face contact per week with the patients. | Schizophrenia and other primary psychotic disorders | Cross-sectional study | Texas Inventory of Grief- Early Intervention; Relatives’ Urgent Needs schedule- Early Intervention |
| **[146]** | **Tomlinson, 2014** | England | Relatives of early psychosis patients from three early intervention services in South London. The relatives spent a minimum of 10 hours of weekly contact with the patients | Schizophrenia and other primary psychotic disorders | Cross-sectional study | Hospital Anxiety and Depression Scale; Family Attitudes Scale; Experience of Caregiving Inventory |
| **[147]** | **Byrom, 2019** | U.K. | Students that supported someone experiencing mental health difficulties | Non-specified mental disorders | Cross-sectional study | Experience of Caregiving Inventory; Involvement Evaluation Questionnaire |
| **[148]** | **Ali, 2015** | Sweden | Young caregivers (16-25 years) supporting a person with a mental illness | Non-specified mental disorders | Cross-sectional study | Self-developed questionnaire by Ali et al. [159]; Perceived Stress Scale; COPE Index; General Self-Efficacy Instrument |
| **[149]** | **Cleary, 2006** | Australia | Caregivers (i.e., a parent, partner, relative or friend) that provided support to the patient in the previous three months. Patients originated from the Health Service which comprises inpatient and community public mental health facilities from April-May 2020 | Non-specified mental disorders | Cross-sectional study | Hospital Anxiety and Depression Scale; Involvement Evaluation Questionnaire |
| **[150]** | **Daniels, 2017** | Albania; Bulgaria; Croatia; Turkey | Parents or primary caregivers of children (≤18 years) with a clinical diagnosis of autism spectrum disorder | Autism spectrum disorder | Cross-sectional study | Family Quality of Life Survey; Affiliate Stigma Scale |
| **[151]** | **Pirkis, 2010** | Australia | Caregivers from a stratified multistage probability sample of households | Non-specified mental disorders | Cross-sectional study | Kessler Psychological Distress Scale; Composite International Diagnostic Interview |
| **[152]** | **Poon, 2018** | Australia | Caregivers of patients from individually focused psychiatric services | Schizophrenia and other primary psychotic disorders; Bipolar and related disorders; Depressive disorders | Prospective cohort study | Caregivers’ and Users’ Expectations of Services—Caregiver version |
| **[153]** | **Sin, 2016** | England | Siblings (≥16 years) who were biologically related, step- or half-siblings, or related through adoption that had weekly contact with their sibling that experienced first-episode psychosis | Schizophrenia and other primary psychotic disorders | Cross-sectional study; Case control study | Experience of Caregiving Inventory; Warwick-Edinburgh Mental Wellbeing Scale; Mental Health Knowledge Schedule |
| **[154]** | **Leith, 2018** | U.S.A. | A national sample of adult siblings who reported having a sibling with a serious mental illness | Non-specified mental disorders | Cross-sectional study | Family Satisfaction Scale; Intention to Care Scale; Well Sibling Guilt Questionnaire; Self- and Sibling-Care Measure; Burden Assessment Scale |
| **[155]** | **Bhullar, 2017** | Australia | Parents who were served as caregivers of young people (12-25 years) with mental disorders | Non-specified mental disorders | Cross-sectional study | Burden Assessment Scale |
| **[156]** | **Shivers, 2019** | U.S.A. | Family caregivers (≥18 years) of an individual with autism spectrum disorder | Autism spectrum disorder | Cross-sectional study | Family Needs Questionnaire |
| **[157]** | **Domínguez-Martínez, 2017** | Spain | Relatives of patients with at-risk mental states or first-episode of psychosis | Schizophrenia and other primary psychotic disorders | Case-control study | Symptom Check List Revised; Family Questionnaire ; Illness Perceptions Questionnaire for Schizophrenia Relatives’ version |
| **[158]** | **Durmaz, 2014** | Turkey | Family caregivers of patients with schizophrenia (according to DSM-IV diagnosis criteria) who were either followed-up at home or hospitalized between November 2010- May 2011 | Schizophrenia and other primary psychotic disorders | Cross-sectional study | Zarit Burden Interview |
| **[159]** | **Koyanagi, 2018** | Bangladesh; Burkina Faso; Chad; Comoros; Ethiopia; Ghana; India; Ivory Coast; Kenya; Laos; Malawi; Mali; Mauritania; Myanmar; Nepal; Pakistan; Republic of Congo; Senegal; Vietnam; Zambia; Zimbabwe; Bosnia Herzegovina; Brazil; China; Croatia; Czech Republic; Dominican Republic; Ecuador; Estonia; Georgia; Hungary; Kazakhstan; Latvia; Malaysia Mauritius; Mexico; Morocco; Namibia; Paraguay; Philippines; Russia; Slovakia; South Africa; Sri Lanka; Swaziland; Tunisia; Ukraine; Uruguay; Finland; France; Ireland; Israel; Luxembourg; Norway; Portugal; Spain; Sweden; United Arab Emirates | Caregivers (≥18 years) of patients with non-specified mental disorders | Non-specified mental disorders | Cross-sectional study | Perceived Stress Scale; World Health Organization World Health Survey |
| **[160]** | **Perlick, 2005** | U.S.A. | Primary family caregivers of patients consecutively admitted to a psychiatric inpatient or outpatient service in a medical college-affiliated hospital | Bipolar and related disorders; Schizophrenia and other primary psychotic disorders | Cross-sectional study | Center for Epidemiologic Studies- Depression; Brief Symptom Inventory; Cornell Medical Index; Social Behavior Assessment Schedule; Self-developed questionnaire by Perlick et al. [79] |
| **[161]** | **Sanders, 2013** | U.S.A. | Adults with a sibling clinically diagnosed with a mental disorder | Non-specified mental disorders | Case-control study | Role Behavior Inventory |
| **[162]** | **Viana, 2013** | Colombia; Iraq; Nigeria; People’s Republic of China; Brazil; Bulgaria; Lebanon; Mexico; Romania; Belgium; France; Germany; Israel; Italy; Netherlands; Northern Ireland; Portugal; Spain; U.S.A. | Caregivers of patients with non-specified menta disorders | Non-specified mental disorders | Cross-sectional study | Composite International Diagnostic Interview |
| **[163]** | **Corsentino, 2008** | U.S.A. | Family caregivers of persons with serious mental illness from the National Alliance on Mental Illness | Non-specified mental disorders | Cross-sectional study | Self-developed questionnaire by Corsentino et al. [188] |
| **[164]** | **Csoboth, 2015** | U.S.A. | Caregivers from the 2012 US National Health and Wellness Survey | Schizophrenia and other primary psychotic disorders | Case-control study | Medical Outcome Survey-Short Form; Medical Expenditure Panel Survey; Work Productivity and Impairment Questionnaire; Self-developed scale by Csoboth et al. [153] |
| **[165]** | **De Andrés-García, 2012** | Spain | Parents of patients with autism spectrum disorder that were living at home. Parents served as the primary caregiver (i.e., main provider of first needs) | Autism spectrum disorder | Case-control study | General Health Questionnaire |
| **[166]** | **Wilson, 2015** | Northern Ireland | Caregivers and family members of persons with mental illness who were affiliated with mental health support groups | Non-specified mental disorders | Cross-sectional study | Caregiver Well-Being and Support Questionnaire |
| **[167]** | **Cleary, 2006** | Australia | Caregivers (i.e., a parent, partner, relative or friend) who provided support to a patient in the previous 3 months. Patients (17-65 years) were discharged from acute inpatient services from February-March 2005 | Non-specified mental disorders | Cross-sectional study | Involvement Evaluation Questionnaire |
| **[168]** | **Barnhart, 2020** | U.S.A. | Adult, non-incarcerated caregivers from the 2016 Behavioral Risk Factor Surveillance System | Non-specified mental disorders | Case-control study | 2016 Behavioral Risk Factor Surveillance System |
| **[169]** | **Pearce, 2006** | England | Caregivers (i.e., living with or in 10 hours or more face-to-face contact) belonging to an Asian ethnic group located in Bolton for patients aged 18–65 that were admitted to a psychiatric ward with an ICD-10 diagnosis of schizophrenia, schizoaffective disorder or delusional disorder between April 1996 and December 1999. | Schizophrenia and other primary psychotic disorders | Cross-sectional study | Relatives Cardinal Needs Schedule; General Health Questionnaire |
| **[170]** | **Sono, 2008** | Japan | Respondents from three family associations of persons with mental illness located in Ichikawa and Matsudo. | Non-specified mental disorders | Cross-sectional study | Kreisman’s Family Rejection Scale; Family Life Difficulty Scale; Zarit Burden Interview; Self-developed questionnaire by Sono et al. [72] |
| **[171]** | **Hielscher, 2019** | Australia | Caregivers of patients with a primary mental disorder from Australian state and territory caregiver networks and organizations, including members of Mental Health Caregivers Arafmi Australia and the Mental Illness Fellowship | Non-specified mental disorders | Cross-sectional study | Self-developed questionnaire by Hielscher et al. [51] |
| **[172]** | **Ghosh, 2012** | U.S.A. | Parents of an adult child with a severe mental illness or developmental disability from 2004-2006 | Non-specified mental disorders | Case-control study | Health Utilities Index; Psychological Wellbeing (PWB) Scale; Center for Epidemiologic Studies- Depression; Wisconsin Longitudinal Study (WLS) Survey |
| **[173]** | **Cirici Amell, 2018** | Spain | Caregivers of patients diagnosed with schizophrenia or other schizophrenic spectrum disorders that attended two public mental health centers- the Adult Mental Health Centre outpatient’s center and the inpatient Sub-Acute Psychiatric Unit- between May 2007 and September 2008 | Schizophrenia and other primary psychotic disorders | Cross-sectional study | Zarit Burden Interview |

# References

1. Heru AM, Ryan CE. Burden, reward and family functioning of caregivers for relatives with mood disorders: 1-year follow-up. J Affect Disord. 2004;83(2-3): 221-225. doi: 10.1016/j.jad.2004.04.013.
2. Ozlu A, Yildiz M, Aker T. Burden and burden-related features in caregivers of schizophrenia patients. Düşünen Adam. 2015;28(2): 147-153. doi: 10.5350/DAJPN2015280207.
3. Padierna A, Martín J, Aguirre U, González N, Muñoz P, Quintana JM. Burden of caregiving amongst family caregivers of patients with eating disorders. Soc Psychiatry Psychiatr Epidemiol. 2013;48(1): 151-161. doi: 10.1007/s00127-012-0525-6.
4. Zendjidjian X, Richieri R, Adida M, Limousin S, Gaubert N, Parola N, et al. Quality of life among caregivers of individuals with affective disorders. J Affect Disord. 2012:136(3): 660-664. doi: 10.1016/j.jad.2011.10.011.
5. Pérez JJN, Marqués AC. Family burden, social support and community health in caregivers of people with serious mental disorder. Rev Esc Enferm USP. 2018;52: e03351. doi: 10.1590/S1980-220X2017029403351.
6. Winn S, Perkins S, Walwyn R, Schmidt U, Eisler I, Treasure J, et al. Predictors of mental health problems and negative caregiving experiences in carers of adolescents with bulimia nervosa. Int J Eat Disord. 2007;40(2): 171-178. doi: 10.1002/eat.20347.
7. Flyckt L, Löthman A, Jörgensen L, Rylander A, Koernig T. Burden of informal care giving to patients with psychoses: A descriptive and methodological study. Int J Soc Psychiatry. 2013;59(2): 137-146. doi: 10.1177/0020764011427239.
8. Foldemo A, Gullberg M, Ek AC, Bogren L. Quality of life and burden in parents of outpatients with schizophrenia. Soc Psychiatry Psychiatr Epidemiol. 2005;40(2): 133-138. doi: 10.1007/s00127-005-0853-x.
9. Gonçalves-Pereira M, Xavier M, van Wijngaarden B, Papoila AL, Schene AH, Caldas-de-Almeida JM. Impact of psychosis on Portuguese caregivers: a cross-cultural exploration of burden, distress, positive aspects and clinical-functional correlates. Soc Psychiatry Psychiatr Epidemiol. 2013;48(2): 325-335. doi: 10.1007/s00127-012-0516-7.
10. Hanzawa S, Bae JK, Tanaka H, Tanaka G, Bae YJ, Goto M, et al. Family stigma and care burden of schizophrenia patients: Comparison between Japan and Korea. Asia Pac Psychiatry. 2009;1(3): 120-129. doi: 10.1111/j.1758-5872.2009.00039.x.
11. Khanna R, Madhavan SS, Smith MJ, Patrick JH, Tworek C, Becker-Cottrill B. Assessment of health-related quality of life among primary caregivers of children with autism spectrum disorders. J Autism Dev Disord. 2011;41(9): 1214-1227. doi: 10.1007/s10803-010-1140-6.
12. Magliano L, Fiorillo A, De Rosa C, Malangone C, Maj M, National Mental Health Project Working Group. Family burden in long-term diseases: a comparative study in schizophrenia vs. physical disorders. Soc Sci Med. 2005;61(2): 313-322. doi: 10.1016/j.socscimed.2004.11.064.
13. Mizuno E, Iwasaki M, Sakai I, Kamizawa N. Sense of coherence and quality of life in family caregivers of persons with schizophrenia living in the community. Arch Psychiatr Nurs. 2012;26(4): 295-306. doi: 10.1016/j.apnu.2012.01.003.
14. Möller-Leimkühler AM. Burden of relatives and predictors of burden. Baseline results from the Munich 5-year-follow-up study on relatives of first hospitalized patients with schizophrenia or depression. Eur Arch Psychiatry Clin Neurosci. 2005;225(4): 223-231. doi: 10.1007/s00406-004-0550-x.
15. Molyneux GJ, McCarthy GM, McEniff S, Cryan M, Conroy RM. Prevalence and predictors of carer burden and depression in carers of patients referred to an old age psychiatric service. Int Psychogeriatr. 2008;20(6): 1193-1202.
16. Kingston C, Omwumere J, Keen N, Ruffell T, Kuipers E. Posttraumatic stress symptoms (PTSS) in caregivers of people with psychosis and associations with caregiving experiences. J Trauma Dissociation. 2016;17(3): 307-321. doi: 10.1080/15299732.2015.1089969.
17. Wingrove C, Rickwood D. Parents and carers of young people with mental ill-health: What factors mediate the effect of burden on stress?. Couns Psychol Q. 2017;32(1): 121-134. doi: 10.1080/09515070.2017.1384362.
18. Ranieri V, Madigan K, Roche E, McGuinness D, Bainbridge E, Feeney L, et al. Caregiver burden and distress following the patient's discharge from psychiatric hospital. BJPsych Bull. 2017;41(2): 87-91. doi: 10.1192/pb.bp.115.053074.
19. Magliano L, Orrico A, Fiorillo A, Del Vecchio H, Castiello G, Malangone C, et al. Family burden in bipolar disorders: results from the Italian Mood Disorders Study (IMDS). Epidemiol Psichiatr Soc. 2009;18(2): 137-146.
20. Mitsonis C, Voussoura E, Dimopoulos N, Psarra V, Kararizou E, Latzouraki E, et al. Factors associated with caregiver psychological distress in chronic schizophrenia. Soc Psychiatry Psychiatr Epidemiol. 2012;47(2): 331-337. doi: 10.1007/s00127-010-0325-9.
21. Elmståhl S, Dahlrup B, Ekström H, Nordell E. The association between medical diagnosis and caregiver burden: a cross-sectional study of recipients of informal support and caregivers from the general population study 'Good Aging in Skåne', Sweden. Aging Clin Exp Res. 2018;30(9): 1023-1032. doi: 10.1007/s40520-017-0870-0.
22. Babinski DE, Mazzant JR, Merrill BM, Waschbusch DA, Sibley MH, Gnagy EM, et al. Lifetime caregiver strain among mothers of adolescents and young adults with attention-deficit/hyperactivity disorder. J Fam Psychol. 2020;34(3): 342-352. doi: 10.1037/fam0000609.
23. Bailey RC, Grenyer BFS. Supporting a person with personality disorder: a study of carer burden and well-being. J Pers Disord. 2014;28(6): 796-809. doi: 10.1521/pedi_2014_28_136.
24. Caqueo-Urízar A, Gutiérrez-Maldonado J. Burden of care in families of patients with schizophrenia. Qual Life Res. 2006;15(4): 719-724. doi: 10.1007/s11136-005-4629-2.
25. Caqueo-Urízar A, Gutiérrez-Maldonado J, Ferrer-García M, Darrigrande-Molina P. Burden of care in Aymara caregivers of patients with schizophrenia. Rev Psiquiatr Salud Ment. 2012;5(3): 191-196. doi: 10.1016/j.rpsm.2011.07.001.
26. Scheirs JGM, Bok S. Psychological distress in caretakers or relatives of patients with borderline personality disorder. Int J Soc Psychiatry. 2007;53(3): 195-203. doi: 10.1177/0020764006074554.
27. Blanthorn-Hazell S, Gracia A, Roberts J, Boldeanu A, Judge D. A survey of caregiver burden in those providing informal care for patients with schizophrenia or bipolar disorder with agitation: results from a European study. Ann Gen Psychiatry. 2018;17: 8. doi: 10.1186/s12991-018-0178-2.
28. Van Wijngaarden B, Schene AH, Koeter MWJ. Family caregiving in depression: impact on caregivers' daily life, distress, and help seeking. J Affect Disord. 2004;81(3): 211-222. doi: 10.1016/S0165-0327(03)00168-X.
29. Jansen JE, Haahr UH, Harder S, Trauelsen AM, Lyse HG, Pedersen MB, et al. Caregiver distress in first-episode psychosis: the role of subjective appraisal, over-involvement and symptomatology. Soc Psychiatry Psychiatr Epidemiol. 2015;50(3): 371-378. doi: 10.1007/s00127-014-0935-8.
30. Ostman M. Family burden and participation in care: differences between relatives of patients admitted to psychiatric care for the first time and relatives of re-admitted patients. J Psychiatr Ment Health Nurs. 2004;11(5): 608-613. doi: 10.1111/j.1365-2850.2004.00771.x.
31. Parks M, Anastasiadou D, Sánchez JC, Graell M, Sepulveda AR. Experience of caregiving and coping strategies in caregivers of adolescents with an eating disorder: A comparative study. Psychiatry Res. 2018;260: 241-247. doi: 10.1016/j.psychres.2017.11.064.
32. Perlick DA, Rosenheck RA, Miklowitz DJ, Chessick C, Wolff N, Kaczynski R, et al. Prevalence and correlates of burden among caregivers of patients with bipolar disorder enrolled in the Systematic Treatment Enhancement Program for Bipolar Disorder. Bipolar Disord. 2007;9(3): 262-273. doi: 10.1111/j.1399-5618.2007.00365.x.
33. Kızılırmak B, Küçük L. Care Burden Level and Mental Health Condition of the Families of Individuals With Mental Disorders. Arch Psychiatr Nurs. 2016;30(1): 47-54. doi: 10.1016/j.apnu.2015.10.004.
34. Koutra K, Triliva S, Roumeliotaki T, Stefanakis Z, Basta M, Lionis C, et al. Family functioning in families of first-episode psychosis patients as compared to chronic mentally ill patients and healthy controls. Psychiatry Res. 2014;219(3): 486-496. doi: 10.1016/j.psychres.2014.06.045.
35. Las Hayas C, Padierna JA, Bilbao A, Martín J, Muñoz P, Quintana JM. Eating disorders: predictors of change in the quality of life of caregivers. Psychiatry Res. 2014;215(3): 718-726. doi: 10.1016/j.psychres.2013.12.028.
36. de la Rie S, van Furth EF, De Koning A, Noordenbos G, Donker MCH. The quality of life of family caregivers of eating disorder patients. Eat Disord. 2005;13(4): 345-351. doi: 10.1080/10640260591005236.
37. Sagut P, Duman ZÇ. Comparison of Caregiver Burden in First Episode Versus Chronic Psychosis. Arch Psychiatr Nurs. 2016;30(6): 768-773. doi: 10.1016/j.apnu.2016.07.011.
38. Salaberria K, Polo-Lopez R, Cruz-Saez S, Echeburua E, Berry K. Chronic Stress in Immigrants and Relatives of People with Mental Illness: A Comparative Study. Rev Mex de Psicol. 2015;32(1): 7-15.
39. Rane LJ, Fekadu A, Papadopoulos AS, Wooderson SC, Poon L, Markopoulou K, et al. Psychological and physiological effects of caring for patients with treatment-resistant depression. Psychol Med. 2012;42(9): 1825-1833. doi: 10.1017/S0033291711003035.
40. Reinares M, Vieta E, Colom F, Martínez-Arán A, Torrent C, Comes M, et al. What really matters to bipolar patients' caregivers: sources of family burden. J Affect Disord. 2006;94(1-3): 157-163. doi: 10.1016/j.jad.2006.04.022.
41. Ribé JM, Salamero M, Pérez-Testor C, Mercadal J, Aguilera C, Cleris M. Quality of life in family caregivers of schizophrenia patients in Spain: caregiver characteristics, caregiving burden, family functioning, and social and professional support. Int J Psychiatry Clin Pract. 2018:22(1): 23-33. doi: 10.1080/13651501.2017.1360500.
42. Sücüllüoğlu Dikici D, Eser E, Çökmüş FP, Demet MM. Quality of Life and Associated Risk Factors in Caregivers of Patients with Obsessive Compulsive Disorder. Psychiatr Clin Psychopharmacol. 2019;29(4): 579-586. doi: 10.1080/24750573.2018.1496524 .
43. Nordstroem AL, Talbot D, Bernasconi C, Galani Berardo C, Lalonde J. Burden of illness of people with persistent symptoms of schizophrenia: A multinational cross-sectional study. Int J Soc Psychiatry. 2017;63(2): 139-150. doi: 10.1177/0020764016688040.
44. Schwarte R, Timmesfeld N, Dempfle A, Krei M, Egberts K, Jaite C, et al. Expressed emotions and depressive symptoms in caregivers of adolescents with first‐onset anorexia nervosa—A long‐term investigation over 2.5 years. Eur Eat Disord Rev. 2017;25(1): 44-51. doi: 10.1002/erv.2490.
45. Martín J, Padierna A, van Wijngaarden B, Aguirre U, Anton A, Muñoz P, et al. Caregivers consequences of care among patients with eating disorders, depression or schizophrenia. BMC Psychiatry. 2015;15: 124. doi: 10.1186/s12888-015-0507-9.
46. Martorell A, Gutiérrez-Recacha P, Irazábal M, Marsà F, García M. Family impact in intellectual disability, severe mental health disorders and mental health disorders in ID. A comparison. Res Dev Disabil. 2011;32(6): 2847-2852. doi: 10.1016/j.ridd.2011.05.021.
47. Skundberg-Kletthagen H, Hall-Lord ML, Hedelin B, Wangensteen S. Relatives of Inpatients Suffering from Severe Depression: Their Burden and Encounters with the Psychiatric Health Services. Issues Ment Health Nurs. 2016;37(5): 293-298. doi: 10.3109/01612840.2016.1145309.
48. Lloyd H, Singh P, Merritt R, Shetty A, Yiend J, Singh S, et al. A comparison of levels of burden in Indian and white parents with a son or daughter with schizophrenia. Int J Soc Psychiatry. 2011;57(3): 300-311. doi: 10.1177/0020764009354838.
49. Chen WY, Lukens E. Well Being, Depressive Symptoms, and Burden Among Parent and Sibling Caregivers of Persons With Severe and Persistent Mental Illness. Soc Work Ment Health. 2011;9(6): 397-416. doi: 10.1080/15332985.2011.575712.
50. Coomber K, King RM. Perceptions of carer burden: differences between individuals with an eating disorder and their carer. Eat Disord. 2013;21(1): 26-36. doi: 10.1080/10640266.2013.741966.
51. Cummings SM, Kropf NP. Predictors of depression among caregivers of older adults with severe mental illness. J Gerontol Soc Work. 2015;58(3): 253-271. doi: 10.1080/01634372.2014.978927.
52. Ruiz-Robledillo N, Romero-Martínez A, Moya-Albiol L. Blunted Cortisol Awakening Response and Poor Self-Perceived Health in Informal Caregivers of People with Eating Disorders. Eur Eat Disord Rev. 2016;24(5): 383-390. doi: 10.1002/erv.2455.
53. Day NJS, Bourke ME, Townsend ML, Grenyer BFS. Pathological Narcissism: A Study of Burden on Partners and Family. J Pers Disord. 2020;34(6): 799-813. doi: 10.1521/pedi_2019_33_413.
54. Depestele L, Lemmens GMD, Dierckx E, Baetens I, Schoevaerts K, Claes L. The Role of Non-suicidal Self-Injury and Binge-Eating/Purging Behaviours in the Caregiving Experience Among Mothers and Fathers of Adolescents with Eating Disorders. Eur Eat Disord Rev. 2016;24(3): 257-260. doi: 10.1002/erv.2428.
55. Dols A, Thesing C, Wouters M, Theunissen J, Sonnenberg C, Comijs H, et al. Burden on caregivers of older patients with bipolar disorder. Aging Ment Health. 2018;22(5): 686-691. doi: 10.1080/13607863.2017.1297360.
56. Kirtley J, Chiocchi J, Cole J, Sampson M. Stigma, Emotion Appraisal, and the Family Environment as Predictors of Carer Burden for Relatives of Individuals Who Meet the Diagnostic Criteria for Borderline Personality Disorder. J Pers Disord. 2019;33(4): 497-514. doi: 10.1521/pedi_2018_32_355.
57. Linacre S, Heywood-Everett S, Sharma V, Hill AJ. Comparing carer wellbeing: implications for eating disorders. Ment Health Rev J. 2015;20(2): 105-118. doi: 10.1108/MHRJ-12-2014-0046.
58. Velligan DI, Brain C, Bouérat Duvold L, Agid O. Caregiver Burdens Associated With Treatment-Resistant Schizophrenia: A Quantitative Caregiver Survey of Experiences, Attitudes, and Perceptions. Front Psychiatry. 2019;10: 584. doi: 10.3389/fpsyt.2019.00584.
59. Yu Y, McGrew JH, Rand KL, Mosher CE. Using a model of family adaptation to examine outcomes of caregivers of individuals with autism spectrum disorder transitioning into adulthood. Res Autism Spectr Disord. 2018 Oct 1;54: 37-50. doi: 10.1016/j.rasd.2018.06.007.
60. Wancata J, Freidl M, Krautgartner M, Friedrich F, Matschnig T, Unger A, et al. Gender aspects of parents’ needs of schizophrenia patients. Soc Psychiatry Psychiatr Epidemiol. 2008;43(12): 968-974. doi: 10.1007/s00127-008-0391-4.
61. Hayes L, Hawthorne G, Farhall J, O’Hanlon B, Harvey C. Quality of Life and Social Isolation Among Caregivers of Adults with Schizophrenia: Policy and Outcomes. Community Ment Health J. 2015;51(5): 591-597. doi: 10.1007/s10597-015-9848-6.
62. Herrema R, Garland D, Osborne M, Freeston M, Honey E, Rodgers J. Mental Wellbeing of Family Members of Autistic Adults. J Autism Dev Disord. 2017;47(11): 3589-3599. doi: 10.1007/s10803-017-3269-z.
63. Heru AM, Ryan CE, Vlastos K. Quality of life and family functioning in caregivers of relatives with mood disorders. Psychiatr Rehabil J. 2004;28(1): 67-71. doi: 10.2975/28.2004.67.71.
64. Kaya Y, Öz F. Global social functioning of patients with schizophrenia and care burden of caregiving relatives. J Psychiatric Nurs. 2019;10(1): 28-38. doi: 10.14744/phd.2018.43815.
65. Lemoine O, Lavoie S, Poulin C, Poirier LR, Fournier L. Being the caregiver of a person with a mental health problem. Can J Commun Ment Health. 2009;24(2): 127-143. doi: 10.7870/cjcmh-2005-001.
66. Lerner D, Chang H, Rogers WH, Benson C, Lyson MC, Dixon LB. Psychological distress among caregivers of individuals with a diagnosis of schizophrenia or schizoaffective disorder. Psychiatr Serv. 2018;69(2): 169-178. doi: 10.1176/appi.ps.201600422.
67. Ruiz-Robledillo N, Moya-Albiol L. Self-reported health and cortisol awakening response in parents of people with asperger syndrome: the role of trait anger and anxiety, coping and burden. Psychol Health. 2013;28(11): 1246-1264. doi: 10.1080/08870446.2013.800517.
68. Schiffman J, Kline E, Reeves G, Jones A, Medoff D, Lucksted A, et al. Differences Between Parents of Young Versus Adult Children Seeking to Participate in Family-to-Family Psychoeducation. Psychiatr Serv. 2014;65(2): 247-250. doi: 10.1176/appi.ps.201300045.
69. Tarricone I, Leese M, Szmukler GI, Bassi M, Berardi D. The experience of carers of patients with severe mental illness: a comparison between London and Bologna. Eur Psychiatry. 2006;21(2): 93-101. doi: 10.1016/j.eurpsy.2005.09.012.
70. Wong C, Davidson L, McGlashan T, Gerson R, Malaspina D, Corcoran C. Comparable family burden in families of clinical high‐risk and recent‐onset psychosis patients. Early Interv Psychiatry. 2008;2(4): 256-261. doi: 10.1111/j.1751-7893.2008.00086.x.
71. Sapouna V, Dafermos V, Vivilaki V, Chatziarsenis M, Aivaliotis IL, Bitsios P, Lionis C. Exploring the Burden of the Family Caregivers of Patients with Major Mental Disorders in Greece. Rev Clin Pharmacol Pharmacokinet Int Ed. 2015;29(1): 25-35.
72. Arciszewska AI, Siwek M, Dudek D. Caregiving burden and psychological distress among spouses of bipolar patients- comparative analysis of subtype I and II. Psychiatr Pol. 2015;49(6): 1289-1302. doi: 10.12740/PP/OnlineFirst/32177.
73. Goossens PJ, van Wijngaarden B, Knoppert-van der Klein EAM, van Achterberg T. Family caregiving in bipolar disorder: caregiver consequences, caregiver coping styles, and caregiver distress. Int J Soc Psychiatry. 2008;54(4): 303-316. doi: 10.1177/0020764008090284.
74. Ak M, Yavuz F, Lapsekili N, Türkçapar MH. Evaluation of burden in a group of patients with chronic psychiatric disorders and their caregivers. Düşünen Adam. 2012;25(4): 330-337.
75. Angermeyer MC, Kilian R, Wilms HU, Wittmund B. Quality of life of spouses of mentally ill people. Int J Soc Psychiatry. 2006;52(3): 278-285. doi: 10.1177/0020764006067186.
76. Athay MM. Caregiver life satisfaction: Relationship to youth symptom severity through treatment. J Clin Child Adolesc Psychol. 2012;41(4): 433-444. doi: 10.1080/15374416.2012.684273.
77. Caqueo-Urízar A, Urzúa A, Jamett PR, Irarrazaval M. Objective and subjective burden in relatives of patients with schizophrenia and its influence on care relationships in Chile. Psychiatry Res. 2016;237: 361-365. doi: 10.1016/j.psychres.2016.01.013.
78. Chartier-Otis M, Guay S, Merchand A. Psychological and relationship distress among partners of civilian PTSD patients. J Nerv Ment Disor. 2009;197(7): 543-546. doi: 10.1097/NMD.0b013e3181aac807.
79. Mittendorfer-Rutz E, Rahman S, Tanskanen A, Majak M, Mehtälä J, Hoti F, et al. Burden for Parents of Patients With Schizophrenia-A Nationwide Comparative Study of Parents of Offspring With Rheumatoid Arthritis, Multiple Sclerosis, Epilepsy, and Healthy Controls. Schizophr Bull. 2019;45(4): 794-803. doi: 10.1093/schbul/sby130.
80. Cicek E, Cicek IE, Kayhan F, Uguz F, Kaya N. Quality of life, family burden and associated factors in relatives with obsessive–compulsive disorder. Gen Hosp Psychiatry. 2013;35(3): 253-258. doi: 10.1016/j.genhosppsych.2013.01.004.
81. Cicek E, Demirel B, Ozturk HI, Kayhan F, Cicek IE, Eren I. Burden of care and quality of life in relatives of opioid dependent male subjects. Psychiatr Danub. 2015;27(3): 273-277.
82. Cleary M, Hunt GE, Matheson S, Walter G. The association between substance use and the needs of patients with psychiatric disorder, levels of anxiety, and caregiving burden. Arch Psychiatr Nurs. 2008;22(6): 375-385. doi: 10.1016/j.apnu.2008.02.001.
83. Lawn S, McMahon J. Experiences of family carers of people diagnosed with borderline personality disorder. J Psychiatr Ment Health Nurs. 2015;22(4): 234-243. doi: 10.1111/jpm.12193.
84. Lindenbaum K, Stroka MA, Linder R. Informal caregiving for elderly people with mental illnesses and the mental health of the informal caregivers. J Ment Health Policy Econ. 2014;17(3): 99-105.
85. Loughland CM, Lawrence G, Allen J, Hunter M, Lewin TJ, Oud NE, et al. Aggression and trauma experiences among carer-relatives of people with psychosis. Soc Psychiatry Psychiatr Epidemiol. 2009;44(12): 1031-1040. doi: 10.1007/s00127-009-0025-5.
86. Magliano L, Fiorillo A, Rosa C, Maj M, National Mental Health Project Working Group. Family burden and social network in schizophrenia vs. physical diseases: preliminary results from an Italian national study. Acta Psychiatr Scand Suppl. 2006;429: 60-63. doi: 10.1111/j.1600-0447.2005.00719.x.
87. Manor-Binyamini I. Parenting children with conduct disorder in Israel: caregiver burden and the sense of coherence. Community Ment Health J. 2012;48(6): 781-785. doi: 10.1007/s10597-011-9474-x.
88. Möller-Leimkühler AM. Multivariate prediction of relatives' stress outcome one year after first hospitalization of schizophrenic and depressed patients. Eur Arch Psychiatry Clin Neurosci. 2006;256(2): 122-130. doi: 10.1007/s00406-005-0619-1.
89. Poon AW, Curtis J, Ward P, Loneragan C, Lappin J. Physical and psychological health of carers of young people with first episode psychosis. Australas Psychiatry. 2018;26(2): 184-188. doi: 10.1177/1039856217748250.
90. Shahly V, Chatterji S, Gruber MJ, Al-Hamzawi A, Alonso J, Andrade LH, et al. Cross-national differences in the prevalence and correlates of burden among older family caregivers in the World Health Organization World Mental Health (WMH) Surveys. Psychol Med. 2013;43(4): 865-879. doi: 10.1017/S0033291712001468.
91. Slaunwhite AK, Ronis ST, Sun Y, Peters PA. The emotional health and well‐being of Canadians who care for persons with mental health or addictions problems. Health Soc Care Community. 2017;25(3): 840-847. doi: 10.1111/hsc.12366.
92. Weimand BM, Hedelin B, Sällström C, Hall-Lord ML. Burden and health in relatives of persons with severe mental illness: a Norwegian cross-sectional study. Issues Ment Health Nurs. 2010;31(12): 804-815. doi: 10.3109/01612840.2010.520819.
93. Storch EA, Lehmkuhl H, Pence SL, Geffken GR, Ricketts E, Storch JF, et al. Parental experiences of having a child with obsessive-compulsive disorder: Associations with clinical characteristics and caregiver adjustment. J Child Family Stud. 2009;18(3): 249-258.
94. Lovell B, Elliot H, Sung Liu CC, Wetherell MA. Memory failures for everyday tasks in caregivers of children with autism. Res Dev Disabil. 2014;35(11): 3057-3061. doi: 10.1016/j.ridd.2014.07.019.
95. Lohrer SP, Lukens EP, Thorning H. Economic expenditures associated with instrumental caregiving roles of adult siblings of persons with severe mental illness. Community Ment Health J. 2007;43(2): 129-151. doi: 10.1007/s10597-005-9026-3.
96. Kronenberg LM, Goossens PJ, van Busschbach JT, van Achterberg T, van den Brink W. Burden and expressed emotion of caregivers in cases of adult substance use disorder with and without attention deficit/hyperactivity disorder or autism spectrum disorder. Int J Ment Health Addict. 2016;14(1): 49-63. doi: 10.1007/s11469-015-9567-9.
97. Grandón P, Jenaro C, Lemos S. Primary caregivers of schizophrenia outpatients: Burden and predictor variables. Psychiatry Res. 2008;158(3): 335-343. doi: 10.1016/j.psychres.2006.12.013.
98. Greenberg JS, Seltzer MM, Krauss MW, Chou RJA, Hong J. The effect of quality of the relationship between mothers and adult children with schizophrenia, autism, or down syndrome on maternal well-being: the mediating role of optimism. Am J Orthopsychiatry. 2004;74(1): 14-25. doi: 10.1037/0002-9432.74.1.14.
99. González N, Padierna A, Martín J, Aguirre U, Quintana JM. Predictors of change in perceived burden among caregivers of patients with eating disorders. Journal Affect Disord. 2012;139(3): 273-282. doi: 10.1016/j.jad.2012.02.013.
100. Goodman M, Patil U, Triebwasser J, Hoffman P, Weinstein ZA, New A. Parental burden associated with borderline personality disorder in female offspring. J Pers Disord. 2011;25(1): 59-74. doi: 10.1521/pedi.2011.25.1.59.
101. Graap H, Bleich S, Herbst F, Scherzinger C, Trostmann Y, Wancata J, et al. The needs of carers: a comparison between eating disorders and schizophrenia. Social Psychiatry Psychiatr Epidemiol. 2008;43(10): 800-807. doi: 10.1007/s00127-008-0364-7.
102. Graap H, Bleich S, Herbst F, Trostmann Y, Wancata J, de Zwaan M. The needs of carers of patients with anorexia and bulimia nervosa. Eur Eat Disord Rev. 2008;16(1): 21-29. doi: 10.1002/erv.804.
103. Grootscholten IAC, van Wijngaarden B, Kan CC. High functioning autism spectrum disorders in adults: Consequences for primary caregivers compared to schizophrenia and depression. J Autism Dev Disord. 2018;48(6): 1920-1931. doi: 10.1007/s10803-017-3445-1.
104. Gupta S, Isherwood G, Jones K, van Impe K. Productivity loss and resource utilization, and associated indirect and direct costs in individuals providing care for adults with schizophrenia in the EU5. Clinicoecon Outcomes Res. 2015;7: 593-602. doi: 10.2147/CEOR.S94334.
105. Joling KJ, ten Have M, de Graaf R, O’Dwyer ST. Risk factors for suicidal thoughts in informal caregivers: results from the population-based Netherlands mental health survey and incidence Study-2 (NEMESIS-2). BMC Psychiatry. 2019;19(1): 320. doi: 10.1186/s12888-019-2317-y.
106. Martín J, Padierna A, Aguirre U, González N, Muñoz P, Quintana JM. Predictors of quality of life and caregiver burden among maternal and paternal caregivers of patients with eating disorders. Psychiatry Res. 2013;210(3): 1107-1115. doi: 10.1016/j.psychres.2013.07.039.
107. Raenker S, Hibbs R, Goddard E, Naumann U, Arcelus J, Ayton A, et al. Caregiving and coping in carers of people with anorexia nervosa admitted for intensive hospital care. Int J Eat Disord. 2013;46(4): 346-354. doi: 10.1002/eat.22068.
108. Rogers EB, Stanford M, Garland DR. The effects of mental illness on families within faith communities. Ment Health Relig Cult. 2012;15(3): 301-13. doi: 10.1080/13674676.2011.573474.
109. Caqueo-Urízar A, Gutiérrez-Maldonado J, Ferrer-García M, Peñaloza-Salazar C, Richards-Araya D, Cuadra-Peralta A. Attitudes and burden in relatives of patients with schizophrenia in a middle income country. BMC Fam Pract. 2011;12: 101.
110. Hanzawa S, Bae JK, Tanaka H, Bae YJ, Tanaka G, Inadomi H, et al. Caregiver burden and coping strategies for patients with schizophrenia: Comparison between Japan and Korea. Psychiatry Clin Neurosci. 2010;64(4): 377-386. doi: 10.1111/j.1440-1819.2010.02104.x.
111. Yıldırım S, Akyüz Ö, Engin E, Gültekin K. The relationship between psychiatric patients’ caregiver burden and anger expression styles. J Clin Nurs. 2018;27(3-4): 725-731. doi: 10.1111/jocn.14060.
112. Poon AWC, Harvey C, Mackinnon A, Joubert L. A longitudinal population-based study of carers of people with psychosis. Epidemiol Psychiatr Sci. 2017;26(3): 265-275. doi: 10.1017/S2045796015001195.
113. Boydell J, Onwumere J, Dutta R, Bhavsar V, Hill N, Morgan C, et al. Caregiving in first‐episode psychosis: social characteristics associated with perceived ‘burden’and associations with compulsory treatment. Early Interv Psychiatry. 2014;8(2): 122-129. doi: 10.1111/eip.12041.
114. Onwumere J, Lotey G, Schulz J, James G, Afsharzadegan R, Harvey R, et al. Burnout in early course psychosis caregivers: the role of illness beliefs and coping styles. Early Interv Psychiatry. 2017;11(3): 237-243. doi: 10.1111/eip.12227.
115. Cummings SM, MacNeil G. Caregivers of Older Clients with Severe Mental Illness: Perceptions of Burdens and Rewards. Fam Soc. 2018;89(1): 51-59. doi: 10.1606/1044-3894.3709.
116. van Wijngaarden B, Koeter M, Knapp M, Tansella M, Thornicroft G, Vázquez-Barquero JL, et al. Caring for people with depression or with schizophrenia: are the consequences different?. Psychiatry Res. 2009;169(1): 62-69. doi: 10.1016/j.psychres.2008.06.013.
117. Shivers CM, Krizova K, Lee GK. Types of strain among family members of individuals with autism spectrum disorder across the lifespan. Res Dev Disabil. 2017;68: 42-51. doi: 10.1016/j.ridd.2017.07.003.
118. Kokurcan A, Özpolat AGY, Göğüş AK. Burnout in caregivers of patients with schizophrenia. Turk J Med Sci. 2015;45(3): 678-685. doi: 10.3906/sag-1403-98.
119. Lauber C, Keller C, Eichenberger A, Rössler W. Family burden during exacerbation of schizophrenia: quantification and determinants of additional costs. Int J Soc Psychiatry. 2005;51(3): 259-264. doi: 10.1177/0020764005057376.
120. Hanzawa S, Bae JK, Bae YJ, Chae MH, Tanaka H, Nakane H, et al. Psychological impact on caregivers traumatized by the violent behavior of a family member with schizophrenia. Asian J Psychiatr. 2013;6(1): 46-51. doi: 10.1016/j.ajp.2012.08.009.
121. Angermeyer MC, Bull N, Bernert S, Dietrich S, Kopf A. Burnout of caregivers: a comparison between partners of psychiatric patients and nurses. Arch Psychiatr Nurs. 2006;20(4): 158-165. doi: 10.1016/j.apnu.2005.12.004.
122. Gutiérrez-Maldonado J, Caqueo-Urízar A, Kavanagh DJ. Burden of care and general health in families of patients with schizophrenia. Soc Psychiatry Psychiatr Epidemiol. 2005;40(11): 899-904. doi: 10.1007/s00127-005-0963-5.
123. Aylaz R, Yıldız E. The care burden and coping levels of chronic psychiatric patients’ caregivers. Perspect Psychiatr Care. 2018;54(2): 230-241. doi: 10.1111/ppc.12228.
124. Barker ET, Hartley SL, Seltzer MM, Floyd FJ, Greenberg JS, Orsmond GI. Trajectories of emotional well-being in mothers of adolescents and adults with autism. Dev Psychol. 2011;47(2): 551-561. doi: 10.1037/a0021268.
125. Bowman S, Alvarez-Jimenez M, Wade D, Howie L, McGorry P. The positive and negative experiences of caregiving for siblings of young people with first episode psychosis. Front Psychol. 2017;8: 730. doi: 10.3389/fpsyg.2017.00730.
126. Butterworth P, Pymont C, Rodgers B, Windsor TD, Anstey KJ. Factors that explain the poorer mental health of caregivers: Results from a community survey of older Australians. Aust N Z J Psychiatry. 2010;44(7): 616-624. doi: 10.3109/00048671003620202.
127. Ohara C, Komaki G, Yamagata Z, Hotta M, Kamo T, Ando T. Factors associated with caregiving burden and mental health conditions in caregivers of patients with anorexia nervosa in Japan. BioPsychoSocial Med. 2016;10: 21. doi: 10.1186/s13030-016-0073-5.
128. Boyer L, Caqueo-Urízar A, Richieri R, Lancon C, Gutiérrez-Maldonado J, Auquier P. Quality of life among caregivers of patients with schizophrenia: a cross-cultural comparison of Chilean and French families. BMC Fam Pract. 2012;13: 42. doi: 10.1186/1471-2296-13-42.
129. Coomber K, King RM. A longitudinal examination of burden and psychological distress in carers of people with an eating disorder. Soc Psychiatry Psychiatr Epidemiol. 2013;48(1): 163-171. doi: 10.1007/s00127-012-0524-7.
130. Sruamsiri R, Mori Y, Mahlich J. Productivity loss of caregivers of schizophrenia patients: a cross-sectional survey in Japan. J Ment Health. 2018;27(6): 583-587. doi: 10.1080/09638237.2018.1466048.
131. Bravo-Ortiz MF, Gutiérrez-Casares JR, Rodríguez-Morales A, García MA, Hidalgo-Borrajo R. Influence of type of treatment on the well-being of Spanish patients with schizophrenia and their caregivers. Int J Psychiatry Clin Pract. 2011;15(4): 286-295. doi: 10.3109/13651501.2011.608469.
132. Labrum T, Solomon P. Safety Fears Held by Caregivers about Relatives with Psychiatric Disorders. Health Soc Work. 2018;43(3): 165-174. doi: 10.1093/hsw/hly013.
133. Mackay C, Pakenham KI. Identification of stress and coping risk and protective factors associated with changes in adjustment to caring for an adult with mental illness. J Clin Psychol. 2011;67(10): 1064-1079. doi: 10.1002/jclp.20829.
134. McCrone P, Szmukler G, Kuipers E. Service use and cost associated with caring for people with serious mental illness. J Ment Health. 2005;14(1): 37-47. doi: 10.1080/09638230500048081.
135. Ostman M, Wallsten T, Kjellin L. Family burden and relatives' participation in psychiatric care: are the patient's diagnosis and the relation to the patient of importance?. Int J Soc Psychiatry. 2005;51(4): 291-301. doi: 10.1177/0020764005057395.
136. Page A, Hooke G, O’Brien N, de Felice N. Assessment of distress and burden in Australian private psychiatric inpatients. Australas Psychiatry. 2006;14(3): 285-290. doi: 10.1080/j.1440-1665.2006.02293.x.
137. Sepulveda AR, Anastasiadou D, Pellegrin Y, Andrés P, Graell M, Carrobles JA, et al. Impact of caregiving experience on mental health among caregivers: a comparison of eating disorder patients with purging and non-purging behaviors. Eat Weight Disord. 2014;19(1): 31-39. doi: 10.1007/s40519-013-0064-5.
138. Smith LE, Hong J, Seltzer MM, Greenberg JS, Almeida DM, Bishop SL. Daily experiences among mothers of adolescents and adults with autism spectrum disorder. J Autism Dev Disord. 2010;40(2): 167-178. doi: 10.1007/s10803-009-0844-y.
139. Fujino N, Okamura H. Factors affecting the sense of burden felt by family members caring for patients with mental illness. Arch Psychiatr Nurs. 2009;23(2): 128-137. doi: 10.1016/j.apnu.2008.05.006.
140. Ghosh S, Greenberg J. Aging fathers of adult children with schizophrenia: the toll of caregiving on their mental and physical health. Psychiatr Serv. 2009;60(7): 982-984. doi: 10.1176/ps.2009.60.7.982.
141. Gonzalez-Bono E, De Andres-Garcia S, Moya-Albiol L. The cortisol awakening response in caregivers of schizophrenic offspring shows sensitivity to patient status. Anxiety Stress Coping. 2011;24(1): 107-120. doi: 10.1080/10615806.2010.481792.
142. Hastrup LH, van den Berg B, Gyrd-Hansen D. Do informal caregivers in mental illness feel more burdened? A comparative study of mental versus somatic illnesses. Scand J Public Health. 2011;39(6): 598-607. doi: 10.1177/1403494811414247.
143. Hare DJ, Pratt C, Burton M, Bromley J, Emerson E. The health and social care needs of family carers supporting adults with autistic spectrum disorders. Autism. 2004;8(4): 425-444. doi: 10.1177/1362361304047225.
144. Ishizaki Y, Nakane Y. A survey of patients with mental disorder and their caregivers using the World Health Organization quality of life instrument. Acta Med Nagasaki. 2004;49(4): 143-147. doi: 10.11343/amn.49.143.
145. Mulligan J, Sellwood W, Reid GS, Riddell S, Andy N. Informal caregivers in early psychosis: evaluation of need for psychosocial intervention and unresolved grief. Early Interv Psychiatry. 2013;7(3): 291-299. doi: 10.1111/j.1751-7893.2012.00369.x.
146. Tomlinson E, Onwumere J, Kuipers E. Distress and negative experiences of the caregiving relationship in early psychosis: does social cognition play a role?. Early Interv Psychiatry. 2014;8(3): 253-260. doi: 10.1111/eip.12040.
147. Byrom NC. Supporting a friend, housemate or partner with mental health difficulties: The student experience. Early Interv Psychiatry. 2019;13(2): 202-207. doi: 10.1111/eip.12462.
148. Ali L, Krevers B, Skärsäter I. Caring Situation, Health, Self-efficacy, and Stress in Young Informal Carers of Family and Friends with Mental Illness in Sweden. Issues Ment Health Nurs. 2015;36(6): 407-415. doi: 10.3109/01612840.2014.1002644.
149. Cleary M, Freeman A, Hunt GE, Walter G. Patient and carer perceptions of need and associations with care-giving burden in an integrated adult mental health service. Soc Psychiatry Psychiatr Epidemiol. 2006;41(3): 208-214. doi: 10.1007/s00127-005-0017-z.
150. Daniels AM, Como A, Hergüner S, Kostadinova K, Stosic J, Shih A. Autism in Southeast Europe: A survey of caregivers of children with autism spectrum disorders. J Autism Dev Disord. 2017;47(8): 2314-2325. doi: 10.1007/s10803-017-3145-x.
151. Pirkis J, Burgess P, Hardy J, Harris M, Slade T, Johnston A. Who cares? A profile of people who care for relatives with a mental disorder. Aust N Z J Psychiatry. 2010;44(10): 929-937. doi: 10.3109/00048674.2010.493858.
152. Poon AWC, Joubert L, Harvey C. Perceived needs of carers of people with psychosis: An Australian longitudinal population‐based study of caregivers of people with psychotic disorders. Health Soc Care Community. 2018;26(3): 412-422. doi: 10.1111/hsc.12530.
153. Sin J, Murrells T, Spain D, Norman I, Henderson C. Wellbeing, mental health knowledge and caregiving experiences of siblings of people with psychosis, compared to their peers and parents: an exploratory study. Soc Psychiatry Psychiatr Epidemiol. 2016;51(9): 1247-1255. doi: 10.1007/s00127-016-1222-7.
154. Leith JE, Jewell TC, Stein CH. Caregiving Attitudes, Personal Loss, and Stress-Related Growth Among Siblings of Adults with Mental Illness. J Child Fam Stud. 2018;27: 1193-1206.
155. Bhullar N, Rickwood D, Carter T, Haridas S. Taking care of teenagers, taking care of me: Profiling parental caregiving burden and activity restriction in a sample of Australian parents. Int J Ment Health Nurs. 2017;26(6): 593-601. doi: 10.1111/inm.12285.
156. Shivers CM, Sonnier‐Netto L, Lee GK. Needs and experiences of family caregivers of individuals with autism spectrum disorders across the lifespan. J Policy Pract Intellect Disabil. 2019;16(1): 21-29. doi: 10.1111/jppi.12272.
157. Domínguez-Martínez T, Medina-Pradas C, Kwapil TR, Barrantes-Vidal N. Relatives' expressed emotion, distress and attributions in clinical high-risk and recent onset of psychosis. Psychiatry Res. 2017;247: 323-329. doi: 10.1016/j.psychres.2016.11.048.
158. Durmaz H, Okanlı A. Investigation of the effect of self-efficacy levels of caregiver family members of the individuals with schizophrenia on burden of care. Arch Psychiatr Nurs. 2014;28(4): 290-294. doi: 10.1016/j.apnu.2014.04.004.
159. Koyanagi A, DeVylder JE, Stubbs B, Carvalho AF, Veronese N, Haro JM, et al. Depression, sleep problems, and perceived stress among informal caregivers in 58 low-, middle-, and high-income countries: A cross-sectional analysis of community-based surveys. J Psychiatr Res. 2018;96: 115-123. doi: 10.1016/j.jpsychires.2017.10.001.
160. Perlick DA, Hohenstein JM, Clarkin JF, Kaczynski R, Rosenheck RA. Use of mental health and primary care services by caregivers of patients with bipolar disorder: a preliminary study. Bipolar Disord. 2005;7(2): 126-135. doi: 10.1111/j.1399-5618.2004.00172.x.
161. Sanders A, Szymanski K, Fiori K. The family roles of siblings of people diagnosed with a mental disorder: Heroes and lost children. Int J Psychol. 2014;49(4): 257-262. doi: 10.1002/ijop.12020.
162. Viana MC, Gruber MJ, Shahly V, Alhamzawi A, Alonso J, Andrade LH, et al. Family burden related to mental and physical disorders in the world: results from the WHO World Mental Health (WMH) surveys. Braz J Psychiatry. 2013;35(2): 115-125. doi: 10.1590/1516-4446-2012-0919.
163. Corsentino EA, Molinari V, Gum AM, Roscoe LA, Mills WL. Family Caregivers' Future Planning for Younger and Older Adults With Serious Mental Illness (SMI). J Appl Gerontol. 2008;27(4): 466-485. doi: 10.1177/0733464808315290.
164. Csoboth C, Witt EA, Villa KF, O’Gorman C. The humanistic and economic burden of providing care for a patient with schizophrenia. Int J Soc Psychiatry. 2015;61(8): 754-761. doi: 10.1177/0020764015577844.
165. De Andrés-García S, Moya-Albiol L, González-Bono E. Salivary cortisol and immunoglobulin A: Responses to stress as predictors of health complaints reported by caregivers of offspring with autistic spectrum disorder. Horm Behav. 2012;62(4): 464-474. doi: 10.1016/j.yhbeh.2012.08.003.
166. Wilson LS, Pillay D, Kelly BD, Casey P. Mental health professionals and information sharing: carer perspectives. Ir J Med Sci. 2015;184(4): 781-790. doi: 10.1007/s11845-014-1172-6.
167. Cleary M, Hunt GE, Walter G, Freeman A. The patient’s view of need and caregiving consequences: A cross‐sectional study of inpatients with severe mental illness. J Psychiatr Ment Health Nurs. 2006;13(5): 506-514. doi: 10.1111/j.1365-2850.2006.00972.x.
168. Barnhart WR, Ellsworth DW, Robinson AC, Myers JV, Andridge RR, Havercamp SM. Caregiving in the shadows: National analysis of health outcomes and intensity and duration of care among those who care for people with mental illness and for people with developmental disabilities. Disabil Health J. 2020;13(1): 100837. doi: 10.1016/j.dhjo.2019.100837.
169. Pearce K, McGovern J, Barrowclough C. Assessment of need for psychosocial interventions in an Asian population of carers of patients with schizophrenia. J Adv Nurs. 2006;54(3): 284-292. doi: 10.1111/j.1365-2648.2006.03811.x.
170. Sono T, Oshima I, Ito J. Family needs and related factors in caring for a family member with mental illness: adopting assertive community treatment in Japan where family caregivers play a large role in community care. Psychiatry Clin Neurosci. 2008;62(5): 584-590. doi: 10.1111/j.1440-1819.2008.01852.x.
171. Hielscher E, Diminic S, Kealton J, Harris M, Lee YY, Whiteford H. Hours of Care and Caring Tasks Performed by Australian Carers of Adults with Mental Illness: Results from an Online Survey. Community Ment Health J. 2019;55(2): 279-295. doi: 10.1007/s10597-018-0244-x.
172. Ghosh S, Greenberg JS, Seltzer MM. Adaptation to a spouse’s disability by parents of adult children with mental illness or developmental disability. Psychiatr Serv. 2012;63(11): 1118-1124. doi: 10.1176/appi.ps.201200014.
173. Cirici Amell R, Cobo J, Castanyer MM, Giménez Gómez N. Gender and other factors influencing the burden of care in relatives of people diagnosed with schizophrenia and schizophrenia spectrum disorders. Int J Cult Ment Health. 2018;11(4): 638-652. doi: 10.1080/17542863.2018.1479764.
