# Supplementary material for: Assessing the impact of caregiving on informal caregivers of adults with a mental disorder in OECD countries: A systematic literature review of concepts and their respective questionnaires
Source: PLoS One. 2022 Jul 8;17(7):e0270278. doi: 10.1371/journal.pone.0270278 (PMC9269485; doi:10.1371/journal.pone.0270278)
Supplement: S1 File — (DOCX) [file pone.0270278.s003.docx]

# S2 File. Complete search string per database.

## Embase

(‘caregiver’/de OR ‘volunteer’/de OR ‘caregiver burden’/de OR ‘caregiver support’/de OR ‘Caregiver Strain Index’/de OR ‘caregiver burnout’/de OR ‘social care’/de OR (caregiver* OR care-giver* OR caregiver OR caregivers OR social-care* OR ((famil* OR friend* OR acquaintanc* OR spous* OR sibling* OR unpaid* OR uncompensat* OR volunt* OR informal OR relative OR relatives OR neighbor* OR neighbour* OR next-of-kin* OR dyad*) NEAR/2 (care* OR caring* OR health-care* OR healthcare* OR support)) OR ((famil* OR friend* OR acquaintanc* OR spous* OR sibling* OR relative OR relatives OR neighbor* OR neighbour* OR next-of-kin*) NEXT/1 (of) NEXT/1 (patient or patients)) OR ((patient*) NEXT/2 (famil* OR friend* OR acquaintanc* OR spous* OR sibling* OR relative OR relatives OR neighbor* OR neighbour* OR next-of-kin*))):ab,ti,kw) AND (‘mental disease’/mj/de OR ‘addiction’/mj/exp OR ‘drug dependence’/mj/exp OR ‘psychosis’/mj/exp OR ‘behavior disorder’/mj/exp OR ‘anxiety disorder’/mj/exp OR ‘mood disorder’/mj/exp OR ‘autism’/mj/exp OR ‘personality disorder’/mj/exp OR ‘psychosexual disorder’/mj/exp OR ‘sleep disorder’/mj/exp OR (addict* OR alcholism* OR psychosis OR schizophreni* OR ADHD OR PTSD OR anorex* OR bulimia OR pica OR kleptomani* OR cleptomani* OR pyromani* OR autism* OR autist* OR depression* OR ((mental* OR substanc* OR anxiet* OR behaviour* OR behavior* OR eating* OR attent*-deficit* OR impuls*-control* OR opposition*-defiant* OR mood OR affectiv* OR bipolar* OR obsessiv*-compuls* OR posttraumat* OR post-traumat* OR dissociativ* OR somatic-symptom* OR elimination* OR sleep-wake* OR conduct* OR personalit* OR paraphilic* OR psychosex* OR tic OR depress* OR psychotic*) NEAR/3 (diseas* OR disord* OR illness*)) OR ((drug OR substanc*) NEAR/3 (dependen*)) OR ((bing*) NEAR/3 (eat)) OR ((sexual* OR gender*) NEAR/3 (dysfunct* OR disord*)) OR ((languag*) NEAR/3 (disord* OR disabil*))):ti,kw) AND (‘questionnaire’/mj/exp OR ‘Caregiver Strain Index’/mj/de OR ‘interview’/mj/exp OR ‘rating scale’/mj/de OR ‘social support assessment’/mj/exp OR ‘quantitative analysis’/mj/de OR ‘scoring system’/mj/de OR ‘experience’/mj/de OR ‘outcome assessment’/mj/de OR ‘measurement’/mj/de OR ‘devices’/mj/de OR ‘caregiver burden’/de OR ‘stress’/mj/de OR ‘burnout’/mj/de OR ‘satisfaction’/mj/de OR (questionnair* OR survey* OR interview* OR donabedian* OR Impact* OR effects OR effect OR effectiveness OR experience OR experiences OR outcome* OR measure* OR instrument* OR assessment* OR appraisal* OR negative-aspect* OR burden* OR stress* OR burnout* OR positive-aspect* OR satisfaction* OR ((quantitativ* OR mixed-method*) NEAR/3 (study or studies OR research* OR analys*)) OR ((scoring OR rating) NEXT/2 (system OR systems OR scale*)) OR ((care OR caregiver OR caring OR caregivers OR caregiver* OR caregiving*) NEAR/3 (inventor* OR index OR indices OR rating OR scale*)) OR ((process) NEAR/3 (assessment* OR triad*))):ti,kw) NOT ((‘juvenile’/exp OR (juvenil* OR adolescen* OR preadolescen* OR youth* OR child* OR schoolchild* OR minors OR teen OR teens OR teenager* OR infan* OR toddler* OR pediatr* OR paediatr* OR puber* OR baby OR babies OR girl* OR boy* OR newborn* OR neonate* OR premature* OR pre-matur* OR kid OR kids OR underag* OR kindergar* OR pubescen* OR prepubesc* OR school* OR preschool* OR highschool* OR suckling OR PICU OR NICU OR PICUs OR NICUs):ab,ti,kw) NOT (‘adult’/exp OR (adult* OR aged OR elderl* OR man OR men OR woman OR women):ab,ti,kw)) AND [ENGLISH]/lim NOT ([Conference Abstract]/lim)

## Medline

(Caregivers/ OR Volunteers/ OR (caregiver* OR care-giver* OR caregiver OR caregivers OR social-care* OR ((famil* OR friend* OR acquaintanc* OR spous* OR sibling* OR unpaid* OR uncompensat* OR volunt* OR informal OR relative OR relatives OR neighbor* OR neighbour* OR next-of-kin* OR dyad*) ADJ2 (care* OR caring* OR health-care* OR healthcare* OR support)) OR ((famil* OR friend* OR acquaintanc* OR spous* OR sibling* OR relative OR relatives OR neighbor* OR neighbour* OR next-of-kin*) ADJ (of) ADJ (patient or patients)) OR ((patient*) ADJ2 (famil* OR friend* OR acquaintanc* OR spous* OR sibling* OR relative OR relatives OR neighbor* OR neighbour* OR next-of-kin*))).ab,ti,kf.) AND (Mental Disorders/ OR exp Behavior, Addictive/ OR exp Substance-Related Disorders/ OR exp Psychotic Disorders/ OR exp Anxiety Disorders/ OR exp Mood Disorders/ OR exp Autistic Disorder/ OR exp Personality Disorders/ OR exp Sexual Dysfunctions, Psychological/ OR exp Sleep Wake Disorders/ OR (addict* OR alcholism* OR psychosis OR schizophreni* OR ADHD OR PTSD OR anorex* OR bulimia OR pica OR kleptomani* OR cleptomani* OR pyromani* OR autism* OR autist* OR depression* OR ((mental* OR substanc* OR anxiet* OR behaviour* OR behavior* OR eating* OR attent*-deficit* OR impuls*-control* OR opposition*-defiant* OR mood OR affectiv* OR bipolar* OR obsessiv*-compuls* OR posttraumat* OR post-traumat* OR dissociativ* OR somatic-symptom* OR elimination* OR sleep-wake* OR conduct* OR personalit* OR paraphilic* OR psychosex* OR tic OR depress* OR psychotic*) ADJ3 (diseas* OR disord* OR illness*)) OR ((drug OR substanc*) ADJ3 (dependen*)) OR ((bing*) ADJ3 (eat)) OR ((sexual* OR gender*) ADJ3 (dysfunct* OR disord*)) OR ((languag*) ADJ3 (disord* OR disabil*))).ti,kf) AND (exp Surveys and Questionnaires/ OR exp Interviews as Topic/ OR Outcome and Process Assessment/ OR Outcome Assessment/ OR Stress, Psychological/ OR Burnout, Psychological/ OR Personal Satisfaction/ OR (questionnair* OR survey* OR interview* OR donabedian* OR Impact* OR effects OR effect OR effectiveness OR experience OR experiences OR outcome* OR measure* OR instrument* OR assessment* OR appraisal* OR negative-aspect* OR burden* OR stress* OR burnout* OR positive-aspect* OR satisfaction* OR ((quantitativ* OR mixed-method*) ADJ3 (study or studies OR research* OR analys*)) OR ((scoring OR rating) ADJ2 (system OR systems OR scale*)) OR ((care OR caregiver OR caring OR caregivers OR caregiver* OR caregiving*) ADJ3 (inventor* OR index OR indices OR rating OR scale*)) OR ((process) ADJ3 (assessment* OR triad*))).ti,kf) NOT ((exp Infant/ OR exp Child/ OR exp Adolescent/ OR (juvenil* OR adolescen* OR preadolescen* OR youth* OR child* OR schoolchild* OR minors OR teen OR teens OR teenager* OR infan* OR toddler* OR pediatr* OR paediatr* OR puber* OR baby OR babies OR girl* OR boy* OR newborn* OR neonate* OR premature* OR pre-matur* OR kid OR kids OR underag* OR kindergar* OR pubescen* OR prepubesc* OR school* OR preschool* OR highschool* OR suckling OR PICU OR NICU OR PICUs OR NICUs).ab,ti,kf.) NOT (exp Adult/ OR (adult* OR aged OR elderl* OR man OR men OR woman OR women).ab,ti,kf.)) AND eeling.la. NOT (news OR congres* OR abstract* OR book* OR chapter* OR dissertation abstract*).pt.

## Cochrane

((caregiver* OR care NEXT giver* OR caregiver OR caregivers OR social NEXT care* OR ((famil* OR friend* OR acquaintanc* OR spous* OR sibling* OR unpaid* OR uncompensat* OR volunt* OR informal OR relative OR relatives OR neighbor* OR neighbour* OR of NEXT kin* OR dyad*) NEAR/2 (care* OR caring* OR health NEXT care* OR healthcare* OR support)) OR ((famil* OR friend* OR acquaintanc* OR spous* OR sibling* OR relative OR relatives OR neighbor* OR neighbour* OR of NEXT kin*) NEXT/1 (of) NEXT/1 (patient or patients)) OR ((patient*) NEXT/2 (famil* OR friend* OR acquaintanc* OR spous* OR sibling* OR relative OR relatives OR neighbor* OR neighbour* OR of NEXT kin*))):ab,ti) AND ((addict* OR alcholism* OR psychosis OR schizophreni* OR ADHD OR PTSD OR anorex* OR bulimia OR pica OR kleptomani* OR cleptomani* OR pyromani* OR autism* OR autist* OR depression* OR ((mental* OR substanc* OR anxiet* OR behaviour* OR behavior* OR eating* OR attent* NEXT deficit* OR impuls* NEXT control* OR opposition* NEXT defiant* OR mood OR affectiv* OR bipolar* OR obsessiv* NEXT compuls* OR posttraumat* OR post NEXT traumat* OR dissociativ* OR somatic NEXT symptom* OR elimination* OR sleep NEXT wake* OR conduct* OR personalit* OR paraphilic* OR psychosex* OR tic OR depress* OR psychotic*) NEAR/3 (diseas* OR disord* OR illness*)) OR ((drug OR substanc*) NEAR/3 (dependen*)) OR ((bing*) NEAR/3 (eat)) OR ((sexual* OR gender*) NEAR/3 (dysfunct* OR disord*)) OR ((languag*) NEAR/3 (disord* OR disabil*))):ti) AND ((questionnair* OR survey* OR interview* OR donabedian* OR Impact* OR effects OR effect OR effectiveness OR experience OR experiences OR outcome* OR measure* OR instrument* OR assessment* OR appraisal* OR negative NEXT aspect* OR burden* OR stress* OR burnout* OR positive NEXT aspect* OR satisfaction* OR ((quantitativ* OR mixed NEXT method*) NEAR/3 (study or studies OR research* OR analys*)) OR ((scoring OR rating) NEXT/2 (system OR systems OR scale*)) OR ((care OR caregiver OR caring OR caregivers OR caregiver* OR caregiving*) NEAR/3 (inventor* OR index OR indices OR rating OR scale*)) OR ((process) NEAR/3 (assessment* OR triad*))):ti) NOT (((juvenil* OR adolescen* OR preadolescen* OR youth* OR child* OR schoolchild* OR minors OR teen OR teens OR teenager* OR infan* OR toddler* OR pediatr* OR paediatr* OR puber* OR baby OR babies OR girl* OR boy* OR newborn* OR neonate* OR premature* OR pre NEXT matur* OR kid OR kids OR underag* OR kindergar* OR pubescen* OR prepubesc* OR school* OR preschool* OR highschool* OR suckling OR PICU OR NICU OR PICUs OR NICUs):ab,ti) NOT ((adult* OR aged OR elderl* OR man OR men OR woman OR women):ab,ti))

## Web of Science

TS=(((caregiver* OR care-giver* OR caregiver OR caregivers OR social-care* OR ((famil* OR friend* OR acquaintanc* OR spous* OR sibling* OR unpaid* OR uncompensat* OR volunt* OR informal OR relative OR relatives OR neighbor* OR neighbour* OR next-of-kin* OR dyad*) NEAR/2 (care* OR caring* OR health-care* OR healthcare* OR support)) OR ((famil* OR friend* OR acquaintanc* OR spous* OR sibling* OR relative OR relatives OR neighbor* OR neighbour* OR next-of-kin*) NEAR/1 (of) NEAR/1 (patient or patients)) OR ((patient*) NEAR/2 (famil* OR friend* OR acquaintanc* OR spous* OR sibling* OR relative OR relatives OR neighbor* OR neighbour* OR next-of-kin*)))) NOT ((juvenil* OR adolescen* OR preadolescen* OR youth* OR child* OR schoolchild* OR minors OR teen OR teens OR teenager* OR infan* OR toddler* OR pediatr* OR paediatr* OR puber* OR baby OR babies OR girl* OR boy* OR newborn* OR neonate* OR premature* OR pre-matur* OR kid OR kids OR underag* OR kindergar* OR pubescen* OR prepubesc* OR school* OR preschool* OR highschool* OR suckling OR PICU OR NICU OR PICUs OR NICUs) NOT (adult* OR aged OR elderl* OR man OR men OR woman OR women))) AND TI=( ((addict* OR alcholism* OR psychosis OR schizophreni* OR ADHD OR PTSD OR anorex* OR bulimia OR pica OR kleptomani* OR cleptomani* OR pyromani* OR autism* OR autist* OR depression* OR ((mental* OR substanc* OR anxiet* OR behaviour* OR behavior* OR eating* OR attent*-deficit* OR impuls*-control* OR opposition*-defiant* OR mood OR affectiv* OR bipolar* OR obsessiv*-compuls* OR posttraumat* OR post-traumat* OR dissociativ* OR somatic-symptom* OR elimination* OR sleep-wake* OR conduct* OR personalit* OR paraphilic* OR psychosex* OR tic OR depress* OR psychotic*) NEAR/2 (diseas* OR disord* OR illness*)) OR ((drug OR substanc*) NEAR/2 (dependen*)) OR ((bing*) NEAR/2 (eat)) OR ((sexual* OR gender*) NEAR/2 (dysfunct* OR disord*)) OR ((languag*) NEAR/2 (disord* OR disabil*)))) AND ((questionnair* OR survey* OR interview* OR donabedian* OR Impact* OR effects OR effect OR effectiveness OR experience OR experiences OR outcome* OR measure* OR instrument* OR assessment* OR appraisal* OR negative-aspect* OR burden* OR stress* OR burnout* OR positive-aspect* OR satisfaction* OR ((quantitativ* OR mixed-method*) NEAR/2 (study or studies OR research* OR analys*)) OR ((scoring OR rating) NEAR/2 (system OR systems OR scale*)) OR ((care OR caregiver OR caring OR caregivers OR caregiver* OR caregiving*) NEAR/2 (inventor* OR index OR indices OR rating OR scale*)) OR ((process) NEAR/2 (assessment* OR triad*)))))

## PsycInfo

(Caregivers/ OR Volunteers/ OR Caregiver Burden/ OR (caregiver* OR care-giver* OR caregiver OR caregivers OR social-care* OR ((famil* OR friend* OR acquaintanc* OR spous* OR sibling* OR unpaid* OR uncompensat* OR volunt* OR informal OR relative OR relatives OR neighbor* OR neighbour* OR next-of-kin* OR dyad*) ADJ2 (care* OR caring* OR health-care* OR healthcare* OR support)) OR ((famil* OR friend* OR acquaintanc* OR spous* OR sibling* OR relative OR relatives OR neighbor* OR neighbour* OR next-of-kin*) ADJ (of) ADJ (patient or patients)) OR ((patient*) ADJ2 (famil* OR friend* OR acquaintanc* OR spous* OR sibling* OR relative OR relatives OR neighbor* OR neighbour* OR next-of-kin*))).ab,ti.) AND (Mental Disorders/ OR exp Substance Related and Addictive Disorders/ OR exp Psychosis OR exp Anxiety Disorders/ OR exp Affective Disorders/ OR exp Autism Spectrum Disorders/ OR exp Personality Disorders/ OR exp Sexual Function Disturbances/ OR exp Sleep Wake Disorders/ OR (addict* OR alcholism* OR psychosis OR schizophreni* OR ADHD OR PTSD OR anorex* OR bulimia OR pica OR kleptomani* OR cleptomani* OR pyromani* OR autism* OR autist* OR depression* OR ((mental* OR substanc* OR anxiet* OR behaviour* OR behavior* OR eating* OR attent*-deficit* OR impuls*-control* OR opposition*-defiant* OR mood OR affectiv* OR bipolar* OR obsessiv*-compuls* OR posttraumat* OR post-traumat* OR dissociativ* OR somatic-symptom* OR elimination* OR sleep-wake* OR conduct* OR personalit* OR paraphilic* OR psychosex* OR tic OR depress* OR psychotic*) ADJ3 (diseas* OR disord* OR illness*)) OR ((drug OR substanc*) ADJ3 (dependen*)) OR ((bing*) ADJ3 (eat)) OR ((sexual* OR gender*) ADJ3 (dysfunct* OR disord*)) OR ((languag*) ADJ3 (disord* OR disabil*))).ti) AND (exp Questionnaires/ OR exp Surveys/ OR exp Interviews/ OR Inventories/ OR exp Rating Scales/ OR Caregiver Burden/ OR Quantitative Methods/ OR Mixed Methods Research/ OR Stress/ OR Satisfaction/ OR (questionnair* OR survey* OR interview* OR donabedian* OR Impact* OR effects OR effect OR effectiveness OR experience OR experiences OR outcome* OR measure* OR instrument* OR assessment* OR appraisal* OR negative-aspect* OR burden* OR stress* OR burnout* OR positive-aspect* OR satisfaction* OR ((quantitativ* OR mixed-method*) ADJ3 (study or studies OR research* OR analys*)) OR ((scoring OR rating) ADJ2 (system OR systems OR scale*)) OR ((care OR caregiver OR caring OR caregivers OR caregiver* OR caregiving*) ADJ3 (inventor* OR index OR indices OR rating OR scale*)) OR ((process) ADJ3 (assessment* OR triad*))).ti) NOT (((juvenil* OR adolescen* OR preadolescen* OR youth* OR child* OR schoolchild* OR minors OR teen OR teens OR teenager* OR infan* OR toddler* OR pediatr* OR paediatr* OR puber* OR baby OR babies OR girl* OR boy* OR newborn* OR neonate* OR premature* OR pre-matur* OR kid OR kids OR underag* OR kindergar* OR pubescen* OR prepubesc* OR school* OR preschool* OR highschool* OR suckling OR PICU OR NICU OR PICUs OR NICUs).ab,ti.) NOT ((adult* OR aged OR elderl* OR man OR men OR woman OR women).ab,ti.)) AND eeling.la. NOT (news OR congres* OR abstract* OR book* OR chapter* OR dissertation abstract*).pt.

## Cinahl Plus

(MH Caregivers+ OR MH “Volunteer Workers+” OR MH “Caregiver Burden+” OR MH “Family Caregiver Status (Iowa NOC)+” OR TI (caregiver* OR care-giver* OR caregiver OR caregivers OR social-care* OR ((famil* OR friend* OR acquaintanc* OR spous* OR sibling* OR unpaid* OR uncompensat* OR volunt* OR informal OR relative OR relatives OR neighbor* OR neighbour* OR next-of-kin* OR dyad*) N2 (care* OR caring* OR health-care* OR healthcare* OR support)) OR ((famil* OR friend* OR acquaintanc* OR spous* OR sibling* OR relative OR relatives OR neighbor* OR neighbour* OR next-of-kin*) N1 (of) N1 (patient or patients)) OR ((patient*) N2 (famil* OR friend* OR acquaintanc* OR spous* OR sibling* OR relative OR relatives OR neighbor* OR neighbour* OR next-of-kin*))) OR AB (caregiver* OR care-giver* OR caregiver OR caregivers OR social-care* OR ((famil* OR friend* OR acquaintanc* OR spous* OR sibling* OR unpaid* OR uncompensat* OR volunt* OR informal OR relative OR relatives OR neighbor* OR neighbour* OR next-of-kin* OR dyad*) N2 (care* OR caring* OR health-care* OR healthcare* OR support)) OR ((famil* OR friend* OR acquaintanc* OR spous* OR sibling* OR relative OR relatives OR neighbor* OR neighbour* OR next-of-kin*) N1 (of) N1 (patient or patients)) OR ((patient*) N2 (famil* OR friend* OR acquaintanc* OR spous* OR sibling* OR relative OR relatives OR neighbor* OR neighbour* OR next-of-kin*)))) AND (MM “Mental Disorders+” OR MM “Behavior, Addictive+” OR MM “Substance Use Disorders+” OR MM “Psychotic Disorders+” OR MM “Anxiety Disorders+” OR MM “Affective Disorders+” OR MM “Autistic Disorder” OR MM “Personality Disorders+” OR MM “Psychosexual Disorders+” OR MM “Sleep Disorders+” OR TI (addict* OR alcholism* OR psychosis OR schizophreni* OR ADHD OR PTSD OR anorex* OR bulimia OR pica OR kleptomani* OR cleptomani* OR pyromani* OR autism* OR autist* OR depression* OR ((mental* OR substanc* OR anxiet* OR behaviour* OR behavior* OR eating* OR attent*-deficit* OR impuls*-control* OR opposition*-defiant* OR mood OR affectiv* OR bipolar* OR obsessiv*-compuls* OR posttraumat* OR post-traumat* OR dissociativ* OR somatic-symptom* OR elimination* OR sleep-wake* OR conduct* OR personalit* OR paraphilic* OR psychosex* OR tic OR depress* OR psychotic*) N2 (diseas* OR disord* OR illness*)) OR ((drug OR substanc*) N2 (dependen*)) OR ((bing*) N2 (eat)) OR ((sexual* OR gender*) N2 (dysfunct* OR disord*)) OR ((languag*) N2 (disord* OR disabil*)))) AND (MM Surveys OR MM “Research Instruments+” OR MM “Interviews+” OR MM “Outcome Assessment” OR MM “Stress, Psychological+” OR MM “Quantitative Studies” OR TI (questionnair* OR survey* OR interview* OR donabedian* OR Impact* OR effects OR effect OR effectiveness OR experience OR experiences OR outcome* OR measure* OR instrument* OR assessment* OR appraisal* OR negative-aspect* OR burden* OR stress* OR burnout* OR positive-aspect* OR satisfaction* OR ((quantitativ* OR mixed-method*) N2 (study or studies OR research* OR analys*)) OR ((scoring OR rating) N2 (system OR systems OR scale*)) OR ((care OR caregiver OR caring OR caregivers OR caregiver* OR caregiving*) N2 (inventor* OR index OR indices OR rating OR scale*)) OR ((process) N2 (assessment* OR triad*)))) NOT ((MH Child+ OR MH Adolescence+ OR TI (juvenil* OR adolescen* OR preadolescen* OR youth* OR child* OR schoolchild* OR minors OR teen OR teens OR teenager* OR infan* OR toddler* OR pediatr* OR paediatr* OR puber* OR baby OR babies OR girl* OR boy* OR newborn* OR neonate* OR premature* OR pre-matur* OR kid OR kids OR underag* OR kindergar* OR pubescen* OR prepubesc* OR school* OR preschool* OR highschool* OR suckling OR PICU OR NICU OR PICUs OR NICUs) OR AB (juvenil* OR adolescen* OR preadolescen* OR youth* OR child* OR schoolchild* OR minors OR teen OR teens OR teenager* OR infan* OR toddler* OR pediatr* OR paediatr* OR puber* OR baby OR babies OR girl* OR boy* OR newborn* OR neonate* OR premature* OR pre-matur* OR kid OR kids OR underag* OR kindergar* OR pubescen* OR prepubesc* OR school* OR preschool* OR highschool* OR suckling OR PICU OR NICU OR PICUs OR NICUs)) NOT (MH Adult+ OR TI (adult* OR aged OR elderl* OR man OR men OR woman OR women) OR AB (adult* OR aged OR elderl* OR man OR men OR woman OR women)))

## Econlit

AB,TI((caregiver* OR care-giver* OR caregiver OR caregivers OR social-care* OR famil* OR friend* OR acquaintanc* OR spous* OR sibling* OR unpaid* OR uncompensat* OR volunt* OR informal OR relative OR relatives OR neighbor* OR neighbour* OR next-of-kin* OR dyad*) AND (((addict* OR alcholism* OR psychosis OR schizophreni* OR ADHD OR PTSD OR anorex* OR bulimia OR pica OR autism* OR autist* OR depression* OR ((mental* OR substanc* OR anxiet* OR behaviour* OR behavior* OR eating* OR attent*-deficit* OR impuls*-control* OR opposition*-defiant* OR mood OR affectiv* OR bipolar* OR obsessiv*-compuls* OR posttraumat* OR post-traumat* OR dissociativ* OR somatic-symptom* OR elimination* OR sleep-wake* OR conduct* OR personalit* OR paraphilic* OR psychosex* OR tic OR depress* OR psychotic*) N/2 (diseas* OR disord* OR illness*))))) AND (((questionnair* OR survey* OR interview* OR donabedian* OR Impact* OR effects OR effect OR effectiveness OR experience OR experiences OR outcome* OR measure* OR instrument* OR assessment* OR appraisal* OR negative-aspect* OR burden* OR stress* OR burnout* OR positive-aspect* OR satisfaction* OR ((quantitativ* OR mixed-method*) N/2 (study or studies OR research* OR analys*))))))

## Google Scholar

caregiver|caregivers|caregiver|caregivers “mental|substance|anxiety|behaviour|behavior|eating|mood|affective|bipolar|posttraumatic|sleep|personality|depressive|psychotic disease|disorder|illness” questionnaire|questionnaires|survey|surveys|interview|interviews
