# Supplementary material for: Assessing the impact of caregiving on informal caregivers of adults with a mental disorder in OECD countries: A systematic literature review of concepts and their respective questionnaires
Source: PLoS One. 2022 Jul 8;17(7):e0270278. doi: 10.1371/journal.pone.0270278 (PMC9269485; doi:10.1371/journal.pone.0270278)
Supplement: S2 File — NR = not reported. (DOCX) [file pone.0270278.s004.docx]

# Meta-summary of caregiving burden (1/2)

| **Meta-summary of caregiving burden (1/2)** | | | | | | | | | | | | | | | | | |
| --- | --- | --- | --- | --- | --- | --- | --- | --- | --- | --- | --- | --- | --- | --- | --- | --- | --- |
| **Themes** | **Dimensions** | **Questionnaires** | | | | | | | | | | | | | | | |
|  |  | **1992-1993 Family Impact Study [1]** | **Burden Assessment Scale [2]** | **Burden Assessment Schedule [3]** | **Care-ED [4]** | **Caregiver Burden Inventory [5]** | **Caregiver Burden Scale [6]** | **Caregiver Strain Index [7]** | **Caregiver Strain Questionnaire [8]** | **Caregiver Strain Questionnaire- Short Form 7 [9]** | **Eating Disorder Symptom Impact Scale (EDSIS) [10]** | **ECFOS-II/SOFBI-II [11-12]** | **Family Burden and Care Participation Instrument [13]** | **Family Burden Interview Schedule [14]** | **Family Burden Questionnaire [15]** | **Family Burden Questionnaire [16]** | **Family Burden Scale [17]** |
| **Caregiver’s experiences with care recipient’s formal care recipients** |  |  |  |  |  |  |  |  |  |  |  |  |  |  |  |  |  |
|  | **Experiences of formal and informal compulsory care** |  |  |  |  |  |  |  |  |  |  |  | NR |  |  |  |  |
|  | **Experiences of the quality of care and attitudes towards compulsory care** |  |  |  |  |  |  |  |  |  |  |  | NR |  |  |  |  |
| **Perception of caregiving** | **Perception of caregiving** |  |  |  |  |  |  |  |  |  |  |  |  |  |  |  |  |
|  | **Self-perceived pressure** |  |  |  |  |  |  |  |  |  |  |  |  |  |  |  |  |
|  | **Role strain** |  |  |  |  |  |  |  |  |  |  |  |  |  |  |  |  |
| **Impact of caregiving on caregiver’s standard of living** | **Impact of caregiving on caregiver’s standard of living** |  |  |  |  |  |  |  |  |  |  |  |  |  |  |  |  |
|  | **Mental tension and disruption of private life** |  |  |  |  |  |  |  |  |  |  |  |  |  |  |  |  |
|  | **Impact of caregiving on daily routines** |  | Disrupted activities, including distractions, changed plans and household routines and reduced time for self, friends and other family members |  |  |  |  |  |  |  |  |  |  | Objective impact |  |  |  |
|  | **Effect on leisure** |  |  |  |  |  |  |  |  |  |  |  |  |  | Effect on family’s leisure | Stopping of normal recreational activities, absorption of another member’s holiday and leisure time, lack of participation by patient in leisure activity and planned leisure activity abandoned |  |
| **Global burden** | **Global burden** | Personal and familial demands associated with providing care, including irritation, sense of personal sacrifice, and worry that the family member could not function alone |  |  |  | Caregiver’s perception of the impact of caregiving on his/her social life, family relationships and time management | Includes the caregiver shouldering too much responsibility for the care recipient´s wellbeing, feeling tired and worn out, spending too much time with care recipient, and having insufficient time for self |  |  |  |  | The impact of care on functioning in the following caregiver’s life domains- work, social, relationships and leisure | Subjective and objective burden on relatives |  | NR |  |  |
| **Effect of caregiving on family** |  |  |  |  |  |  |  |  |  |  |  |  |  |  |  |  |  |
|  | **Disruption to household routine** |  |  |  |  |  |  |  |  |  |  |  |  |  |  |  |  |
|  | **Impact on marital relationship** |  |  | The disruption of the marital relationship as a consequence of the presence of the care recipient |  |  |  |  |  |  |  |  |  |  |  |  |  |
|  | **Family intrusions** |  |  |  |  |  |  |  |  |  |  |  |  |  |  |  |  |
|  | **Effect on mental health of other family members** |  |  |  |  |  |  |  |  |  |  |  |  |  |  | Any member seeking professional help for psychological illness and any member becoming depressed, weepy, and irritable |  |
|  | **Effect on family interaction** |  |  |  |  |  |  |  |  |  |  |  |  |  | NR | Ill effect on general family atmosphere, other members argue over the patient, reduction, or cessation of interaction with friends and neighbors, family becoming secluded or withdrawn and any other effect on family or neighborhood relationship |  |
|  | **Effect on family routine** |  |  |  |  |  |  |  |  |  |  |  |  |  | NR | Patient not attending work, school, etc., patient unable to help in household duties, disruption of activities due to patient´s illness and care, disruption of activities due to patient´s irrational demands and other family members missing school, meals |  |
|  | **Effect on children** |  |  |  |  |  |  |  |  |  |  |  | Situation of the underage children in the family |  | NR |  |  |
| **Care recipient characteristics** | **Care recipient characteristics** |  |  |  |  |  |  | Situations in the helper’s life that made it difficult to help; occurrence of situations that conflicted with giving help; satisfaction with the degree to which ex-patients understood caregivers’ problems and limitations; caregivers’ assistance received from others; emotional support and empathy received from others; and the degree to which helping had changed their lives. |  |  |  |  |  |  |  |  |  |
|  | **Care recipient’s passive behaviors** |  |  |  |  |  |  |  |  |  |  |  |  |  |  |  |  |
|  | **Perceived severity of the disease** |  |  | Severity of the disorder, such as disturbing or unpredictable behavior rending the caregiver unable to hold or take up a regular job |  |  |  |  |  |  |  |  |  |  |  |  |  |
|  | **Care recipient aggressiveness** |  |  |  |  |  |  |  |  |  |  |  |  |  |  |  | Captures the presence of episodes of hostility, violence, and destruction of property |
|  | **Care recipient dependence** |  |  |  |  |  |  |  |  |  |  |  |  |  |  |  |  |
|  | **Demand for attention** |  |  |  |  |  |  |  |  |  |  |  |  |  |  |  |  |
|  | **Admission to hospital** |  |  |  |  |  |  |  |  |  |  |  | NR |  |  |  |  |
| **Subjective burden** | **Subjective burden** |  |  |  |  |  |  |  |  | Psychological impact of caring |  | NR |  | Affective responses, including subjective worry and distress |  |  |  |
|  | **Subjective burden: externalized** |  |  |  |  |  |  |  | Negative feelings directed towards the care recipient (e.g., resentment, anger, embarrassment) |  |  |  |  |  |  |  |  |
|  | **Subjective burden: internalized** |  |  |  |  |  |  |  | Feelings internalized by the caregiver that are associated with caregiving (e.g., feeling sad or unhappy, worrying about the family’s future) |  |  |  |  |  |  |  |  |
| **Objective burden** | **Objective burden** |  |  |  |  |  |  |  | Negative occurrences that resulted from caring for a child with emotional or behavioral problems (e.g., interruption of personal time, missing work or neglecting duties, financial strain) | Establish how problematic issues have been over the past month |  |  |  |  |  |  |  |
| **Emotional burden** | **Emotional burden** |  |  |  |  | The negative feelings of the caregiver against the care recipient that are aroused by unusual or irritating behaviors of the care recipient | Includes feeling ashamed and embarrassed of care recipient´s behavior, feeling offended and angry with care recipient | Includes depression, anxiety, and hostility |  |  |  |  |  |  | Shame |  |  |
|  | **Frustration** |  |  |  |  |  |  |  |  |  |  |  |  |  |  |  |  |
|  | **Personal distress** |  | Distress that arises from experiencing frictions with persons outside the household, being embarrassed by disruptive behaviors, and feeling trapped and resentful |  |  |  |  |  |  |  |  |  |  |  | NR |  |  |
|  | **Guilt** |  | e.g., guilt for not helping enough, guilt for causing illness, worry about making illness worse |  |  |  |  |  |  |  | Includes feeling that the caregiver has let the caregiver down, thinking that there was something that the caregiver should have done |  |  |  |  |  |  |
|  | **Worry** |  |  |  |  |  |  |  |  |  |  |  |  |  | Caregiver worries for the care recipient |  |  |
|  | **Embarrassment** |  |  |  |  |  |  |  |  |  |  |  |  |  |  |  |  |
| **Caregiving tasks** |  |  |  |  |  |  |  |  |  |  |  |  |  |  |  |  |  |
|  | **Task: Caring for additional care recipient** |  |  |  |  |  |  |  |  |  |  |  |  |  |  |  |  |
|  | **Task: Time in transit** |  |  |  |  |  |  |  |  |  |  |  |  |  |  |  |  |
|  | **Task: Time spent on standby** |  |  |  |  |  |  |  |  |  |  |  |  |  |  |  |  |
|  | **Task: Assistance in daily living** |  |  |  |  |  |  |  |  |  |  | Worry, distress, frequency, and duration resulting from assistance with daily living tasks |  | Objective and subjective assistance in daily living | NR |  |  |
|  | **Task: Dealing with dysregulated behaviors** |  |  |  |  |  |  |  |  |  | Includes lying/stealing, food disappearing from the cupboards, bad smells, and poor hygiene in the bathroom |  |  |  |  |  |  |
|  | **Task: Emotional** |  |  |  | Time spent providing emotional support within the month before admission |  |  |  |  |  |  |  |  |  |  |  |  |
|  | **Task: Food** |  |  |  | Time spent providing food support within the month before admission |  |  |  |  |  | Includes difficulties preparing meals, arguments, and tension during mealtimes, checking on care recipient to ensure they are okay |  |  |  |  |  |  |
|  | **Task: Medical** |  |  |  | Time spent providing medical support within the month before admission |  |  |  |  |  |  |  |  |  |  |  |  |
|  | **Task: Non-food/medical practical support** |  |  |  | Time spent providing practical support within the month before admission |  |  |  |  |  |  |  |  |  |  |  |  |
|  | **Task: Obtaining information about eating disorders and local services** |  |  |  | Time spent obtaining information about eating disorders and local services within the month before admission |  |  |  |  |  |  |  |  |  |  |  |  |
|  | **Task: Participation in care** |  |  |  |  |  |  |  |  |  |  |  | NR |  |  |  |  |
|  | **Task: Supervising the patient** |  |  |  |  |  |  |  |  |  |  | Worry, distress, frequency, and duration of supervision of behavioral problems |  | Objective and subjective supervision of bothersome or troublesome behaviors | NR |  |  |
| **Positive effects of caregiving** |  |  |  |  |  |  |  |  |  |  |  |  |  |  |  |  |  |
|  | **Appreciation for caring** |  |  | Reflects the satisfaction caregivers receive from the appreciation and acknowledgement of their good care from friends and family members, and the pride of still being able to take good care of the rest of the family |  |  |  |  |  |  |  |  |  |  |  |  |  |
| **Economic burden** | **Economic burden** |  |  |  |  |  |  |  |  |  |  | Out-of-pocket expenses related to care |  | Objective money | NR | Includes loss of care recipient´s income, loss of income of other family members, expenses of patient´s illness, expenses due to other necessary changes in arrangements, loans taken and any other planned financial activity needing finance, postponed | Defined in terms of financial problems created by the care recipient’s illness |
| **Social burden** | **Social burden** |  | Includes significant alterations in the social spheres of work and family |  |  | Role conflicts that may be experienced in relationships with others and the restrictions in other roles because of caregiving, efforts of caregiving may not be welcomed by the care recipient | Isolation, which includes not inviting friends and acquaintances home because of care recipient´s problem, decreased social life |  |  |  | Social isolation, which includes losing friends, being unable to go out for evenings, weekend, or on holiday |  |  |  | Effect on social network |  | Defined in terms of burden experienced regarding disruption of daily/social activities |
|  | **Stigma** |  |  |  |  |  |  |  |  |  |  |  |  |  | NR |  |  |
|  | **Effect on others outside household** |  |  |  |  |  |  |  |  |  |  |  |  |  | NR |  |  |
|  | **Impact on relations with others** |  |  | Disruption of family and other social relations because of the presence of the care recipient |  |  |  |  |  |  |  |  |  |  |  |  |  |
| **Time dependence burden** | **Time dependence burden** |  | Negative temporal aspect of managing mental illness, such as past and lost possibilities for care recipients, present stigma, and future plans |  |  | Caregiver burden due to restrictions on time |  |  |  |  |  |  |  |  |  |  |  |
| **Negative effects of caregiving** |  |  |  |  |  |  |  |  |  |  |  |  |  |  |  |  |  |
|  | **Fear of accidents** |  |  |  |  |  |  |  |  |  |  |  |  |  |  |  |  |
|  | **Lack of pleasure in caring** |  |  |  |  |  |  |  |  |  |  |  |  |  |  |  |  |
|  | **Nervousness and restrictedness** |  |  |  |  |  |  |  |  |  |  |  |  |  |  |  |  |
|  | **Exhaustion with caregiving** |  |  |  |  |  |  |  |  |  |  |  |  |  |  |  |  |
|  | **Disappointment** |  |  |  |  |  | Includes feeling like life has treated the care recipient unfairly, expected that life would be different than it is at the caregiver´s age, experienced economic sacrifice related to caring, find it physically trying to take care of care recipient |  |  |  |  |  |  |  |  |  |  |
| **Subjective perception of the caregiving relationship** | **Subjective perception of the caregiving relationship** |  |  |  |  |  |  | Includes care recipient’s overall mental health status, rehospitalizations within 2 months since discharge from the hospital, and self-assessments of progress made |  |  |  |  |  |  |  |  |  |
|  | **Tension between caregiver and care recipient** |  |  |  |  |  |  |  |  |  |  |  |  |  |  |  |  |
| **Caregiver incompetence** | **Caregiver incompetence** |  |  |  |  |  |  |  |  |  |  |  |  |  |  |  |  |
| **Impact on wellbeing** | **Impact on wellbeing** |  |  | Impact of the presence of the care recipient on the caregiver in terms of feelings of exhaustion, frustration, depression, and impact on general health |  |  |  |  |  |  |  |  |  |  |  |  |  |
|  | **Psychological wellbeing** |  |  |  |  |  |  |  |  |  |  |  |  |  |  |  |  |
| **Physical burden** | **Physical burden** |  |  |  |  | Negative health effects of tiredness and sleep difficulties on the caregiver |  |  |  |  |  | Reflected by caregiver’s health status, use of health services and days lost at work because of health problems |  |  |  | Includes physical illness in any family member and any other adverse effect on others | Signs and symptoms of psychopathology reported by caregiver |
|  | **Sleep disturbance** |  |  |  |  |  |  |  |  |  |  |  |  |  |  |  |  |
|  | **Depression** |  |  |  |  |  |  |  |  |  |  |  |  |  |  |  |  |
| **Environmental burden** | **Environmental burden** |  |  |  |  |  | e.g., “the physical environment makes it troublesome for caregiver to take care of care recipient,” worry about not taking care of care recipient |  |  |  |  |  |  |  |  |  |  |
| **Effect on work/ employment** | **Effect on work/ employment** |  |  |  |  |  |  |  |  |  |  |  |  |  | NR |  |  |
| **Emotional support and psychosocial care** | **Emotional support and psychosocial care** |  |  |  |  |  |  |  |  |  |  |  | Relative’s own support received from the professionals |  |  |  |  |
| **Dedication to care and replacement of the caregiver by other caregivers** | **Dedication to care and replacement of the caregiver by other caregivers** |  |  |  |  |  |  |  |  |  |  | NR |  |  |  |  |  |

# Meta-summary of caregiving burden (2/2)

| **Meta-summary of caregiving burden (2/2)** | | | | | | | | | | | | | | | | | |
| --- | --- | --- | --- | --- | --- | --- | --- | --- | --- | --- | --- | --- | --- | --- | --- | --- | --- |
| **Themes** | **Dimensions** | **Questionnaires** | | | | | | | | | | | | | | | |
|  |  | **Family Problems Questionnaire [18]** | **Interview for Measuring the Burden on the Family [19]** | **Interview Schedule for Families and Relatives of Severely Mentally Ill Persons [20]** | **Perceived Burden Scale [21]** | **Perceived Family Burden Scale [22]** | **Schizophrenia Caregiver Questionnaire [23]** | **Self-developed questionnaire Goodman et al. [24]** | **Self-developed visual analogue scale by Heru & Ryan [25]** | **Self-developed questionnaire by Hielscher et al. [26]** | **Self-Perceived Pressure by Informal Care Scale [27]** | **Strain Scale [28]** | **Zarit Burden Interview [29]** | **Zarit Burden Interview [30]** | **Zarit Burden Interview [31]** | **Zarit Burden Interview [32]** | **Zarit Burden Interview [33]** |
| **Caregiver’s experiences with care recipient’s formal care recipients** |  |  |  |  |  |  |  |  |  |  |  |  |  |  |  |  |  |
|  | **Experiences of formal and informal compulsory care** |  |  |  |  |  |  |  |  |  |  |  |  |  |  |  |  |
|  | **Experiences of the quality of care and attitudes towards compulsory care** |  |  |  |  |  |  |  |  |  |  |  |  |  |  |  |  |
| **Perception of caregiving** | **Perception of caregiving** |  |  |  |  |  | e.g., “caregiver should be doing more for patient,” “unable to care for care recipient as much as liked” |  |  |  |  |  |  |  |  |  |  |
|  | **Self-perceived pressure** |  |  |  |  |  |  |  |  |  | e.g., “owing to the situation of my care recipient I have too little time for myself,” “combining the responsibility for my care recipient and for my job and/or family is not easy,” “my independence is suffering,” “the situation of my relative constantly demands my attention” |  |  |  |  |  |  |
|  | **Role strain** |  |  |  |  |  |  |  |  |  |  |  |  |  |  |  | Feeling embarrassed over relative´s behavior, social life suffered due to caregiving responsibilities, social life suffered |
| **Impact of caregiving on caregiver’s standard of living** | **Impact of caregiving on caregiver’s standard of living** |  |  |  |  |  |  | NR |  |  |  |  |  |  |  |  |  |
|  | **Mental tension and disruption of private life** |  |  |  |  |  |  |  |  |  |  |  |  | e.g., “Do you think you do not have enough time for yourself because of the time you are spending with your loved one?” “Caring for your close people and fulfilling other responsibilities related to your family or job- do you feel troubled by being interrupted?” “Do you feel that your health is impaired due to working with your loved ones?” “Do you think that you do not have the level of private life you want because of your relatives?” “Do you feel that your social life is disrupted because you look closer to you?” “Do you feel like you cannot control your life since your loved one got sick?” “Do you feel uncertain about what to do with your loved one?” |  |  |  |
|  | **Impact of caregiving on daily routines** |  |  |  |  |  |  |  |  |  |  |  |  |  |  |  |  |
|  | **Effect on leisure** |  |  | Effect of patient’s illness on leisure |  |  |  |  |  |  |  |  |  |  |  |  |  |
| **Global burden** | **Global burden** | Includes objective burden (e.g., at night one of us is forced to stay awake or to wake up often because of care recipient’s problems) and subjective (e.g., Looking after care recipient is physically tiring) |  |  |  |  | Humanistic impact, including physical, emotional, social, and daily life impact |  | NR |  |  |  | Subjective impact of the relationship |  |  | NR |  |
| **Effect of caregiving on family** |  |  |  |  |  |  |  |  |  |  |  |  |  |  |  |  |  |
|  | **Disruption to household routine** |  |  |  |  |  |  |  |  |  |  | NR |  |  |  |  |  |
|  | **Impact on marital relationship** |  |  |  |  |  |  | Impact of child’s illness on caregiver’s marriage |  |  |  |  |  |  |  |  |  |
|  | **Family intrusions** |  |  |  | NR |  |  |  |  |  |  |  |  |  |  |  |  |
|  | **Effect on mental health of other family members** |  |  |  |  |  |  |  |  |  |  |  |  |  |  |  |  |
|  | **Effect on family interaction** |  |  | Effect of patient’s illness on family interaction |  |  |  |  |  |  |  |  |  |  |  |  |  |
|  | **Effect on family routine** |  |  |  |  |  |  |  |  |  |  |  |  |  |  |  |  |
|  | **Effect on children** |  |  |  |  |  |  |  |  |  |  |  |  |  |  |  |  |
| **Care recipient characteristics** | **Care recipient characteristics** |  | Behaviour of the patient including assessment of symptomatology |  |  |  |  |  |  |  |  |  |  |  |  |  |  |
|  | **Care recipient’s passive behaviors** |  |  |  |  | Threatening suicide, inflicting self-harm, appearing fearful or sad |  |  |  |  |  |  |  |  |  |  |  |
|  | **Perceived severity of the disease** |  |  |  |  |  |  |  |  |  |  |  |  |  |  |  |  |
|  | **Care recipient aggressiveness** |  |  |  |  | Relative’s reaction to active/ aggressive behaviors, including refusing medicine, threatening violence, harassing others, talking loudly, using abusive language |  |  |  |  |  |  |  |  |  |  |  |
|  | **Care recipient dependence** |  |  |  |  |  | e.g., “care recipient asks for more help than needed,” “care recipient overly dependent to help with daily activities” |  |  |  |  |  |  | e.g., “Do you think your loved one is dependent on you?” “Do you feel that your loved one is expecting you to look at him, as if you were the only person he could lean on?” |  |  |  |
|  | **Demand for attention** |  |  |  |  |  |  |  |  |  |  | NR |  |  |  |  |  |
|  | **Admission to hospital** |  |  |  |  |  |  |  |  |  |  |  |  |  |  |  |  |
| **Subjective burden** | **Subjective burden** |  |  |  |  |  |  |  |  |  |  |  |  |  |  |  |  |
|  | **Subjective burden: externalized** |  |  |  |  |  |  |  |  |  |  |  |  |  |  |  |  |
|  | **Subjective burden: internalized** |  |  |  |  |  |  |  |  |  |  |  |  |  |  |  |  |
| **Objective burden** | **Objective burden** |  |  |  |  |  |  |  |  | Average weekly hours spent actively caring for main care recipient |  |  |  |  |  |  |  |
| **Emotional burden** | **Emotional burden** |  | Emotional atmosphere at home |  |  |  |  | Impact of child’s health on caregiver’s emotional health |  |  |  |  |  |  |  |  |  |
|  | **Frustration** |  |  |  |  |  |  |  |  |  |  | NR |  |  |  |  |  |
|  | **Personal distress** |  |  | NR | Personal strains |  |  |  |  |  |  |  |  |  |  |  | Distress that arises from experiencing frictions with persons outside the household, being embarrassed by disruptive behaviors and feeling trapped and resentful, wishing the care for the care recipient could be left to someone else |
|  | **Guilt** |  |  | NR |  |  |  |  |  |  |  |  |  |  |  |  |  |
|  | **Worry** |  |  | NR |  |  | e.g., “afraid of what the future holds,” “worry about patient having an episode” |  |  |  |  | NR |  |  |  |  |  |
|  | **Embarrassment** |  |  |  |  |  |  |  |  |  |  | NR |  |  |  |  |  |
| **Caregiving tasks** |  |  |  |  |  |  |  |  |  |  |  |  |  |  |  |  |  |
|  | **Task: Caring for additional care recipient** |  |  |  |  |  |  |  |  | Average weekly hours spent on additional care recipient |  |  |  |  |  |  |  |
|  | **Task: Time in transit** |  |  |  |  |  |  |  |  | Average weekly hours spent in transit |  |  |  |  |  |  |  |
|  | **Task: Time spent on standby** |  |  |  |  |  |  |  |  | Average weekly hours spent in standby |  |  |  |  |  |  |  |
|  | **Task: Assistance in daily living** |  | Buren due to the practical help |  |  |  |  |  |  | Average weekly hours spent on activities of daily living |  |  |  |  |  |  |  |
|  | **Task: Dealing with dysregulated behaviors** |  |  |  |  |  |  |  |  |  |  |  |  |  |  |  |  |
|  | **Task: Emotional** |  |  |  |  |  |  |  |  |  |  |  |  |  |  |  |  |
|  | **Task: Food** |  |  |  |  |  |  |  |  |  |  |  |  |  |  |  |  |
|  | **Task: Medical** |  |  |  |  |  |  |  |  |  |  |  |  |  |  |  |  |
|  | **Task: Non-food/medical practical support** |  |  |  |  |  |  |  |  | Average weekly hours spent on practical tasks |  |  |  |  |  |  |  |
|  | **Task: Obtaining information about eating disorders and local services** |  |  |  |  |  |  |  |  |  |  |  |  |  |  |  |  |
|  | **Task: Participation in care** |  |  |  |  |  |  |  |  |  |  |  |  |  |  |  |  |
|  | **Task: Supervising the patient** |  |  | NR |  |  |  |  |  |  |  |  |  |  |  |  |  |
| **Positive effects of caregiving** |  |  |  |  |  |  |  |  |  |  |  |  |  |  |  |  |  |
|  | **Appreciation for caring** |  |  |  |  |  |  |  |  |  |  |  |  |  |  |  |  |
| **Economic burden** |  | e.g., The family is forced to make financial sacrifices because of them |  |  |  |  |  |  |  |  |  |  |  | e.g., “Do you think you do not have enough money to look close to you in addition to your own expenses?” Do you think you need to do more for your loved one?” “Do you think I could do better in taking care of your loved one?” “Do you have any fears about the future of your loved one?” | e.g., “I feel that I don’t have enough money to support my spouse in addition to the rest of our expenses” |  |  |
| **Social burden** | **Social burden** |  | Consequences of exacerbation on the social life of the relative | Effect of patient’s illness on social network | Limitations on social life |  |  | Impact of child’s health on caregiver’s social life |  |  |  |  |  | Deterioration of social relationships, e.g. “Do you think that your loved one is currently negatively affecting your relationship with other family members or friends?” “Do you feel uncomfortable inviting your friends because of your relatives?” “Would you like to be able to leave the care of your loved one to someone else?” | e.g., “I feel that my social life has suffered because of my involvement with my spouse” |  |  |
|  | **Stigma** |  |  |  |  |  |  |  |  |  |  |  |  |  |  |  |  |
|  | **Effect on others outside household** |  |  |  |  |  |  |  |  |  |  |  |  |  |  |  |  |
|  | **Impact on relations with others** |  |  |  |  |  |  |  |  |  |  |  |  |  |  |  |  |
| **Time dependence burden** | **Time dependence burden** |  |  |  |  |  |  |  |  |  |  |  |  |  |  |  |  |
| **Negative effects of caregiving** |  |  |  |  |  |  |  |  |  |  |  |  |  |  |  |  |  |
|  | **Fear of accidents** |  |  |  |  |  |  |  |  |  |  | NR |  |  |  |  |  |
|  | **Lack of pleasure in caring** |  |  |  |  |  |  |  |  |  |  | NR |  |  |  |  |  |
|  | **Nervousness and restrictedness** |  |  |  |  |  |  |  |  |  |  |  |  | e.g., “Do you feel anger when you are with your loved one?” “Do you feel restricted when you are with your relative?” “How burdened do you feel about the care of your loved one when you take full advantage?” |  |  |  |
|  | **Exhaustion with caregiving** |  |  |  |  |  | Caregiver exhaustion with caregiving, e.g., “unable to take care of care recipient much longer,” “wish to leave care to someone else” |  |  |  |  |  |  |  |  |  |  |
|  | **Disappointment** |  |  |  |  |  |  |  |  |  |  |  |  |  |  |  |  |
| **Subjective perception of the caregiving relationship** | **Subjective perception of the caregiving relationship** |  | Relationship between the patient and the caregiver |  |  |  |  |  |  |  |  |  |  |  |  |  |  |
|  | **Tension between caregiver and care recipient** |  |  |  |  |  |  |  |  |  |  |  | Feelings of rejection/ hostility towards the care recipient |  | e.g., “I feel that my spouse tries to manipulate me” |  |  |
| **Caregiver incompetence** | **Caregiver incompetence** |  |  |  |  |  |  |  |  |  |  |  | Self-valuation of the incompetence to keep the care |  |  |  |  |
| **Impact on wellbeing** | **Impact on wellbeing** |  |  |  |  |  |  |  |  |  |  |  |  |  |  |  |  |
|  | **Psychological wellbeing** |  |  |  |  |  |  |  |  |  |  |  |  |  | e.g., “I feel nervous or depressed about my interaction with my spouse” |  |  |
| **Physical burden** | **Physical burden** |  |  |  |  |  |  | Impact of child’s health on caregiver’s physical health |  |  |  | NR |  |  | e.g., “I feel that my health has suffered because of my involvement with my spouse” |  |  |
|  | **Sleep disturbance** |  |  |  |  |  |  |  |  |  |  | NR |  |  |  |  |  |
|  | **Depression** |  |  |  |  |  |  |  |  |  |  | NR |  |  |  |  |  |
| **Environmental burden** | **Environmental burden** |  |  |  |  |  |  |  |  |  |  |  |  |  |  |  |  |
| **Effect on work/ employment** | **Effect on work/ employment** |  |  | Effect of patient’s illness on work/employment | Work interference |  |  | Impact of child’s health on caregiver’s career trajectory |  |  |  |  |  |  |  |  |  |
| **Emotional support and psychosocial care** | **Emotional support and psychosocial care** |  |  |  |  |  |  |  |  | Average weekly hours spent on emotional support and encouragement |  |  |  |  |  |  |  |
| **Dedication to care and replacement of the caregiver** | **Dedication to care and replacement of the caregiver** |  |  |  |  |  |  |  |  |  |  |  |  |  |  |  |  |

# Meta-summary of caregiving needs

| **Meta-summary of caregiving needs** | | | | | | | | | | |
| --- | --- | --- | --- | --- | --- | --- | --- | --- | --- | --- |
| **Themes** | **Dimensions** | **Questionnaires** | | | | | | | | |
|  |  | **Behavioral Risk Factor Surveillance System [34]** | **Caregiver Needs Survey [35]** | **Caregivers' Needs Assessment for Schizophrenia [36]** | **General Social Survey Questionnaire [37]** | **Relative's Cardinal Needs Schedule [38]** | **Relatives' Urgent Needs Schedule- Early Intervention [39]** | **Self-developed questionnaire by Sono et al. [40]** | **Self-developed questionnaire by Chamba et al. [41]** | **Family Needs Questionnaire [42]** |
| **Caregiver needs assessment** | **Caregiver needs assessment** |  | Caregiver needs and perceptions, including difficulties or delays in accessing services due to ineligibility; unavailability; waitlists; cost; lack of information; and other difficulties, as well as their levels of frustration in getting services for their children | Caregiver needs regarding not having enough information on the illness, its symptoms and course, treatment, rehabilitation and relapses and their prevention, communication problems with professionals and the patient, fear of stigmatization and discrimination, problems caused by relapses or crises, burden caused by non-compliance or dangerous behavior of the patient; caregiver´s stress due to earlier life events, financial burden, disappointment caused by the chronic course of the illness, feelings of guilt, not enough time for oneself, social isolation, conflicts within the family, over-involvement, difficulties because the patient lives in the same apartment, burden for minor children or siblings of the patient and burn-out or illness of the caregiver |  |  |  |  | Unmet needs of caregivers, including break from caring for the person, help planning for the person’s future, help getting care for the person in the future, someone to help show what services are available, more money to care for the person, to expand my education, skills and interests, to do things that I enjoy, help getting respite, help exploring future educational options for person, spend more time with partner, learn the best ways of helping person, to have emergency social care available, help getting a day service placement, to have emergency health care available, help in the holidays, help at community and family events, spend more time with other children |  |
| **Community support network** | **Community support network** |  |  |  |  |  |  |  |  | e.g., "I need to have other family members understand the patient's problems" |
| **Needs related to care recipient** |  |  |  |  |  |  |  |  |  |  |
|  | **Needs: Affective symptoms** |  |  |  |  | Cardinal need for help coping with affective symptoms | Issues relating to affective difficulties that the caregiver has found distressing over the previous 12 months and requires help for |  |  |  |
|  | **Needs: Antisocial behavior** |  |  |  |  | Cardinal need for help coping with antisocial behavior |  |  |  |  |
|  | **Needs: Constructive** |  |  |  |  |  | Issues relating to constructive difficulties that the caregiver has found distressing over the previous 12 months and requires help for |  |  |  |
|  | **Needs: Disruptive** |  |  |  |  |  | Issues relating to disruptive difficulties that the caregiver has found distressing over the previous 12 months and requires help for |  |  |  |
|  | **Needs: Negative symptoms** |  |  |  |  | Cardinal need for help coping with negative symptoms |  |  |  |  |
|  | **Needs: Psychotic symptoms** |  |  |  |  | Cardinal need for help coping with psychotic symptoms | Issues relating to psychosis that the caregiver has found distressing over the previous 12 months and requires help for |  |  |  |
|  | **Needs: Relapse prevention** |  |  |  |  | Cardinal need for relapse prevention |  |  |  |  |
|  | **Needs: Involvement in care** |  |  |  |  |  |  |  |  | e.g., "I need to be told daily what is being done with or for the patient" |
|  | **Needs: Monitoring of early warning signs** |  |  |  |  |  | Issues relating to the monitoring of early warning signs that the caregiver has found distressing over the previous 12 months and requires help for |  |  |  |
| **Needs related to information** | **Needs related to information** |  |  |  |  |  | Issues relating to information need that the caregiver has found distressing over the previous 12 months and requires help for |  |  |  |
|  | **Needs: Information about condition and treatment** |  |  |  |  | Cardinal need for information about illness |  |  |  | Health information, e.g. "I need to have explanations from professionals given in terms I can understand" |
| **Needs related to personal life** |  |  |  |  |  |  |  |  |  |  |
|  | **Needs: Interpersonal problems** |  |  |  |  | Cardinal need for help coping with interpersonal problems | Issues relating to interpersonal difficulties that the caregiver has found distressing over the previous 12 months and requires help for |  |  |  |
|  | **Needs: Relationship difficulties** |  |  |  |  | Cardinal need for help with relationship difficulties | Difficulties relating to relationship issues that the caregiver has found distressing over the previous 12 months and requires help for |  |  |  |
|  | **Needs: Social and leisure activities** |  |  |  |  | Cardinal need for help regarding reduced social and leisure activities | Difficulties relating to social issues that the caregiver has found distressing over the previous 12 months and requires help for |  |  |  |
|  | **Needs: Work/study** |  |  |  |  | Cardinal need for help with work or employment disruption | Issues relating to work/study that the caregiver has found distressing over the previous 12 months and requires help for |  |  |  |
| **Needs related to caregiver support services** | **Needs related to caregiver support services** | Classes about giving care, such as giving meds; help in getting access to services; support groups; individual counseling to help cope with giving care; respite care |  |  | Homecare, financial support, information, emotional support, help from medical professionals, occasional relief/ respite care, voluntary/ community services |  |  |  |  |  |
|  | **Needs: Current for assertive community treatment** |  |  |  |  |  |  | Caregiver current assertive community treatment needs, including services provided by multi-disciplinary team, services provided in consumers' home or community, counseling for anxieties and troubles, housing services, support for admission to hospital, service to maintain daily living, explanation or advice about medication, vocational services, |  |  |
|  | **Needs: Domestic task** |  |  |  |  | Cardinal need for help regarding increased household tasks | Issues relating to domestic tasks that the caregiver has found distressing over the previous 12 months and requires help for |  |  |  |
|  | **Needs: Emotional support** |  |  |  |  |  | Issues relating to emotional experience difficulties that the caregiver has found distressing over the previous 12 months and requires help for |  |  | e.g., "I need help in remaining hopeful about the patient's future" |
|  | **Needs: Future for assertive community treatment** |  |  |  |  |  |  | Caregiver future assertive community treatment needs, including services provided by multi-disciplinary team, services provided in consumers' home or community, counseling for anxieties and troubles, housing services, support for admission to hospital, service to maintain daily living |  |  |
|  | **Needs: Instrumental support** |  |  |  |  |  |  |  |  | e.g., "I need to get enough rest or sleep" |
|  | **Needs: Professional support** |  |  |  |  | Cardinal need for professional support |  |  |  | e.g., "I need to have help in deciding how much to let the patient do by himself/herself" |
| **Needs related to finances** | **Needs related to finances** |  |  |  |  | Cardinal need for help regarding adverse financial consequences | Issues relating to financial difficulties that the caregiver has found distressing over the previous 12 months and requires help for |  |  |  |

# Meta-summary of caregiver service use

| **Meta-summary of caregiver service use** | | | | | | | |
| --- | --- | --- | --- | --- | --- | --- | --- |
| **Themes** | **Dimensions** | **Questionnaires** | | | | | |
|  |  | **2010, 2011, and 2013 EU5 National Health and Wellness Survey [43]** | **Client Service Receipt Inventory- Service Receipt section [44]** | **Family Support Scale [45]** | **Insurance-Medicine-All-Sweden (IMAS) study [46]** | **Medical Expenditure Panel Survey [47]** | **Self-developed questionnaire by Perlick et al. [48]** |
| **Overall service use** | **Overall service use** |  | Includes hospital in-patient days, out-patient/ day care attendances, community-based service contacts (mental health, social services, and primary care), criminal justice service contacts |  |  |  |  |
|  | **Caregiver support use** |  |  | Includes utility of the following services: parents, spouse’s parents, relatives/ kin, spouse’s relatives, spouse, friends, spouse’s friends, children, other parents, co-workers, parent groups, social groups, church, family/child’s physician, early intervention. Program, school/ daycare, professional help, professional agency |  |  |  |
| **Medical care use** | **Medical care use** | Includes health care provider visits (e.g., general practitioner, internist, cardiologist, neurologist; psychiatrist), emergency room visits, and number of times hospitalized |  |  |  | Includes emergency room visits, hospitalizations, and healthcare provider visits |  |
|  | **Mental health service use** |  |  |  | Number of in- and specialized outpatient care visits due to psychiatric disorders (ICD-10 codes: F00-F99); |  | Frequency of mental health service usage in the past 7 months |
|  | **Primary care service use** |  |  |  |  |  | Frequency of primary care service usage in the past 7 months |
|  | **Somatic health service use** |  |  |  | Number of in- and specialized outpatient care visits due to somatic disorders (i.e., diabetes mellitus type 2, diseases of the circulatory system, diseases of esophagus, stomach, and duodenum, liver disease, and dorsalis); |  |  |

# Meta-summary of caregiver characteristics

| **Meta-summary of caregiver characteristics** | | | | | |
| --- | --- | --- | --- | --- | --- |
| **Themes** | **Dimensions** | **Questionnaires** | | | |
|  |  | **Christian Faith Practices Scale [49]** | **Intention to Care Scale [50]** | **Self- and Sibling-Care Measure [51]** | **Sense of Coherence Index [52]** |
| **Sense of coherence** |  |  |  |  |  |
|  | **Comprehensibility of situation** |  |  |  | Ability of people to understand what is happening around them |
|  | **Manageability** |  |  |  | Extent to which they feel capable of managing the situation |
|  | **Meaningfulness** |  |  |  | Ability to find meaning in the situation |
| **Sibling-related characteristics** |  |  |  |  |  |
|  | **Balanced sibling-care priorities** |  |  | Balance in self- and sibling-care |  |
| **Devotional practices** | **Devotional practices** | e.g., attend weekly worship services |  |  |  |
|  | **Relating** | e.g., confess my faults to others |  |  |  |
| **Self-care priorities** | **Self-care priorities** |  |  | Priority toward self-care |  |
| **Intention to care** | **Intention to care** |  |  |  |  |
|  | **Emotional support** |  | Assistance area: emotional support (e.g., I intend/expect to help my sibling with emotional support sometime in the future) |  |  |
|  | **Basic needs** |  | Assistance area: basic needs |  |  |
|  | **Financial assistance** |  | Assistance area: financial assistance |  |  |
|  | **Symptom management** |  | Assistance area: symptom management |  |  |
|  | **Arranging for supportive services** |  | Assistance area: arranging for supportive services |  |  |
| **Serving** | **Serving** | e.g., volunteer to help those less fortunate |  |  |  |
| **Ambivalence** | **Ambivalence** |  |  | Ambivalence toward sibling caregiving |  |

# Meta-summary of mental illness conceptions

| **Meta-summary of mental illness conceptions** | | | | | | | |
| --- | --- | --- | --- | --- | --- | --- | --- |
| **Themes** | **Dimensions** | **Questionnaires** | | | | | |
|  |  | **Anorectic Behaviour Observation Scale [53]** | **Illness Perceptions Questionnaire for Schizophrenia- Relatives’ version [54]** | **Knowledge Measure [55]** | **Mental Health Knowledge Schedule [56]** | **Mental Illness and Disorder Understanding Scale [57]** | **Self-developed vignette by Link et al. [58]** |
| **Caregiver perception of eating disorder** |  |  |  |  |  |  |  |
|  | **Bulimic behaviour** | Refers to bulimic-like behavior |  |  |  |  |  |
|  | **Concern about diet** | Refers to eating behavior, concern with weight and food, denial of problems |  |  |  |  |  |
|  | **Hyperactivity** | Refers to physical hyperactivities (e.g., engaging in a lot of sports, leaving the table during mealtimes) |  |  |  |  |  |
| **Caregiver perception of illness consequences** |  |  |  |  |  |  |  |
|  | **Consequences for the patient** |  | Perception of negative consequences for the patient |  |  |  |  |
|  | **Consequences for the relative** |  | Perception of negative consequences for the relative |  |  |  |  |
| **Caregiver perception of illness** |  |  |  |  |  |  |  |
|  | **Identity** |  | The number of experiences that relatives recognize as ever having been a problem for the patient since the onset of their mental health problems |  |  |  |  |
|  | **Illness coherence** |  | Sense of having no coherent understanding of the mental health problems |  |  |  |  |
| **Caregiver perception of personal blame** |  |  |  |  |  |  |  |
|  | **Personal blame- patient** |  | Attributions of blame towards the patient |  |  |  |  |
|  | **Personal blame- relative** |  | Attributions of blame towards the relative |  |  |  |  |
|  | **Personal control- patient** |  | Perception of control by the patient |  |  |  |  |
|  | **Personal control- relative** |  | Perception of control by the relative |  |  |  |  |
| **Emotional representation** | **Emotional representation** |  | Negative emotional response to the patient’s mental health problems |  |  |  |  |
| **Knowledge of mental illness conditions** | **Knowledge of mental illness conditions** |  |  | NR | Levels of recognition and familiarity with various conditions |  |  |
|  | **Cause** |  | Relative’s beliefs concerning the causal factor in the patient’s mental health problems |  |  |  |  |
|  | **Efficacy of medication** |  |  |  |  | Includes knowing that initial treatment requires medication, medication improves brain function, medication is effective in improving symptoms, continuous administration of medication does not lead  to intoxication or addiction, mental illness is a brain disease |  |
|  | **Recognition of mental illness** |  |  |  |  |  | NR |
|  | **Social recognition of illness** |  |  |  |  | Includes knowing that mental illnesses are very common, anybody can suffer from mental illness, mental illness is a medical condition like other illnesses, people with mental disorders are able to live in the community, if they receive appropriate support |  |
|  | **Stigma-related mental health knowledge** |  |  |  | Includes help seeking, recognition, support, employment, treatment, and recovery |  |  |
|  | **Timeline acute/chronic** |  | Perception of chronic timeline |  |  |  |  |
|  | **Timeline cyclical** |  | Perception of a more cyclical pattern of mental health problems over time |  |  |  |  |
|  | **Treatability of mental illness** |  |  |  |  | Includes knowing that mental illness requires early treatment, mental illness is treatable, delayed treatment worsens the prognosis of mental illness, living environment has an influence on recovery from illness, misunderstanding of mental illnesses makes it difficult for people with mental disorders to participate in society, rehabilitation is effective in improving mental illness |  |
|  | **Treatment control/ cure** |  | Belief that treatment can be effective in alleviating the mental health problems |  |  |  |  |
| **Stigma** |  |  |  |  |  |  |  |
|  | **Beliefs about how dangerous people with mental illnesses are** |  |  |  |  |  | NR |
|  | **Beliefs about the causes of mental illness** |  |  |  |  |  | NR |
|  | **The amount of social distance desired from people with mental illness** |  |  |  |  |  | NR |

# Meta-summary of family impact

| **Meta-summary of family impact** | | | | | | | | | | | | | | | | | |
| --- | --- | --- | --- | --- | --- | --- | --- | --- | --- | --- | --- | --- | --- | --- | --- | --- | --- |
| **Themes** | **Dimensions** | **Questionnaires** | | | | | | | | | | | | | | | |
|  |  | **Adult Sibling Relationship Questionnaire [59]** | **Family Questionnaire [60]** | **Family Attitudes Scale [61]** | **Level of Expressed Emotion [62]** | **Family Problems Questionnaire [18]** | **Attitudes Towards Schizophrenia Questionnaire for Relatives [63]** | **Family Assessment Device [64]** | **Family Communication Scale [65]** | **Family Empowerment Scale [66]** | **Family Experiences Interview Schedule [67]** | **Family Quality of Life Survey [68]** | **Self-developed visual analogue scale by Heru & Ryan [69]** | **Self-developed visual analogue scale by Heru et al. [70]** | **Role Behavior Inventory [71]** | **Family Strengths Scale [72]** | **Parent Experience of Chronic Illness [73]** |
| **Financial expenditures** | **Financial expenditures** |  |  |  |  |  |  |  |  |  | Objective money, including paying for and giving money to care recipient for transportation, food, mental health treatment and personal items |  |  |  |  |  |  |
| **Characteristics of parental caregivers** |  |  |  |  |  |  |  |  |  |  |  |  |  |  |  |  |  |
|  | **Emotional resources** |  |  |  |  |  |  |  |  |  |  |  |  |  |  |  | i.e., resources that parents have available to contend with the illness |
|  | **Guilt and worry** |  |  |  |  |  |  |  |  |  |  |  |  |  |  |  | i.e., concerns about the care recipient's current and future well-being and distress about the caregiver's role in the illness |
|  | **Unresolved sorrow and anger** |  |  |  |  |  |  |  |  |  |  |  |  |  |  |  | i.e., emotions related to having a child with a chronic illness |
| **Long-term uncertainty** | **Long-term uncertainty** |  |  |  |  |  |  |  |  |  |  |  |  |  |  |  | i.e., concerns about the impact of the illness on the care recipient |
| **Family role** | **Family role** |  |  |  |  |  |  | Focuses on whether the family has established patterns of behavior for handling a set of family functions |  |  |  |  |  |  |  |  |  |
|  | **Hero role** |  |  |  |  |  |  |  |  |  |  |  |  |  | Characterized as people who are goal-driven, achievement-oriented, mature, serious and have high standards |  |  |
|  | **Lost child role** |  |  |  |  |  |  |  |  |  |  |  |  |  | Characterized as people who are self-conscious and sensitive |  |  |
|  | **Mascot role** |  |  |  |  |  |  |  |  |  |  |  |  |  | Characterized as people who are carefree and entertaining |  |  |
|  | **Scapegoat role** |  |  |  |  |  |  |  |  |  |  |  |  |  | Characterized as people who act out and do not conform |  |  |
|  | **Enabler role** |  |  |  |  |  |  |  |  |  |  |  |  |  | Characterized as people who are involved in taking care of the house |  |  |
| **Expressed emotion** |  |  |  |  |  |  |  |  |  |  |  |  |  |  |  |  |  |
|  | **Warmth** | Intimacy, affection, knowledge, acceptance, similarity, admiration, emotional support, instrumental support |  |  |  |  |  |  |  |  |  |  |  |  |  |  |  |
|  | **Emotional overinvolvement** |  | Unusually over-intrusive, self-sacrificing,  overprotective, or devoted behavior, exaggerated emotional response, overidentification with the care recipient |  |  | e.g., "We feel guilty about the origin of their problems" |  |  |  |  |  |  |  |  |  |  |  |
|  | **Hostility** |  |  | NR |  |  |  |  |  |  |  |  |  |  |  |  |  |
|  | **Lack of emotional support** |  |  |  | The (lack of) emotionally  supportive behavior by the significant other regarding the subject and his/her problems, includes emotional responsibility, expectancy tolerance and attitude versus illness |  |  |  |  |  |  |  |  |  |  |  |  |
|  | **Irritation** |  |  |  | e.g., "fly off the handle when care recipient doesn't do something well" |  |  |  |  |  |  |  |  |  |  |  |  |
|  | **Intrusiveness** |  |  |  | Tendency of the significant other to get upset or irritable when things go wrong |  |  |  |  |  |  |  |  |  |  |  |  |
|  | **Critical attitudes** |  | Constructive criticism, an unfavorable comment on the behavior or the personality of the care recipient | Level of criticism |  | e.g., "Sometimes I criticize them or nag them about their behavior" |  |  |  |  |  |  |  |  |  |  |  |
| **Sibling relationship** |  |  |  |  |  |  |  |  |  |  |  |  |  |  |  |  |  |
|  | **Conflict** | Includes dominancy, competition, antagonism, quarreling |  |  |  |  |  |  |  |  |  |  |  |  |  |  |  |
|  | **Rivalry** | Includes maternal rivalry, paternal rivalry |  |  |  |  |  |  |  |  |  |  |  |  |  |  |  |
| **Responses to caregiving** |  |  |  |  |  |  |  |  |  |  |  |  |  |  |  |  |  |
|  | **Behavioral responses** |  |  |  |  |  | Defined as behavioral intentions or action predispositions |  |  |  |  |  |  |  |  |  |  |
|  | **Cognitive responses** |  |  |  |  |  | Defined as the beliefs, opinions, and ideas about the attitude object |  |  |  |  |  |  |  |  |  |  |
|  | **Affective responses** |  |  |  |  |  | Defined as the assessment of feelings as pleasant or unpleasant | Assesses the extent to which individual family members can experience appropriate affect over a range of stimuli. Both welfare and emergency emotions |  |  | Subjective worry and distress |  |  |  |  |  |  |
| **Caregiving tasks** |  |  |  |  |  |  |  |  |  |  |  |  |  |  |  |  |  |
|  | **Assistance in daily living** |  |  |  |  |  |  |  |  |  | Objective and subjective assistance in daily living care, including grooming, bathing, and dressing, reminding care recipient to take medicine, housework, cooking, and laundry |  |  |  |  |  |  |
|  | **Supervision of bothersome or troublesome behaviors** |  |  |  |  |  |  |  |  |  | Objective and subjective supervision, including trying to prevent or stop care recipient from doing something embarrassing and making excessive demands for attention |  |  |  |  |  |  |
|  | **Impact on daily routines** |  |  |  |  |  |  |  |  |  | Objective impact, including disrupted social and leisure activities |  |  |  |  |  |  |
| **Family functioning** | **Family functioning** |  |  |  |  |  |  | Assesses the overall health/ pathology of the family |  |  |  | Family interaction, which includes the family enjoying spending time together, talking openly with each other, solving problems together, supporting each other to accomplish goals, showing that they love and care for each other, and being able to handle life's ups and downs | NR | NR |  |  |  |
|  | **Affective involvement** |  |  |  |  |  |  | Extent to which family members are interested in and place value on each other’s activities and concerns |  |  |  |  |  |  |  |  |  |
|  | **Behavior control** |  |  |  |  |  |  | Assesses the way in which a family expresses and maintains standards for the behavior of its members. Behavior in situations of different sorts is assessed as are different patterns of control |  |  |  |  |  |  |  |  |  |
|  | **Problem solving** |  |  |  |  |  |  | Refers to the family’s ability to resolve problems (issues which threaten the integrity and functional capacity of the family) at a level that maintains effective family functioning |  |  |  |  |  |  |  |  |  |
|  | **Adaptability and flexibility** |  |  |  |  |  |  |  |  |  |  |  |  |  |  | e.g., "we compromise when we need to" |  |
|  | **Family cohesion** |  |  |  |  |  |  |  |  |  |  |  |  |  |  | e.g., "we can depend on each other" |  |
|  | **Community connections** |  |  |  |  |  |  |  |  |  |  |  |  |  |  | e.g., "we seek help when we need it from outside the family" |  |
|  | **Companionship** |  |  |  |  |  |  |  |  |  |  |  |  |  |  | e.g., "our family spends time together with friends or neighbors" |  |
|  | **Physical/ Material well-being** |  |  |  |  |  |  |  |  |  |  | Includes the family getting medical care when needed, getting dental care when needed, family members having transportation to get to the places they need to be, having a way to take care of their expenses, and feeling safe at home, work, school, and in the neighborhood |  |  |  |  |  |
|  | **Parenting** |  |  |  |  |  |  |  |  |  |  | Includes helping children learn to be independent, helping children with schoolwork and activities, teaching the children how to get along with others, teaching the children to make good decisions, adults in the family knowing other people in the children's lives, and adults in my family have time to take care of the individual needs of every child |  |  |  |  |  |
|  | **Emotion** |  |  |  |  |  |  |  |  |  |  | Emotional wellbeing, including the family having the support we need to relieve stress, have friends or others who provide support, have some time to pursue their own interests, and has outside help available to us to take care of special needs of all family members |  |  |  |  |  |
|  | **Empowerment with respect to the family, service system and larger community and political environment** |  |  |  |  |  |  |  |  | Level of empowerment in the immediate situation at home, professionals and agencies that provide services to the caregiver's own child and the legislative bodies, policy makers, agencies and community members who are concerned with or who influence services for children with disorders and their families |  |  |  |  |  |  |  |
|  | **Expression of empowerment as attitudes, knowledge, and behaviors** |  |  |  |  |  |  |  |  | Concerns what a caregiver feels and believes, what a caregiver knows and can potentially do and what a caregiver actually does, e.g., "Professionals should ask me what services I want for my child." |  |  |  |  |  |  |  |
| **Communication** | **Communication** |  |  |  |  |  |  | Exchange of information among family members. The focus is on whether verbal messages are clear with respect to content and direct in the sense that the person spoken to is the person for whom the message is intended | Functionality of communication within the family |  |  |  |  |  |  |  |  |
|  | **Conflictual communications** |  |  |  |  |  |  |  |  |  |  |  |  |  |  | e.g., "when we are angry, we can talk it out rather than yelling, grabbing, slapping, hitting or throwing things at one another" |  |
| **Disability-related support** | **Disability-related support** |  |  |  |  |  |  |  |  |  |  | Includes the family member with special needs having support to make progress at school, workplace, home, and make friends and the family having a good relationship with the service providers who work with the family member with a disability |  |  |  |  |  |

# Meta-summary of mental health (1/2)

| **Meta-summary of mental health (1/2)** | | | | | | | | | | | | | | | | | | | |
| --- | --- | --- | --- | --- | --- | --- | --- | --- | --- | --- | --- | --- | --- | --- | --- | --- | --- | --- | --- |
| **Themes** | **Dimensions** | **Questionnaires** | | | | | | | | | | | | | | | | | |
|  |  | **Beck Anxiety Inventory [74]** | **Beck Depression Inventory [75-76]** | **Behavioral Risk Factor Surveillance System [34]** | **Brief Symptom Inventory [77]** | **Center for Epidemiologic Studies- Depression [78]** | **Composite International Diagnostic Interview [79-80]** | **Depression, Anxiety and Stress Scale [81]** | **Difficulties in Emotion Regulation Scale [82]** | **General Health Questionnaire [83-84]** | **General Health Questionnaire [85-86]** | **General Health Questionnaire [83, 87]** | **General Stress Scale [88]** | **General Symptom Index [89]** | **General Social Survey Questionnaire [37]** | **Geriatric Depression Scale [90]** | **Goldberg Anxiety and Depression Scales [91]** | **Grief Scale [92]** | **Hospital Anxiety and Depression Scale [93]** |
| **Medication use** |  |  |  |  |  |  |  |  |  |  |  |  |  |  |  |  |  |  |  |
|  | **Tranquillizers, sedatives, and hypnotics** |  |  |  |  |  |  |  |  |  |  |  |  |  |  |  |  |  |  |
|  | **Antidepressants** |  |  |  |  |  |  |  |  |  |  |  |  |  |  |  |  |  |  |
| **Mental health** | **Mental health** |  |  |  |  |  |  |  |  |  |  |  |  |  |  |  |  |  |  |
|  | **Negative affect** |  |  |  |  |  |  |  |  |  |  |  |  |  |  |  |  |  |  |
|  | **Positive affect** |  |  |  |  | Good, hopeful, happy, enjoy |  |  |  |  |  |  |  |  |  |  |  |  |  |
|  | **Poor mental health days** |  |  | Number of poor mental health days in past month |  |  |  |  |  |  |  |  |  |  |  |  |  |  |  |
|  | **Screening and lifetime review** |  |  |  |  |  | Presence or absence of lifetime diagnoses of affective disorders, anxiety disorders and substance use disorders assessed using International Classification of Diseases (ICD-10) criteria and the presence or absence of symptoms in the past 12 months |  |  |  |  |  |  |  |  |  |  |  |  |
| **Emotional health** | **Emotional health** |  |  |  |  |  |  |  |  |  |  |  |  |  | Feeling tired, worried, or anxious, overwhelmed, lonely or isolated, short-tempered, or irritable, resentful, depressed, loss of appetite, disturbed sleep |  |  |  |  |
|  | **Lack of emotional awareness** |  |  |  |  |  |  |  | Tendency to attend to and acknowledge emotions |  |  |  |  |  |  |  |  |  |  |
|  | **Lack of emotional clarity** |  |  |  |  |  |  |  | Extent to which individuals know (and are clear about) the emotions they are experiencing |  |  |  |  |  |  |  |  |  |  |
|  | **Nonacceptance of emotional responses** |  |  |  |  |  |  |  | Tendency to have negative secondary emotional responses to one's negative emotions, or nonaccepting reactions to one's distress |  |  |  |  |  |  |  |  |  |  |
|  | **Confusion-bewilderment** |  |  |  |  |  |  |  |  |  |  |  |  |  |  |  |  |  |  |
|  | **Vigor-activity** |  |  |  |  |  |  |  |  |  |  |  |  |  |  |  |  |  |  |
| **Perceived stress** | **Perceived stress** |  |  |  |  |  |  | Includes the core symptoms of stress, such as difficulty relaxing, nervous arousal, easily upset/agitated, irritable/ over-reactive and impatient |  |  |  |  |  |  |  |  |  |  |  |
|  | **Stress: Economic** |  |  |  |  |  |  |  |  |  |  |  | Stress related to economic matters |  |  |  |  |  |  |
|  | **Stress: Health** |  |  |  |  |  |  |  |  |  |  |  | Stress related to health |  |  |  |  |  |  |
|  | **Stress: Lifestyle** |  |  |  |  |  |  |  |  |  |  |  | Stress related to lifestyle |  |  |  |  |  |  |
|  | **Stress: Relationships** |  |  |  |  |  |  |  |  |  |  |  | Stress related to relationships |  |  |  |  |  |  |
|  | **Stress: Social issues** |  |  |  |  |  |  |  |  |  |  |  | Stress related to social issues |  |  |  |  |  |  |
| **Anxiety** | **Anxiety** |  |  |  | Includes nervousness or shakiness inside, feelings tense or keyed up, suddenly scared or no reason, spells of terror or panic, feeling so restless you couldn’t sit still and feeling fearful |  |  | Includes the core symptoms of anxiety, such as autonomic arousal, skeletal musculature effects, situational anxiety, and subjective experience of anxious affect |  |  | Encompasses losing sleep over worry, having difficulty staying asleep, feeling constantly under strain, getting edgy and bad-tempered, getting scared or panicky for no good reason, everything getting on top of you and feeling nervous and strung-up all the time | Anxiety, worry and tension  e.g., "Felt constantly under strain?" "Found everything getting on top of you?" Been feeling nervous and strung-up all the time?" "Been feeling unhappy and depressed? "Been taking things hard? "Felt you couldn't overcome your difficulties?" "Lost much sleep over worry?" "Been losing confidence in yourself?" "Found at times you couldn't do anything because your nerves were too bad?" "Been thinking of yourself as a worthless person?" |  | Anxiety symptom |  |  | Feeling keyed up, on edge, worrying a lot, being irritable, difficulty relaxing, sleeping poorly, headaches and neck aches, trembling, tingling, dizzy spells, sweating, diarrhea, worried about health and difficulty falling asleep |  | Psychic manifestations of anxiety neurosis |
|  | **Anxiety: phobic** |  |  |  |  |  |  |  |  |  |  |  |  | Phobic anxiety symptom |  |  |  |  |  |
|  | **Anxiety: somatic affective** | Numbness or tingling, feeling hot, wobbliness in legs, heart pounding or racing, unsteady, terrified, hands trembling, shaky, scared, faint, face flushed, and sweating (not due to heat) |  |  |  |  |  |  |  |  |  |  |  |  |  |  |  |  |  |
|  | **Anxiety: subjective and panic-related** | Unable to relax, dizzy or lightheaded, terrified, nervous, feelings of choking, fear of losing control, difficulty of breathing, fear of dying, and indigestion or discomfort in abdomen |  |  |  |  |  |  |  |  |  |  |  |  |  |  |  |  |  |
| **Burnout** |  |  |  |  |  |  |  |  |  |  |  |  |  |  |  |  |  |  |  |
|  | **Burnout: depersonalization** |  |  |  |  |  |  |  |  |  |  |  |  |  |  |  |  |  |  |
|  | **Burnout: emotional exhaustion** |  |  |  |  |  |  |  |  |  |  |  |  |  |  |  |  |  |  |
|  | **Burnout: personal accomplishment** |  |  |  |  |  |  |  |  |  |  |  |  |  |  |  |  |  |  |
| **Depression** | **Depression** |  |  |  | Includes feeling no interest in things, feeling lonely, feeling blue, feelings of worthlessness, feeling hopeless about the future and thoughts of ending your life | Blues, depressed, lonely, cry sad |  | Includes the core symptoms of depression, such as dysphoria, hopelessness, devaluation of life, self-deprecation, devaluation of life, self-deprecation, lack of interest/ involvement, anhedonia, and inertia |  |  | Encompasses the respondent thinking they are worthless, feeling that life isn't worth living, considering committing suicide, finding at times that they are not able to do anything due to their nerves being so bad, found themselves wishing they were dead and away from it all and finding the idea of taking their own life keeps coming up in their mind | Depression, hopelessness  e.g., "Felt that life isn't worth living?" "Been thinking of yourself as a worthless  person?" "Felt that life is entirely hopeless?" "Been feeling hopeful about your own future?" "Found at times you couldn't do anything  because your nerves were too bad?" "Been losing confidence in yourself?" |  | Depression symptom |  | Includes satisfaction with life, dropping activities and interests, being bored, being afraid that something bad may happen, feeling helpless, preferring to stay home, having problems with memory | Includes having low energy, loss of interests, losing confidence in self, feeling hopeless, having difficulty concentrating, losing weight (i.e., due to poor appetite), waking up early, feeling slowed up and tending to feel worse in the mornings |  | Anhedonic state |
|  | **Depression: cognitive affective** |  | Pessimism and worthlessness |  |  |  |  |  |  |  |  |  |  |  |  |  |  |  |  |
|  | **Depression: somatic affective** |  | Fatigue and loss of energy |  |  |  |  |  |  |  |  |  |  |  |  |  |  |  |  |
|  | **Depression: duration of depressive symptoms** |  |  |  |  |  |  |  |  |  |  |  |  |  |  |  |  |  |  |
|  | **Depression: persistence of depressive symptoms** |  |  |  |  |  |  |  |  |  |  |  |  |  |  |  |  |  |  |
| **Post-traumatic stress disorder** |  |  |  |  |  |  |  |  |  |  |  |  |  |  |  |  |  |  |  |
|  | **Post-traumatic stress disorder: avoidance** |  |  |  |  |  |  |  |  |  |  |  |  |  |  |  |  |  |  |
|  | **Post-traumatic stress disorder: hyperarousal** |  |  |  |  |  |  |  |  |  |  |  |  |  |  |  |  |  |  |
|  | **Post-traumatic stress disorder: intrusion** |  |  |  |  |  |  |  |  |  |  |  |  |  |  |  |  |  |  |
| **Psychological distress** | **Psychological distress** |  |  |  |  |  |  |  |  | Includes ability to concentrate, losing sleep, having purpose, capable of making decisions, under stress, cannot overcome difficulties, enjoying normal activities, facing up to problems, feeling unhappy and depressed, losing confidence, thinking of self as worthless and feeling reasonably happy |  |  |  |  |  |  |  |  |  |
| **Environmental mastery** | **Environmental mastery** |  |  |  |  |  |  |  |  |  |  |  |  |  |  |  |  |  |  |
|  | **Relationships with others** |  |  |  |  |  |  |  |  |  |  |  |  |  |  |  |  |  |  |
|  | **Autonomy** |  |  |  |  |  |  |  |  |  |  |  |  |  |  |  |  |  |  |
|  | **Difficulties engaging in goal-direct behavior** |  |  |  |  |  |  |  | Difficulties concentrating and accomplishing tasks when experiencing negative emotions |  |  |  |  |  |  |  |  |  |  |
|  | **Social dysfunction** |  |  |  |  |  |  |  |  |  | Managing to keep themselves busy and occupied, taking longer over normal tasks, on the whole feeling like they are doing things well, being satisfied with the way they have carried out a task, feeling like they are playing a useful part in things, feeling capable of making decisions about things and being able to enjoy normal day-to-day activities | Social dysfunction  e.g., "Spent much time chatting with people?" "Been finding it easy to get on with other people?" "Been getting out of the house as much as usual?" "Felt that you are playing a useful part in  things?" |  |  |  |  |  |  |  |
| **Memory failures** |  |  |  |  |  |  |  |  |  |  |  |  |  |  |  |  |  |  |  |
|  | **Memory failures: prospective** |  |  |  |  |  |  |  |  |  |  |  |  |  |  |  |  |  |  |
|  | **Memory failures: retrospective** |  |  |  |  |  |  |  |  |  |  |  |  |  |  |  |  |  |  |
|  | **Impulse control difficulties** |  |  |  |  |  |  |  | Difficulties remaining in control of one's behavior when experiencing negative emotions |  |  |  |  |  |  |  |  |  |  |
|  | **Anger: control** |  |  |  |  |  |  |  |  |  |  |  |  |  |  |  |  |  |  |
|  | **Anger: external** |  |  |  |  |  |  |  |  |  |  |  |  |  |  |  |  |  |  |
|  | **Anger: hostility** |  |  |  |  |  |  |  |  |  |  |  |  |  |  |  |  |  |  |
|  | **Anger: internal** |  |  |  |  |  |  |  |  |  |  |  |  |  |  |  |  |  |  |
|  | **Anger: trait** |  |  |  |  |  |  |  |  |  |  |  |  |  |  |  |  |  |  |
| **Somatization** | **Somatization** |  |  |  | Includes faintness or dizziness, pains in heart or chest, nausea or upset stomach, trouble getting your breath, numbness or tingling in parts of your body and feeling weak in parts of your body | Bothered, appetite, effort, sleep, get going |  |  |  |  | Feeling perfectly well and in good health, feeling in need of a good tonic, feeling run down and out of sorts, feeling ill, getting pains in head, feelings of tightness or pressure in head and having hot or cold spells |  |  | Somatization symptom |  |  |  |  |  |
|  | **Fatigue** |  |  |  |  |  |  |  |  |  |  |  |  |  |  |  |  |  |  |
| **Feelings of incompetence** | **Feelings of incompetence** |  |  |  |  |  |  |  |  |  |  | Feelings of incompetence, low self-esteem  e.g.,  "Been satisfied with the way you've carried out your task?" "Been managing to keep yourself busy and occupied?" "Been managing as well as most people would in your shoes?" "Felt that you are playing a useful part in  things?" "Been able to concentrate on whatever  you're doing?" "Been getting out of the house as much as usual?" |  |  |  |  |  |  |  |
| **General worry** | **General worry** |  |  |  |  |  |  |  |  |  |  |  |  |  |  |  |  |  |  |
| **Grief** |  |  |  |  |  |  |  |  |  |  |  |  |  |  |  |  |  |  |  |
|  | **Grief: current** |  |  |  |  |  |  |  |  |  |  |  |  |  |  |  |  | Current feelings of grief associated with the mental illness of a loved one |  |
|  | **Grief: initial** |  |  |  |  |  |  |  |  |  |  |  |  |  |  |  |  |  |  |
| **Hostility** | **Hostility** |  |  |  |  |  |  |  |  |  |  |  |  | Hostility symptom |  |  |  |  |  |
| **Interpersonal** | **Interpersonal** |  |  |  |  | Unfriendly, dislike |  |  |  |  |  |  |  |  |  |  |  |  |  |
|  | **Interpersonal sensitivity** |  |  |  |  |  |  |  |  |  |  |  |  | Interpersonal sensitivity symptom |  |  |  |  |  |
| **Difficulty in coping** | **Difficulty in coping** |  |  |  |  |  |  |  |  |  |  | Difficulty in coping, dispirited e.g., "Been finding life a struggle all the time?" "Been able to enjoy your normal day to-day activities?" "Been feeling reasonably happy, all things considered?" "Been getting scared or panicky for no good reason?" "Felt capable of making decisions about things?" "Been feeling that life is entirely hopeless?" "Been having restless, disturbed nights?" |  |  |  |  |  |  |  |
| **Limited access to emotion regulation strategies** | **Limited access to emotion regulation strategies** |  |  |  |  |  |  |  | Belief that there is little that can be done to regulate emotions effectively, once an individual is upset |  |  |  |  |  |  |  |  |  |  |
| **Obsessive-compulsive** | **Obsessive-compulsive** |  |  |  |  |  |  |  |  |  |  |  |  | Obsessive-compulsive symptom |  |  |  |  |  |
| **Paranoid ideation** | **Paranoid ideation** |  |  |  |  |  |  |  |  |  |  |  |  | Paranoid ideation symptom dimension |  |  |  |  |  |
| **Personal growth** | **Personal growth** |  |  |  |  |  |  |  |  |  |  |  |  |  |  |  |  |  |  |
| **Psychoticism** | **Psychoticism** |  |  |  |  |  |  |  |  |  |  |  |  | Psychoticism symptom |  |  |  |  |  |
| **Purpose in life** | **Purpose in life** |  |  |  |  |  |  |  |  |  |  |  |  |  |  |  |  |  |  |
| **Self-acceptance** | **Self-acceptance** |  |  |  |  |  |  |  |  |  |  |  |  |  |  |  |  |  |  |

# Meta-summary of mental health (2/2)

| **Meta-summary of mental health (2/2)** | | | | | | | | | | | | | | | | | | |
| --- | --- | --- | --- | --- | --- | --- | --- | --- | --- | --- | --- | --- | --- | --- | --- | --- | --- | --- |
| **Themes** | **Dimensions** | **Questionnaires** | | | | | | | | | | | | | | | | |
|  |  | **Impact of Event Scale-Revised [94]** | **Kessler Psychological Distress Scale-10 [95]** | **Maslach Burnout Inventory- Human Services Survey [96]** | **Mental Health Inventory-5 [97]** | **Mental Illness Version of the Texas Inventory of Grief [98]** | **Non-Specific Psychological Distress and Positive Emotions Scale [99]** | **Penn State Worry Questionnaire [100]** | **Perceived Stress Scale [101]** | **Profile of Mood States [102]** | **Prospective and Retrospective Memory Questionnaire [103]** | **Psychological Wellbeing (PWB) Scale [104]** | **Texas Inventory of Grief-Early Intervention [39]** | **State-Trait Anger Scale [105]** | **Symptom Check List Revised [106-107]** | **Techniker Krankenkasse [108]** | **Warwick-Edinburgh Mental Wellbeing Scale [109]** | **WHO World Health Survey [110]** |
| **Medication use** |  |  |  |  |  |  |  |  |  |  |  |  |  |  |  |  |  |  |
|  | **Tranquillizers, sedatives, and hypnotics** |  |  |  |  |  |  |  |  |  |  |  |  |  |  | If tranquillizers, sedatives, and hypnotics (ATC: N05B, N05C)  were prescribed |  |  |
|  | **Antidepressants** |  |  |  |  |  |  |  |  |  |  |  |  |  |  | If antidepressants (ATC: N06A) were prescribed |  |  |
| **Mental health** | **Mental health** |  |  |  |  |  |  |  |  |  |  |  |  |  |  |  | Individual's state of mental well-being (i.e., thoughts and feelings) in the previous two weeks |  |
|  | **Negative affect** |  |  |  |  |  | Anxiety and depression |  |  |  |  |  |  |  |  |  |  |  |
|  | **Positive affect** |  |  |  | NR |  | Enthusiasm, alertness, and vitality |  |  |  |  |  |  |  |  |  |  |  |
|  | **Poor mental health days** |  |  |  |  |  |  |  |  |  |  |  |  |  |  |  |  |  |
|  | **Screening and lifetime review** |  |  |  |  |  |  |  |  |  |  |  |  |  |  |  |  |  |
| **Emotional health** | **Emotional health** |  |  |  | Behavioral/ emotional control |  |  |  |  |  |  |  |  |  |  |  |  |  |
|  | **Lack of emotional awareness** |  |  |  |  |  |  |  |  |  |  |  |  |  |  |  |  |  |
|  | **Lack of emotional clarity** |  |  |  |  |  |  |  |  |  |  |  |  |  |  |  |  |  |
|  | **Nonacceptance of emotional responses** |  |  |  |  |  |  |  |  |  |  |  |  |  |  |  |  |  |
|  | **Confusion-bewilderment** |  |  |  |  |  |  |  |  | Feeling state characterized by bewilderment and uncertainty, associated with  a general failure to control attention and emotions |  |  |  |  |  |  |  |  |
|  | **Vigor-activity** |  |  |  |  |  |  |  |  | Feelings of excitement, alertness, and physical energy |  |  |  |  |  |  |  |  |
| **Perceived stress** | **Perceived stress** |  |  |  |  |  |  |  | Perceived stress specifically related to caring for the care recipient |  |  |  |  |  |  |  |  |  |
|  | **Stress: Economic** |  |  |  |  |  |  |  |  |  |  |  |  |  |  |  |  |  |
|  | **Stress: Health** |  |  |  |  |  |  |  |  |  |  |  |  |  |  |  |  |  |
|  | **Stress: Lifestyle** |  |  |  |  |  |  |  |  |  |  |  |  |  |  |  |  |  |
|  | **Stress: Relationships** |  |  |  |  |  |  |  |  |  |  |  |  |  |  |  |  |  |
|  | **Stress: Social issues** |  |  |  |  |  |  |  |  |  |  |  |  |  |  |  |  |  |
| **Anxiety** | **Anxiety** |  |  |  | NR |  |  |  |  | Feelings such as nervousness, apprehension, worry, and anxiety |  |  |  |  | Primary symptom- anxiety |  |  |  |
|  | **Anxiety: phobic** |  |  |  |  |  |  |  |  |  |  |  |  |  | Primary symptom- phobic anxiety |  |  |  |
|  | **Anxiety: somatic affective** |  |  |  |  |  |  |  |  |  |  |  |  |  |  |  |  |  |
|  | **Anxiety: subjective and panic-related** |  |  |  |  |  |  |  |  |  |  |  |  |  |  |  |  |  |
| **Burnout** |  |  |  |  |  |  |  |  |  |  |  |  |  |  |  |  |  |  |
|  | **Burnout: depersonalization** |  |  | Negative attitudes and responses to care recipient |  |  |  |  |  |  |  |  |  |  |  |  |  |  |
|  | **Burnout: emotional exhaustion** |  |  | Feeling drained and overextended in work |  |  |  |  |  |  |  |  |  |  |  |  |  |  |
|  | **Burnout: personal accomplishment** |  |  | Feeling incompetent, inadequate, and ineffective in role |  |  |  |  |  |  |  |  |  |  |  |  |  |  |
| **Depression** | **Depression** |  |  |  | NR |  |  |  |  | Negative self-schema characterized by themes such as hopelessness, personal deficiency, worthlessness, and self-blame |  |  |  |  | Primary symptom- depression |  |  |  |
|  | **Depression: cognitive affective** |  |  |  |  |  |  |  |  |  |  |  |  |  |  |  |  |  |
|  | **Depression: somatic affective** |  |  |  |  |  |  |  |  |  |  |  |  |  |  |  |  |  |
|  | **Depression: duration of depressive symptoms** |  |  |  |  |  |  |  |  |  |  |  |  |  |  |  |  | NR |
|  | **Depression: persistence of depressive symptoms** |  |  |  |  |  |  |  |  |  |  |  |  |  |  |  |  | NR |
| **Post-traumatic stress disorder** |  |  |  |  |  |  |  |  |  |  |  |  |  |  |  |  |  |  |
|  | **Post-traumatic stress disorder: avoidance** | Effortful avoidance of situations that serve as reminders of the trauma |  |  |  |  |  |  |  |  |  |  |  |  |  |  |  |  |
|  | **Post-traumatic stress disorder: hyperarousal** | Feeling irritable and angry, jumpy, easily startled, having trouble falling asleep, having trouble concentrating, physical reactions and being watchful and on guard |  |  |  |  |  |  |  |  |  |  |  |  |  |  |  |  |
|  | **Post-traumatic stress disorder: intrusion** | Repeated thoughts about the trauma |  |  |  |  |  |  |  |  |  |  |  |  |  |  |  |  |
| **Psychological distress** | **Psychological distress** |  | Includes depressed mood, motor agitation, fatigue, worthless guilt, and anxiety |  |  |  |  |  |  |  |  |  |  |  |  |  |  |  |
| **Environmental mastery** | **Environmental mastery** |  |  |  |  |  |  |  |  |  |  | Sense of mastery and competence in managing the environment; controls complex array of external activities; makes effective use of surrounding opportunities; able to choose or create contexts suitable to personal needs and value |  |  |  |  |  |  |
|  | **Relationships with others** |  |  |  |  |  |  |  |  |  |  | Has warm, satisfying, trusting relationships with others; is concerned about the welfare of others; capable of strong empathy, affection, and intimacy; understands give and take of human relationships. |  |  |  |  |  |  |
|  | **Autonomy** |  |  |  |  |  |  |  |  |  |  | Is self-determining and independent; able to resist social pressures to think and act in certain ways; regulates behavior from within; evaluates self by personal standards |  |  |  |  |  |  |
|  | **Difficulties engaging in goal-direct behavior** |  |  |  |  |  |  |  |  |  |  |  |  |  |  |  |  |  |
|  | **Social dysfunction** |  |  |  |  |  |  |  |  |  |  |  |  |  |  |  |  |  |
| **Memory failures** |  |  |  |  |  |  |  |  |  |  |  |  |  |  |  |  |  |  |
|  | **Memory failures: prospective** |  |  |  |  |  |  |  |  |  | e.g., do you forget appointments if you are not prompted by someone else or by a reminder such as a calendar or diary? |  |  |  |  |  |  |  |
|  | **Memory failures: retrospective** |  |  |  |  |  |  |  |  |  | e.g., do you fail to do something you were supposed to do a few minutes later even though it’s there in front of you, like take a pill or turn off the kettle? |  |  |  |  |  |  |  |
| **Impulse control difficulties** | **Impulse control difficulties** |  |  |  |  |  |  |  |  |  |  |  |  |  |  |  |  |  |
|  | **Anger: control** |  |  |  |  |  |  |  |  |  |  |  |  | NR |  |  |  |  |
|  | **Anger: external** |  |  |  |  |  |  |  |  |  |  |  |  | NR |  |  |  |  |
|  | **Anger: hostility** |  |  |  |  |  |  |  |  | Feelings that vary in intensity from mild annoyance or aggravation to fury and rage,  and is associated with arousal of the autonomic nervous system |  |  |  |  |  |  |  |  |
|  | **Anger: internal** |  |  |  |  |  |  |  |  |  |  |  |  | NR |  |  |  |  |
|  | **Anger: trait** |  |  |  |  |  |  |  |  |  |  |  |  | NR |  |  |  |  |
| **Somatization** | **Somatization** |  |  |  |  |  |  |  |  |  |  |  |  |  | Primary symptom-somatization |  |  |  |
|  | **Fatigue** |  |  |  |  |  |  |  |  | Feelings of mental and physical tiredness |  |  |  |  |  |  |  |  |
| **Feelings of incompetence** | **Feelings of incompetence** |  |  |  |  |  |  |  |  |  |  |  |  |  |  |  |  |  |
| **General worry** | **General worry** |  |  |  |  |  |  | Concerns about physical safety and health, concerns about social evaluation, beliefs in worry as a positive coping strategy, depression, and lack of optimism about life, and concerns about future success in relationships |  |  |  |  |  |  |  |  |  |  |
| **Grief** |  |  |  |  |  |  |  |  |  |  |  |  |  |  |  |  |  |  |
|  | **Grief: current** |  |  |  |  | Current grief in response to their relative's mental illness |  |  |  |  |  |  | Present feelings about the relative's perceived loss |  |  |  |  |  |
|  | **Grief: initial** |  |  |  |  | Initial grief in response to their relative's mental illness |  |  |  |  |  |  | Relative's behavior when they first noticed that their loved one was experiencing mental health difficulties |  |  |  |  |  |
| **Hostility** | **Hostility** |  |  |  |  |  |  |  |  |  |  |  |  |  | Primary symptom- hostility |  |  |  |
| **Interpersonal** | **Interpersonal** |  |  |  |  |  |  |  |  |  |  |  |  |  |  |  |  |  |
|  | **Interpersonal sensitivity** |  |  |  |  |  |  |  |  |  |  |  |  |  | Primary symptom- interpersonal sensitivity |  |  |  |
| **Difficulty in coping** | **Difficulty in coping** |  |  |  |  |  |  |  |  |  |  |  |  |  |  |  |  |  |
| **Limited access to emotion regulation strategies** | **Limited access to emotion regulation strategies** |  |  |  |  |  |  |  |  |  |  |  |  |  |  |  |  |  |
| **Obsessive-compulsive** | **Obsessive-compulsive** |  |  |  |  |  |  |  |  |  |  |  |  |  | Primary symptom- obsessive-compulsive |  |  |  |
| **Paranoid ideation** | **Paranoid ideation** |  |  |  |  |  |  |  |  |  |  |  |  |  | Primary symptom- paranoid ideation |  |  |  |
| **Personal growth** | **Personal growth** |  |  |  |  |  |  |  |  |  |  | Has a feeling of continued development; sees self as growing and expanding; is open to new experiences; has sense of realizing his or her potential; sees improvement in self and behavior over time; is changing in ways that reflect more self-knowledge and effectiveness |  |  |  |  |  |  |
| **Psychoticism** | **Psychoticism** |  |  |  |  |  |  |  |  |  |  |  |  |  | Primary symptom- psychoticism |  |  |  |
| **Purpose in life** | **Purpose in life** |  |  |  |  |  |  |  |  |  |  | Has goals in life and a sense of directedness; feels there is meaning to present and past life; holds beliefs that give life purpose; has aims and objectives for living |  |  |  |  |  |  |
| **Self-acceptance** | **Self-acceptance** |  |  |  |  |  |  |  |  |  |  | Possesses a positive attitude toward the self; acknowledges and accepts multiple aspects of self, including good and bad qualities; feels positive about past life |  |  |  |  |  |  |

# Meta-summary of the overall caregiving situation

| **Meta-summary of the overall caregiving situation** | | | | | | | | | | |
| --- | --- | --- | --- | --- | --- | --- | --- | --- | --- | --- |
| **Themes** | **Dimensions** | **Questionnaires** | | | | | | | | |
|  |  | **Caregivers’ and Users’ Expectations of Services- Caregiver version [111]** | **Experience of Caregiving Inventory [112]** | **General Self-Efficacy Scale [113]** | **General Social Survey questionnaire [37]** | **Nursing Awareness [114]** | **Perceptions of Prevalence of Aggression Scale [115]** | **Self-developed survey by Labrum & Solomon [116]** | **Self-developed survey by the Private Mental Health Consumer Caregiver Network [117]** | **Well Sibling Guilt Index (WSIGI) of the Well Sibling Guilt Questionnaire [118]** |
| **Caregiver’s appraisal of their caregiving** |  |  |  |  |  |  |  |  |  |  |
|  | **Awareness of care** |  |  |  |  | NR |  |  |  |  |
|  | **Self-efficacy** |  |  | Individuals’ belief in their own ability to succeed in specific situations. Individuals with high self-efficacy are those who believe they can manage well in difficult situations and do not avoid them |  |  |  |  |  |  |
| **Caregiver’s appraisal of negative aspects of caregiving situation** |  |  |  |  |  |  |  |  |  |  |
|  | **Burden of caring** | NR |  |  |  |  |  |  |  |  |
|  | **Caregiving stress** |  |  |  | How stressful caregiver perceives caregiving |  |  |  |  |  |
|  | **Dependency** |  | Encompasses being unable to do things that the caregiver wants to do, the care recipient’s dependence on their caregiver and unable to leave the care recipient home alone |  |  |  |  |  |  |  |
|  | **Interaction guilt** |  |  |  |  |  |  |  |  | NR |
|  | **Problems with services** |  | Encompasses dealing with psychiatrists and other mental health professionals and difficulties getting knowledge about illness |  |  |  |  |  |  |  |
|  | **Safety fears** |  |  |  |  |  |  | Encompasses the fear that the care recipient will be hurt by someone else, destroys property, hurting themselves, hurting themselves or someone else |  |  |
|  | **Stigma** |  | Encompasses covering up care recipient’s illness and feeling unable to have visitors at home |  |  |  |  |  |  |  |
| **Caregiver’s appraisal of positive aspects of caregiving situation** |  |  |  |  |  |  |  |  |  |  |
|  | **Caregivers feel free to lead their own lives/ exercise choice about their role as caregiver** | NR |  |  |  |  |  |  |  |  |
|  | **Good aspects of relationship** |  | e.g., “I have contributed to his wellbeing,” “He makes a valuable contribution to the household” “He has shown strengths coping with his illness” “I share some of his interests” “I feel useful in my relationship with him” |  |  |  |  |  |  |  |
|  | **Positive personal experiences** |  | e.g., “I have learnt more about myself,” “I have contributed to others’ understanding of the illness,” “I have become more understanding of others with problems,” “I have discovered strengths in myself” |  |  |  |  |  |  |  |
| **Caregiver involvement in care recipient’s formal care** |  |  |  |  |  |  |  |  |  |  |
|  | **Care recipient assessment process** |  |  |  |  |  |  |  | NR |  |
|  | **Care recipient treatment** |  |  |  |  |  |  |  | NR |  |
|  | **Care recipient ongoing management** |  |  |  |  |  |  |  | NR |  |
|  | **Decisions about the person you care for** |  |  |  |  |  |  |  | NR |  |
|  | **Care recipient discharge planning** |  |  |  |  |  |  |  | NR |  |
| **Caregiver support** |  |  |  |  |  |  |  |  |  |  |
|  | **Quality of help, provided to caregivers by services providers and extent to which service providers involve the caregiver in planning of treatment and care** | NR |  |  |  |  |  |  |  |  |
|  | **Requesting help for the impact of caring on the caregiver’s own life** | NR |  |  |  |  |  |  |  |  |
|  | **Requesting help to improve the quality of help provided to caregivers by service providers and extent to which the service providers involve the caregiver in planning of treatment and care** | NR |  |  |  |  |  |  |  |  |
| **Caregiving tasks** |  |  |  |  |  |  |  |  |  |  |
|  | **Need to back up** |  | Encompasses difficulties looking after money, having to support the care recipient, the effect of caregiving on the caregiver’s finances and setting up accommodation for the care recipient |  |  |  |  |  |  |  |
| **Caregiver’s appraisal of the care recipient** |  |  |  |  |  |  |  |  |  |  |
|  | **Auto-aggression** |  |  |  |  |  | Includes self-afflicted violence against the care recipient, suicide |  |  |  |
|  | **Difficult behaviors** |  | Encompasses the care recipient behaving in a moody, unpredictable, irritable, inconsiderate, or reckless manner |  |  |  |  |  |  |  |
|  | **Forms of verbal and threatening aggression** |  |  |  |  |  | Includes non-threatening verbal aggression, threatening verbal aggression, passive aggressive behavior, humiliating aggressive behavior |  |  |  |
|  | **Loss** |  | Encompasses lost opportunities of the care recipient and the risk of the care recipient committing suicide or self-harm |  |  |  |  |  |  |  |
|  | **Negative symptoms** |  | Encompasses the care recipient behaving in a withdrawn uncommunicative and uninterested manner and feeling unable to have visitors at home |  |  |  |  |  |  |  |
|  | **Physical aggression** |  |  |  |  |  | Includes threatening physical aggression |  |  |  |
|  | **Sexual aggression** |  |  |  |  |  | Includes sexual intimidation, sexual assault, rape |  |  |  |
| **Effects on family** | **Effects on family** |  | Encompasses family members not understanding caregiver’s situation, family members not understanding the illness of the care recipient and others leaving home because of the illness |  |  |  |  |  |  |  |

# Meta-summary of overall health

| **Meta-summary of overall health** | | | | | | | | | | |
| --- | --- | --- | --- | --- | --- | --- | --- | --- | --- | --- |
| **Themes** | **Dimensions** | **Questionnaires** | | | | | | | | |
|  |  | **1992-1993 Family Impact Study [92, 119]** | **Behavioral Risk Factor Surveillance System [34]** | **Caregiver Well-Being and Support [120]** | **Cornell Medical Index [121]** | **Insurance-Medicine-All-Sweden (IMAS) study [46]** | **Medical Outcomes Survey-Short Form [122-124]** | **Self-developed questionnaire by Ali et al. [125]** | **Self-developed questionnaire by Csoboth et al. [126]** | **Wisconsin Longitudinal Study (WLS) Survey [127]** |
| **Caregiver day-to-day life** | **Caregiver day-to-day life** |  |  | NR |  |  |  |  |  |  |
| **Caregiving situation** |  |  |  |  |  |  |  |  |  |  |
|  | **Caregiver safety** |  |  | NR |  |  |  |  |  |  |
|  | **Relationship with care recipient** |  |  | NR |  |  |  |  |  |  |
|  | **Role as caregiver** |  |  | NR |  |  |  |  |  |  |
|  | **Safety of the care recipient** |  |  | NR |  |  |  |  |  |  |
| **Characteristics of caregiver** |  |  |  |  |  |  |  |  |  |  |
|  | **Inadequacy** |  |  |  | Includes sweating and trembling during examinations, nervous when approached by a superior, doing things slowly to avoid making mistake, afraid of strangers, scared to be alone, hard to make up mind, clumsy, not able to eat anywhere except in own home |  |  |  |  |  |
|  | **Sensitivity** |  |  |  | Includes extreme shyness and sensitivity, feelings easily hurt, being misunderstood |  |  |  |  |  |
|  | **Tension** |  |  |  | Breaking out in cold sweat, having recurrent frightening thoughts, scared at sudden movements or noises at night, jumping or shaking due to sudden movements |  |  |  |  |  |
| **Financial situation** | **Financial situation** |  |  | NR |  |  |  |  |  |  |
| **Functioning** |  |  |  |  |  |  |  |  |  |  |
|  | **Physical functioning** |  |  |  |  |  | Limitations in moderate physical activities and in climbing several flights of stairs |  |  |  |
|  | **Social functioning** |  |  |  |  |  | Interference of physical health or emotional problems with social activities |  |  |  |
| **General health** | **General health** |  | General health status |  |  |  | Perceived general health condition | Self-rated health |  |  |
|  | **Disability status** |  |  |  |  |  |  |  | Includes long- and short-term disability status |  |
|  | **Lifestyle measures** |  |  |  | NR |  |  |  |  |  |
|  | **Medication use** |  |  |  |  | NR |  |  |  |  |
|  | **Mortality** |  |  |  |  | NR |  |  |  |  |
|  | **Vitality** |  |  |  | NR |  | Includes having a lot of energy and fatigability |  |  |  |
| **Mental health** | **Mental health** |  |  |  |  |  | Includes feeling calm, peaceful, downhearted, and blue |  |  |  |
|  | **Anger** |  |  |  | Includes being on guard, being impulsive, overreacting, violent rages |  |  |  |  |  |
|  | **Conditions: Anxiety** |  |  |  | Includes nerves, occurrence of a nervous breakdown, having been a patient in a mental hospital |  |  |  |  |  |
|  | **Conditions: Depression** |  |  |  | Includes feeling alone and sad at a party, feeling unhappy, depressed, and hopeless, suicidal thoughts, crying often |  |  |  |  |  |
|  | **Drug use** |  |  |  |  | NR |  | Use of non-prescription drugs in the last 4 months |  |  |
|  | **General mood** |  |  |  |  |  |  | How easily respondent has been irritated in the last 4 months |  |  |
|  | **Role-emotional problems** |  |  |  |  |  | Respondent is not careful in work or activities due to emotional problems and accomplished less due to emotional problems |  |  |  |
| **Physical health** | **Physical health** |  |  | NR |  |  |  |  |  |  |
|  | **Bodily pain** |  |  |  |  |  | Pain interference with work inside or outside the home |  |  |  |
|  | **Conditions: Cardiovascular system** |  |  |  | Blood pressure, pains in heart or chest, arrhythmia, difficulties breathing, swollen ankles, cold hands or feet in warm weather, frequent cramps in legs, heart conditions |  |  |  |  |  |
|  | **Conditions: Digestive tract** |  |  |  | Dental problems, poor appetite, snacking between meals, bloating, suffering from an upset stomach, indigestion, belching after eating, stomach ulcers, diarrhea, gall bladder trouble, rectal hemorrhoids, intestinal worms, constipation |  |  |  |  |  |
|  | **Conditions: Eyes and ears** |  |  |  | Needing to wear glasses, bad pains in eyes, being hard of hearing, tinnitus, having running eyes or ears, blacked out eyesight |  |  |  |  |  |
|  | **Conditions: Genitourinary** |  |  |  | Issues with genitalia, hernia, issues urinating, kidney or bladder disease |  |  |  |  |  |
|  | **Conditions: Miscellaneous** |  |  |  | Scarlet fever, rheumatic fever, malaria, severe anemia, venereal diseases, diabetes, goiter, varicose veins, overweight, underweight, serious, and small operations, and injuries |  |  |  |  |  |
|  | **Conditions: Musculoskeletal system** |  |  |  | Swollen and stiff joints, arthritis, painful feet, back pain, serious bodily disability, and deformity |  |  |  |  |  |
|  | **Conditions: Nervous system** |  |  |  | Headaches, hot and cold spells, spells of severe dizziness, fainting, paralysis, numbness or tingling in any art of the body, epilepsy, nail biting, stuttering or stammer, sleep walking, bed wetting |  |  |  |  |  |
|  | **Conditions: Respiratory system** |  |  |  | Regularly coughing and sneezing, nose bleeds, severe colds, hay fever, asthma, severe soaking sweats at night, chronic chest conditions, Tuberculosis |  |  |  |  |  |
|  | **Conditions: Skin** |  |  |  | Sensitive and tender skin, cuts that do not heal in a timely manner, flushed face, severe itching, sweating in cold weather, rashes, boils |  |  |  |  |  |
|  | **Frequency of illness** |  |  |  | Frequency of illness, being confined to a bed by illness, being in poor health, being made miserable by poor health |  |  |  |  |  |
|  | **Role-physical problems** |  |  |  |  |  | Limitations in kind of work or activities due to physical health and whether respondent accomplished less due to physical health |  |  |  |
|  | **Somatic health symptoms** |  |  |  |  |  |  |  |  | NR |
| **Relationship with family and friends** | **Relationship with family and friends** |  |  | NR |  |  |  |  |  |  |
| **Stigma and discrimination** | **Stigma and discrimination** |  |  | NR |  |  |  |  |  |  |
| **Wellbeing** | **Wellbeing** | Positive feelings towards life, including hope, enjoyment, and happiness |  |  |  |  |  | Overall wellbeing |  |  |
|  | **Emotional wellbeing** |  |  | NR |  |  |  |  |  |  |

# Meta-summary of physical health

| **Meta-summary of physical health** | | | | | | | |
| --- | --- | --- | --- | --- | --- | --- | --- |
| **Themes** | **Dimensions** | **Questionnaires** | | | | | |
|  |  | **Australian Type 2 Diabetes Risk Assessment Tool [128]** | **Behavioral Risk Factor Surveillance System [34]** | **Physical Health Rating [129]** | **Self-developed scale by Greenberg et al. [130]** | **Somatic Symptom Scale [131]** | **WHO World Health Survey [132-133]** |
| **General health-related characteristics** |  |  |  |  |  |  |  |
|  | **Demographic measures** | Includes gender, age, country of birth, education, and employment |  |  |  |  |  |
|  | **Lifestyle measures** | Includes smoking, eating vegetables or fruits daily, and physical activity per week |  |  |  |  |  |
|  | **Simple anthropometric measures** | Includes body mass index, blood pressure and waist circumference |  |  |  |  |  |
|  | **Severity of sleep problems** |  |  |  |  |  | NR |
| **Physical health** | **Physical health** |  | Poor physical health days in past month | NR | Self-rated physical health |  |  |
|  | **Immunological** |  |  |  |  | the most common symptoms in disorders that affect the immunological system |  |
|  | **Respiratory** |  |  |  |  | the most common symptoms in disorders that affect the respiratory system |  |
|  | **Cardiovascular** |  |  |  |  | the most common symptoms in disorders that affect the cardiovascular system |  |
|  | **Neurosensory** |  |  |  |  | the most common symptoms in disorders that affect the neurosensory system |  |
|  | **Gastrointestinal** |  |  |  |  | the most common symptoms in disorders that affect the gastrointestinal system |  |
|  | **Dermatological** |  |  |  |  | the most common symptoms in disorders that affect the dermatological system |  |
|  | **Genital-urinary** |  |  |  |  | the most common symptoms in disorders that affect the genital-urinary system |  |
|  | **Muscular** |  |  |  |  | the most common symptoms in disorders that affect the muscular system |  |
|  | **Female reproductive system** |  |  |  |  | the most common symptoms in disorders that affect the female reproductive system |  |

# Meta-summary of quality of life

| **Meta-summary of quality of life** | | | | | | | |
| --- | --- | --- | --- | --- | --- | --- | --- |
| **Themes** | **Dimensions** | **Questionnaires** | | | | | |
|  |  | **CarerQoL [134]** | **EuroQoL [135]** | **Health Utilities Index [136]** | **Quality of Life Index [137]** | **Quality of Life Measure [138]** | **World Health Organization Quality of Life [139-143]** |
| **Environment** | **Environment** |  |  |  |  |  | Includes financial resources, freedom, physical safety and security, health, and social care: accessibility and quality, home environment, opportunities for acquiring new information and skills, participation in and opportunities for recreation/ leisure activities, physical environment (pollution/ noise/ traffic/ climate), and transport |
|  | **Finances** | Caregiver has financial problems because of my care tasks |  |  |  | NR |  |
|  | **Residence** |  |  |  |  | NR |  |
|  | **Socioeconomic** |  |  |  | Includes standard of living, financial independence, home, job/unemployment, neighborhood, overall conditions in U.S.A., friends, emotional support from others, and education |  |  |
| **Family** | **Family** |  |  |  | Includes family's happiness, children, relationship with spouse/significant other, and family's health |  |  |
| **Health and functioning** | **Health and functioning** |  |  |  | Includes usefulness to others, physical independence, ability to meet family responsibilities, own health, stress or worries, leisure time activities, potential for a happy old age/retirement, ability to travel on vacations, potential for long life, sex life, health care, and dialysis treatment |  |  |
|  | **Fertility** |  |  | Includes ability to have children with a fertile spouse, difficulty in having children with a fertile spouse and unable to have children with a fertile spouse |  |  |  |
|  | **Self-care** |  | Degree to which respondent experiences difficulties washing or dressing themselves on the day of measurement | Includes eating, bathing, dressing, and using the toilet normally for age, eating, bathing, dressing, and using the toilet independently with difficulty, requires mechanical equipment to eat, bathe, dress or use the toilet independently and requires the help of another person to eat, bathe, dress, or use the toilet |  |  |  |
|  | **Usual activities** |  | Degree to which respondent experiences difficulties performing usual activities on the day of measurement (e.g., work, study, housework,  or leisure activities) |  |  |  |  |
| **Mental health** | **Mental health** | Caregiver has problems with their own mental health (e.g., stress, fear, gloominess, depression, concern about the future) |  |  |  | NR |  |
|  | **Anxiety/ depression** |  | Degree to which respondent experiences depression or anxiety on the day of measurement |  |  |  |  |
|  | **Cognition** |  |  | Includes learning and remembering schoolwork normally for age, learning and remembering schoolwork more slowly than classmates as judged by parents and/or teachers and learning and remembering very slowly and usually requires special education assistance |  |  |  |
|  | **Emotion** |  |  | Includes being generally happy and free from worry, occasionally fretful, angry, irritable, anxious, depressed or suffering night terrors, almost always fretful, angry, irritable, anxious or depressed, extremely fretful, angry, irritable, anxious, or depressed usually requiring hospitalization of psychiatric institutional care |  |  |  |
|  | **Psychological/spiritual** |  |  |  | Includes satisfaction with life, happiness in general, satisfaction with self in general, achievement of personal goals, peace of mind, personal appearance, and personal faith in God |  | Includes bodily image and appearance, negative feelings, positive feelings, self-esteem, spirituality/ religion/ personal beliefs, and thinking, learning, memory, and concentration |
| **Overall satisfaction with life** | **Overall satisfaction with life** |  |  |  |  | NR |  |
|  | **Positivity about the future** |  |  |  |  | NR |  |
|  | **Sense of fulfilment** | Caregiver has fulfilment from carrying out care tasks |  |  |  | NR |  |
| **Physical health** | **Physical health** | Caregiver has problems with their own physical health (e.g., more often sick, tiredness, physical stress) |  |  |  | NR | Includes activities of daily living, dependence on medicinal substances and medical aids, energy and fatigue, mobility, pain and discomfort, sleep and rest, and work capacity |
|  | **Mobility** |  | Degree to which respondent experiences difficulties walking about on the day of measurement | Includes being able to walk, bend, lift, jump and run normally for age, walk, bend, lift, jump or run with some limitations but does not require help, requires mechanical equipment (i.e., canes, crutches, braces, or wheelchair) to walk or get around independently and requires the help of another person to walk and get around and requires mechanical equipment as well |  |  |  |
|  | **Pain** |  | Degree to which respondent experiences pain on the day of measurement | Includes free of pain and discomfort, occasional pain (i.e., discomfort relieved by non-prescription drugs or self-control activity without disruption of normal activities), frequent pain (i.e., discomfort relieved by oral medicines with occasional disruption of normal activities), frequent pain- frequent disruption of normal activities (i.e., discomfort requires prescription narcotics for relief) and severe pain (i.e., pain not relieved by drugs and constantly disrupts normal activities) |  |  |  |
|  | **Sensation** |  |  | Includes ability to see, hear and speak normally for age, requires equipment to see or hear or speak, sees, hears, or speaks with limitations even with equipment and blind, deaf, or mute |  |  |  |
| **Relational dimension** | **Relational dimension** | Caregiver has relational problems with the care recipient e.g., he/she is very demanding or behaves differently; we have communication problems) |  |  |  |  |  |
| **Social relationships** | **Social relationships** | Caregiver has problems combining their care tasks with their own daily activities (e.g., household activities, work, study, family, leisure activities) |  |  |  | Relationships with others | Includes personal relationships, social support, sexual activity |
| **Support with lending care** | **Support with lending care** | Caregiver has support with carrying out their care tasks when they need it (e.g., from family, friends, neighbors, acquaintances) |  |  |  | Access to support |  |

# Meta-summary of satisfaction

| **Meta-summary of satisfaction** | | | | | | | | |
| --- | --- | --- | --- | --- | --- | --- | --- | --- |
| **Themes** | **Dimensions** | **Questionnaires** | | | | | | |
|  |  | **Caregiver Well-Being and Support [120]** | **Family Satisfaction Scale [144]** | **Family Satisfaction Scale [145]** | **Marital Adjustment Test [146]** | **Marital Satisfaction Questionnaire for Older Persons [147]** | **Satisfaction with Life Scale [148]** | **Self-developed survey by the Private Mental Health Consumer Caregiver Network [117]** |
| **Satisfaction with life** | **Satisfaction with life** |  |  |  |  |  | Includes their life being close to their ideal, conditions of their life are excellent, satisfaction with their life, having the important things they want in life and changing nothing if they could live their life over. |  |
| **Satisfaction with caregiver support** |  |  |  |  |  |  |  |  |
|  | **Information and advice for caregivers** | NR |  |  |  |  |  |  |
|  | **Your involvement in treatment and care planning** | NR |  |  |  |  |  |  |
|  | **Support from medical and/or care staff** | NR |  |  |  |  |  |  |
|  | **Support from other caregivers** | NR |  |  |  |  |  |  |
|  | **Taking a break ("respite")** | NR |  |  |  |  |  |  |
|  | **Perceived support general practitioner** |  |  |  |  |  |  | NR |
|  | **Perceived support psychiatrist** |  |  |  |  |  |  | NR |
|  | **Perceived support psychologist** |  |  |  |  |  |  | NR |
|  | **Perceived support mental health worker** |  |  |  |  |  |  | NR |
|  | **Perceived support occupational therapist** |  |  |  |  |  |  | NR |
|  | **Perceived support social worker** |  |  |  |  |  |  | NR |
|  | **Perceived support other** |  |  |  |  |  |  | NR |
| **Family satisfaction** | **Family satisfaction** |  |  | The degree to which one is generally satisfied with one’s family of origin and the constituent relationships imbedded therein. |  |  |  |  |
|  | **Adaptability** |  | The quality and expression of leadership and organization, role relationship, and relationship rules and negotiations. |  |  |  |  |  |
|  | **Cohesion** |  | The emotional bonding that family members have toward one another. |  |  |  |  |  |
|  | **Communication** |  | The amount of change in family leadership, role relationships, and relationship rules. |  |  |  |  |  |
| **Marital satisfaction** |  |  |  |  |  |  |  |  |
|  | **Sexual congeniality** |  |  |  | NR | e.g., "frequency of sexual relations," "quality of sexual relations" |  |  |
|  | **Compatibility** |  |  |  | NR |  |  |  |
|  | **Communication/ companionship** |  |  |  |  | e.g., "consideration shown by spouse," "how decisions are made" |  |  |
|  | **Satisfaction with spouse's health** |  |  |  |  | e.g., "spouse's physical health" |  |  |

# Meta-summary of social impact

| **Meta-summary of social impact** | | | | | | | |
| --- | --- | --- | --- | --- | --- | --- | --- |
| **Themes** | **Dimensions** | **Questionnaires** | | | | | |
|  |  | **Affiliate Stigma Scale [149]** | **Friendship Scale [150]** | **Kreisman's Family Rejection Scale [151]** | **Self-developed scale by Griffiths et al. [152]** | **Social Network Questionnaire [18]** | **Wisconsin Longitudinal Study (WLS) Survey [127]** |
| **Perceived stigma** | **Perceived stigma** |  |  |  | Caregivers'  beliefs about the attitudes of others |  |  |
|  | **Behavioral** | Avoid communicating with their family member with mental illness, dare not tell others that they have a family member with a mental illness, reduce going out with family member with mental illness, cut down contacts with friends and relatives, and keeping a low profile when with family member with mental illness |  |  |  |  |  |
|  | **Cognitive** | Includes feeling discriminated against, feeling incompetent, losing face, having a damaged reputation, feeling lesser to others, and people's attitudes towards them turning sour due to their family member with a mental illness |  |  |  |  |  |
| **Personal stigma** | **Personal stigma** |  |  |  | Participants' personal attitudes |  |  |
|  | **Affective** | Includes feeling inferior, emotional, sad, helpless, and under great pressure because a family member has a mental disorder or intellectual disability. Additionally, the behavior of the family member with a mental illness or intellectual disability makes them feel embarrassed and worrying if other people know if they have a family member with mental illness/intellectual disability |  |  |  |  |  |
| **Social contact** |  |  |  |  |  |  |  |
|  | **Easy to get in touch with others when they need it** |  | NR |  |  |  |  |
|  | **Easy to relate to others** |  | NR |  |  |  |  |
|  | **Frequency of social contact** |  |  |  |  | NR |  |
|  | **Having someone to share feelings with** |  | NR |  |  |  |  |
| **Social isolation** |  |  |  |  |  |  |  |
|  | **Feeling alone and friendless** |  | NR |  |  |  |  |
|  | **Feeling isolated from other people** |  | NR |  |  |  |  |
|  | **Feeling separated when with other people** |  | NR |  |  |  |  |
| **Social rejection** | **Social rejection** |  |  | NR |  |  |  |
| **Social participation** | **Social participation** |  |  |  |  |  | Social participations in activity organizations, including charitable organizations and civic groups |
| **Social support** |  |  |  |  |  |  |  |
|  | **Emotional social support** |  |  |  |  | NR |  |
|  | **Practical social support** |  |  |  |  | NR |  |
| **Quality of an intimate supportive relationship** | **Quality of an intimate supportive relationship** |  |  |  |  | NR |  |

# Meta-summary of work impact

| **Meta-summary of work impact** | | | | |
| --- | --- | --- | --- | --- |
| **Themes** | **Dimensions** | **Questionnaires** | | |
|  |  | **Insurance-Medicine-All-Sweden (IMAS) Study [46]** | **Work Productivity and Impairment Questionnaire [153]** | **Self-developed scale by Csoboth et al. [126]** |
| **Activity impairment** | **Activity impairment** |  | The percentage of impairment in daily activities due to health problems. |  |
| **Labor force participation** | **Labor force participation** | Unemployment (>180 days) |  | Includes being employed full time, part time or being self-employed. |
| **Low or no income** | **Low or no income** | NR |  |  |
|  | **Social welfare benefit** | NR |  |  |
|  | **Disability pension** | NR |  |  |
| **Overall work productivity loss** | **Overall work productivity loss** |  | The percentage of overall work impairment due to health problems. |  |
|  | **Absenteeism** | Sickness absence (>90 days) | The percentage of work time missed due to health problems. |  |
|  | **Presenteeism** |  | The percentage of impairment while at work, due to health problems. |  |

# Meta-summary of other caregiving consequences

| **Meta-summary of other caregiving consequences** | | | | | | | | | | | |
| --- | --- | --- | --- | --- | --- | --- | --- | --- | --- | --- | --- |
| **Themes** | **Dimensions** | **Questionnaires** | | | | | | | | | |
|  |  | **Additional Involvement Evaluation Questionnaire Modules [154]** | **COPE Index [155-156]** | **Family Life Difficulty Scale [151]** | **Involvement Evaluation Questionnaire [154, 157-158]** | **Self-developed visual analogue scale by Heru & Ryan [25]** | **Self-developed questionnaire by Corsentino et al. [159]** | **Social Behavior Assessment Scale [160]** | **Stress Related Growth Scale- Revised [161]** | **Self-developed questionnaire by Lohrer et al. [162]** | **Wisconsin Longitudinal Study (WLS) Survey [127]** |
| **Caregiver consequences: negative** | **Caregiver consequences: negative** |  | Negative impact of caring; e.g., ‘‘Do you find caregiving too demanding?’’; ‘‘Does caregiving cause difficulties in your relationship with your friends?’’; ‘‘Does caregiving have a negative effect on your physical health?’’; ‘‘Does caregiving cause difficulties in your relationship with your family?’’; ‘‘Does caregiving cause you financial difficulties?’’; ‘‘Do you feel trapped in your role as caregiver?’’; and ‘‘Does caregiving have a negative effect on your emotional well-being?’’ |  |  |  |  | Impact of the illness on the caregiver's work, social, and leisure time |  |  |  |
|  | **Difficulties and adversities in managing finances** |  |  | NR |  |  |  |  |  |  | Financial distress |
|  | **Difficulties and adversities concerning control over their personal lives while caring for a member with mental illness** |  |  | NR |  |  |  |  |  |  |  |
|  | **Difficulties and adversities in managing social and family life** |  |  | NR |  |  |  |  |  |  |  |
|  | **Stress** |  |  |  |  |  | NR |  |  |  |  |
|  | **Worrying** |  |  |  | Painful interpersonal cognitions, such as concern about the patient's safety and future, general health, and health care |  |  |  |  |  |  |
|  | **Caregiver's concerns** |  |  |  |  |  | NR |  |  |  |  |
| **Caregiver consequences: positive** | **Caregiver consequences: positive** |  | e.g., ‘‘Do you feel you cope well as a caregiver?’’; ‘‘Do you find caregiving worthwhile?’’; ‘‘Do you have a good relationship with the person you care for?’’; and ‘‘Do you feel that anyone appreciates you as a caregiver?’’ |  |  |  |  |  |  |  |  |
|  | **Affect-regulation** |  |  |  |  |  |  |  | e.g., "not getting angry about things" |  |  |
|  | **Religiousness** |  |  |  |  |  |  |  | e.g., "trust in God" |  |  |
|  | **Treatment of others** |  |  |  |  |  |  |  | e.g., "respect for others' feelings and beliefs" |  |  |
|  | **Self-understanding** |  |  |  |  |  |  |  | e.g., "accepting myself as less than perfect" |  |  |
|  | **Belongingness** |  |  |  |  |  |  |  | e.g., "feeling as if I am part of a community" |  |  |
|  | **Personal strength** |  |  |  |  |  |  |  | e.g., "confidence in self" |  |  |
|  | **Optimism** |  |  |  |  |  |  |  | e.g., "looking at things in a positive way" |  |  |
|  | **Life satisfaction** |  |  |  |  |  |  |  | NR |  |  |
|  | **Reward** |  |  |  |  | Amount of reward caregiver's felt caring for their relative |  |  |  |  |  |
| **Caregiver's use of professional help** | **Caregiver's use of professional help** | NR |  |  |  |  |  |  |  |  |  |
|  | **Quality of support in caring** |  | e.g., ‘‘Do you feel well supported by friends or neighbors?’’; ‘‘Do you feel supported by your family?’’; ‘‘Do you feel well supported by health and social services?’’; and ‘‘Overall, do you feel well supported in your role of caregiver?’’ |  |  |  |  |  |  |  |  |
| **Consequences for caregiving situation** |  |  |  |  |  |  |  |  |  |  |  |
|  | **Planning future care** |  |  |  |  |  | NR |  |  |  |  |
|  | **Supervision** |  |  |  | Caregiver's task of guarding the patient's medicine intake, sleep, and dangerous behavior |  |  |  |  |  |  |
|  | **Urging** |  |  |  | Activation and motivation, e.g., stimulating the patient to take care of themselves, to eat enough and to undertake activities |  |  |  |  |  |  |
|  | **Time spent in care related activities** |  |  |  |  |  |  |  |  | Extent to which respondents reported aiding their sibling with illness in specific care related activities over the past month, including personal hygiene, housework, & meal preparation, money management assistance, shopping, transportation, and case management activities |  |
|  | **Involvement in crisis management** |  |  |  |  |  |  |  |  | Frequency of crisis episodes in which the sibling respondent was involved in providing assistance to their relative during the past year |  |
|  | **Changes in caregiving due to ageing care recipient** |  |  |  |  |  | NR |  |  |  |  |
| **Consequences for care recipient** |  |  |  |  |  |  |  |  |  |  |  |
|  | **Patient problem behaviors** |  |  |  |  |  |  | e.g., violence, unpredictability |  |  |  |
|  | **Patient role dysfunction at work and at home** |  |  |  |  |  |  | NR |  |  |  |
| **Tension** | **Tension** |  |  |  | Strained interpersonal atmosphere between patient and relatives |  |  |  |  |  |  |
| **Financial expenses** | **Financial expenses** | Extra financial expenses incurred on behalf of the patient |  |  |  |  |  |  |  | Amount of money caregiver spent over the past month related to the care and assistance of their relative with mental illness, including money provided as well as direct expenses related to the care of their sibling |  |

# References

1. Struening E, Vine P, Stueve A, Kreisman D, Link B, Ellis M, et al. The Family Impact Study. New York (NY): The New York State Psychiatric Institute; 1993.
2. Reinhard SC, Gubman GD, Horwitz AV, Minsky S. Burden assessment scale for families of the seriously mentally ill. Eval Program Plann. 1994;17(3): 261-269. doi: 10.1016/0149-7189(94)90004-3.
3. Sell H, Thara R, Padmavati R, Kumar S. The Burden Assessment Schedule (BAS). WHO Regional Office for South-East Asia; 1998. Regional Publication No. 27. Available from: https://apps.who.int/iris/handle/10665/205977.
4. Raenker S, Hibbs R, Goddard E, Naumann U, Arcelus J, Ayton A, et al. Caregiving and coping in carers of people with anorexia nervosa admitted for intensive hospital care. Int J Eat Disord. 2013;46(4): 346-354. doi: 10.1002/eat.22068.
5. Novak M, Guest C. Application of a multidimensional caregiver burden inventory. Gerontologist. 1989;29(6): 798-803. doi: 10.1093/geront/29.6.798.
6. Elmståhl S, Malmberg B, Annerstedt L. Caregiver's burden of patients 3 years after stroke assessed by a novel caregiver burden scale. Arch Phys Med Rehabil. 1996;77(2): 177-182. doi: 10.1016/S0003-9993(96)90164-1.
7. Robinson BC. Validation of a Caregiver Strain Index. J Gerontol. 1983;38(3): 344-348. doi: 10.1093/geronj/38.3.344.
8. Brannan AM, Heflinger CA, Bickman L. The Caregiver Strain Questionnaire: Measuring the Impact on the Family of Living with a Child with Serious Emotional Disturbance. J Emot Behav Disord. 1997;5(4): 212-222. doi: 10.1177/106342669700500404.
9. Bickman L, Athay MM, Riemer M, Lambert EW, Kelley SD, Breda C, et al. Manual of the Peabody treatment progress battery. Nashville (TN): Vanderbilt University; 2007.
10. Sepulveda AR, Whitney J, Hankins M, Treasure J. Development and validation of an Eating Disorders Symptom Impact Scale (EDSIS) for carers of people with eating disorders. Health Qual Life Outcomes. 2008;6: 28. doi: 10.1186/1477-7525-6-28.
11. Martínez A, Nadal S, Beperet M, Mendióroz P, grupo Psicost. Sobrecarga de los cuidadores familiares de pacientes con esquizofrenia: factores determinantes [Burden upon family caregivers of patients with schizophrenia: determining factors]. Anales Sis San Navarra. 2000;23: 101-110.
12. Vilaplana M, Ochoa S, Martínez A, Villalta V, Martinez-Leal R, Puigdollers E, et al. Validación en población española de la entrevista de carga familiar objetiva y subjetiva (ECFOS-II). Validación en población española del ECFOS-II [Validation in Spanish population of the family objective and subjective burden interview (ECFOS-II). Validity of ECFOS-II in Spanish population]. Actas Esp Psiquiatr. 2007;35(0):00-00.
13. Östman M, Hansson L. Family burden and care participation: A test-retest reliability study of an interview instrument concerning families with a severely mentally ill family member. Nord J Psychiatry. 2000;54(5): 327-332. doi: 10.1080/080394800457156.
14. Tessler RC, Fisher GA, Gamache GM. The family burden interview schedule: Manual. Amherst (MA): Social and Demographic Research Institute; 1992.
15. Ostman M, Wallsten T, Kjellin L. Family burden and relatives' participation in psychiatric care: are the patient's diagnosis and the relation to the patient of importance?. Int J Soc Psychiatry. 2005;51(4): 291-301. doi: 10.1177/0020764005057395.
16. Pai S, Kapur RL. The burden on the family of a psychiatric patient: development of an interview schedule. Br J Psychiatry. 1981;138: 332-335. doi: 10.1192/bjp.138.4.332.
17. Madianos M, Economou M, Dafni O, Koukia E, Palli A, Rogakou E. Family disruption, economic hardship and psychological distress in schizophrenia: can they be measured?. Eur Psychiatry. 2004;19(7): 408-414. doi: 10.1016/j.eurpsy.2004.06.028.
18. Morosini PL, Roncone R, Veltro F, Palomba U, Casacchia M. Routine assessment tool in psychiatry: the questionnaire of family attitudes and burden. Ital J Psychiatry Behav Sci. 1991;1(1): 95-101.
19. Kluiter H, Kramer JJ, Wiersma D. Interview for Measuring the Burden on the Family (IBF). Groningen (NL): University of Groningen; 1998.
20. Ostman M. The burden experienced by relatives of those with a severe mental illness - differences between those living with and those living apart from the patient. J Intensive Care. 2007;3(1): 35-43. doi: 10.1017/S1742646407001082.
21. Stueve A, Vine P, Struening EL. Perceived burden among caregivers of adults with serious mental illness: comparison of black, Hispanic, and white families. Am J Orthopsychiatry. 1997;67(2): 199-209. doi: 10.1037/h0080223
22. Levene JE, Lancee WJ, Seeman MV. The perceived family burden scale: measurement and validation. Schizophr Res. 1996;22(2): 151-157. doi: 10.1016/s0920-9964(96)00071-0.
23. Gater A, Rofail D, Marshall C, Tolley C, Abetz-Webb L, Zarit SH, et al. Assessing the Impact of Caring for a Person with Schizophrenia: Development of the Schizophrenia Caregiver Questionnaire. Patient. 2015;8(6): 507-520. doi: 10.1007/s40271-015-0114-3.
24. Goodman M, Patil U, Triebwasser J, Hoffman P, Weinstein ZA, New A. Parental burden associated with borderline personality disorder in female offspring. J Pers Disord. 2011;25(1): 59-74. doi: 10.1521/pedi.2011.25.1.59.
25. Heru AM, Ryan CE. Burden, reward and family functioning of caregivers for relatives with mood disorders: 1-year follow-up. J Affect Disord. 2004;83(2-3): 221-225. doi: 10.1016/j.jad.2004.04.013.
26. Hielscher E, Diminic S, Kealton J, Harris M, Lee YY, Whiteford H. Hours of Care and Caring Tasks Performed by Australian Carers of Adults with Mental Illness: Results from an Online Survey. Community Ment Health J. 2019;55(2): 279-295. doi: 10.1007/s10597-018-0244-x.
27. Pot AM, van Dyck R, Deeg DJ. Ervaren druk door informele zorg: constructie van een schaal [Perceived stress caused by informal caregiving: construction of a scale]. Tijdschr Gerontol Geriatr. 1995;26: 214-219.
28. Gilleard CJ. Living with Dementia: Community Care of the Elderly Mental Infirm. London (UK): Croom Helm; 1984.
29. Martín Carrasco M, Salvadó I, Nadal Álava S, Miji LC; Rico JM, Lanz P, et al. Adaptación para nuestro medio de la escala de sobrecarga del cuidador (Caregiver Burden Interview) de Zarit [Adaptation for our setting of the Zarit Caregiver Burden Interview scale]. Rev Gerontol. 1996; 6(4): 338-345.
30. Zarit SH, Reever KE, Bach-Peterson J. Relatives of the impaired elderly: correlates of feelings of burden. Gerontologist. 1980;20(6): 649-655. doi: 10.1093/geront/20.6.649.
31. Cirici Amell R, Cobo J, Castanyer MM, Giménez Gómez N. Gender and other factors influencing the burden of care in relatives of people diagnosed with schizophrenia and schizophrenia spectrum disorders. Int J Cult Ment Health. 2018;11(4): 638-652. doi: 10.1080/17542863.2018.1479764.
32. Ozlu A, Yildiz M, Aker T. Burden and burden-related features in caregivers of schizophrenia patients. Düşünen Adam. 2015;28(2): 147-153. doi: 10.5350/DAJPN2015280207.
33. Arai Y, Kudo K, Hosokawa T, Washio M, Miura H, Hisamichi S. Reliability and validity of the Japanese version of the Zarit Caregiver Burden interview. Psychiatry Clin Neurosci. 1997;51(5): 281-287. doi: 10.1111/j.1440-1819.1997.tb03199.x.
34. Centers for Disease Control and Prevention. Behavioral Risk Factor Surveillance System Survey Data. Atlanta (GA): Department of Health and Human Services; 2016.
35. Autism Speaks. Autism Speaks Global Public Health Initiative [Internet]. New York (NY): Autism Speaks; 2016. Available from: [https://www.autismspeaks.org/science-blog?article_type[2196]=2196&article_type[2196]=2196](https://www.autismspeaks.org/science-blog?article_type%5b2196%5d=2196&article_type%5b2196%5d=2196).
36. Wancata J, Krautgartner M, Berner J, Scumaci S, Freidl M, Alexandrowicz R, et al. The “Carers’ needs assessment for Schizophrenia” An instrument to assess the needs of relatives caring for schizophrenia patients. Soc Psychiatry Psychiatr Epidemiol. 2006;41: 221-229. doi: 10.1007/s00127-005-0021-3.
37. Statistics Canada. General Social Survey Cycle 26: Caregiving and Care Receiving 2012 Study Documentation. Ottawa (CA): Statistics Canada; 2013.
38. Barrowclough C, Marshall M, Lockwood A, Quinn J, Sellwood W. Assessing relatives' needs for psychosocial interventions in schizophrenia: a relatives' version of the Cardinal Needs Schedule (RCNS). Psychol Med. 1998;28(3): 531-542. doi: 10.1017/s003329179800662x.
39. Mulligan J, Sellwood W, Reid GS, Riddell S, Andy N. Informal caregivers in early psychosis: evaluation of need for psychosocial intervention and unresolved grief. Early Interv Psychiatry. 2013;7(3): 291-299. doi: 10.1111/j.1751-7893.2012.00369.x.
40. Sono T, Oshima I, Ito J. Family needs and related factors in caring for a family member with mental illness: adopting assertive community treatment in Japan where family caregivers play a large role in community care. Psychiatry Clin Neurosci. 2008;62(5): 584-590. doi: 10.1111/j.1440-1819.2008.01852.x.
41. Chamba R, Ahmad W, Hirst M, Lawton D, Beresford B. On the Edge: Minority Ethnic Families Caring for a Severely Disabled Child. Bristol (UK): Policy Press; 1999.
42. Marwitz J. The Family Needs Questionnaire. [Internet] The Center for Outcome Measurement in Brain Injury; 2000. Available from: <http://www.tbims.org/combi/fnq>.
43. Gupta S, Isherwood G, Jones K, van Impe K. Productivity loss and resource utilization, and associated indirect and direct costs in individuals providing care for adults with schizophrenia in the EU5. Clinicoecon Outcomes Res. 2015;7: 593-602. doi: 10.2147/CEOR.S94334.
44. Beecham J, Knapp M. Costing psychiatric interventions. In: Thornicroft G, editor. Measuring Mental Health Needs. 2nd ed. London (UK): Royal College of Psychiatrists; 2001. p. 200-224.
45. Dunst CJ, Jenkins V, Trivette CM. Enabling and Empowering Families: Principles and Guidelines for Practice. Cambridge (MA): Brookline Books; 1988. Family Support Scale; p. 153-174.
46. Mittendorfer-Rutz E, Rahman S, Tanskanen A, Majak M, Mehtälä J, Hoti F, et al. Burden for Parents of Patients With Schizophrenia-A Nationwide Comparative Study of Parents of Offspring With Rheumatoid Arthritis, Multiple Sclerosis, Epilepsy, and Healthy Controls. Schizophr Bull. 2019;45(4): 794-803. doi: 10.1093/schbul/sby130.
47. Cohen JW, Monheit AC, Beauregard KM, Cohen SB, Lefkowitz DC, Potter DE, et al. The Medical Expenditure Panel Survey: a national health information resource. Inquiry. 1996-1997;33(4): 373-389.
48. Perlick DA, Hohenstein JM, Clarkin JF, Kaczynski R, Rosenheck RA. Use of mental health and primary care services by caregivers of patients with bipolar disorder: a preliminary study. Bipolar Disord. 2005;7(2): 126-135. doi: 10.1111/j.1399-5618.2004.00172.x.
49. Sherr ME, Stamey JD, Garland D. A faith practices scale for the church. Fam Community Health. 2009;23(1): 27-36.
50. Jewell TC, Stein CH. Parental influence on sibling caregiving for people with severe mental illness. Community Ment Health J. 2002;38(1): 17-33. doi: 10.1023/a:1013903813940.
51. Leith JE, Jewell TC, Stein CH. Caregiving Attitudes, Personal Loss, and Stress-Related Growth Among Siblings of Adults with Mental Illness. J Child Fam Stud. 2018;27: 1193-1206.
52. Antonovsky A. Unraveling the mystery of health: how people manage stress and stay well. San Francisco (CA): Jossey-Bass Publishers; 1987.
53. Vandereycken W. Validity and reliability of the Anorectic Behavior Observation Scale for parents. Acta Psychiatr Scand. 1992;85(2): 163-166. doi: 10.1111/j.1600-0447.1992.tb01462.x.
54. Lobban F, Barrowclough C, Jones S. Assessing cognitive representations of mental health problems. II. The illness perception questionnaire for schizophrenia: Relatives' version. Br J Clin Psychol. 2005;44(Pt 2): 163-179. doi: 10.1348/014466504X19785.
55. Schiffman J, Kline E, Reeves G, Jones A, Medoff D, Lucksted A, et al. Differences Between Parents of Young Versus Adult Children Seeking to Participate in Family-to-Family Psychoeducation. Psychiatr Serv. 2014;65(2): 247-250. doi: 10.1176/appi.ps.201300045.
56. Evans-Lacko S, Little K, Meltzer H, Rose D, Rhydderch D, Henderson C, et al. Development and psychometric properties of the Mental Health Knowledge Schedule. Can J Psychiatry. 2010;55(7): 440-448. doi: 10.1177/070674371005500707.
57. Tanaka G. Development of the Mental Illness and Disorder Understanding Scale. Int J Japanese Sociol. 2003;12(1): 95-107. doi: 10.1111/j.1475-6781.2003.00045.x.
58. Link BG, Phelan JC, Bresnahan M, Stueve A, Pescosolido BA. Public conceptions of mental illness: labels, causes, dangerousness, and social distance. Am J Public Health. 1999;89(9): 1328-1333. doi: 10.2105/ajph.89.9.1328.
59. Stocker CM, Lanthier RP, Furman W. Sibling Relationships in Early Adulthood. J Fam Psychol. 1997;11(2): 210-221.
60. Wiedemann G, Rayki O, Feinstein E, Hahlweg K. The Family Questionnaire: development and validation of a new self-report scale for assessing expressed emotion. Psychiatry Res. 2002;109(3): 265-279. doi: 10.1016/s0165-1781(02)00023-9.
61. Kavanagh DJ, O’Halloran P, Manicavasagar V, Clark D, Piatkowska, O, Tennant C, et al. The Family Attitude Scale: Reliability and validity of a new scale for measuring the emotional climate of families. Psychiatry Res. 1997;70(3): 185-195. doi: 10.1016/S0165-1781(97)00033-4.
62. Gerlsma C, van der Lubbe PM, van Nieuwenhuizen C. Factor analysis of the level of expressed emotion scale, a questionnaire intended to measure 'perceived expressed emotion'. Br J Psychiatry. 1992;160: 385-389. doi: 10.1192/bjp.160.3.385.
63. Caqueo-Urízar A, Gutiérrez-Maldonado J, Ferrer-García M, Peñaloza-Salazar C, Richards-Araya D, Cuadra-Peralta A. Attitudes and burden in relatives of patients with schizophrenia in a middle income country. BMC Fam Pract. 2011;12: 101.
64. Epstein NB, Baldwin LM, Bishop DS. The McMaster Family Assessment Device. J Marital Fam Ther. 1983;9(2): 171-180. doi: 10.1111/j.1752-0606.1983.tb01497.x.
65. Olson DH, Barnes H. Family Communication Scale. St Paul (MN): University of Minnesota; 1996.
66. Koren PE, DeChillo N, Friesen BJ. Measuring empowerment in families whose children have emotional disabilities: A brief questionnaire. Rehabil Psychol. 1992;37(4): 305-321. doi: 10.1037/h0079106.
67. Tessler R, Gamache G. Toolkit for Evaluating Family Experiences with Severe Mental Illness. Cambridge (MA): Human Services Research Institute; 1995.
68. Hoffman L, Marquis J, Poston D, Summers JA, Turnbull A. Assessing Family Outcomes: Psychometric Evaluation of the Beach Center Family Quality of Life Scale. J Marriage Fam. 2006;68(4): 1069-1083. doi: 10.1111/j.1741-3737.2006.00314.x.
69. Heru AM, Ryan CE. Burden, reward and family functioning of caregivers for relatives with mood disorders: 1-year follow-up. J Affect Disord. 2004;83(2-3): 221-225. doi: 10.1016/j.jad.2004.04.013.
70. Heru AM, Ryan CE, Vlastos K. Quality of life and family functioning in caregivers of relatives with mood disorders. Psychiatr Rehabil J. 2004;28(1): 67-71. doi: 10.2975/28.2004.67.71.
71. Verdiano DL. Family roles: An integration of theory, research, and practice. ProQuest Dissertations Publishing; 1986. Available from: <https://www.proquest.com/openview/7d17d5060f0a94125891a4fd22169966/1?pq-origsite=gscholar&cbl=18750&diss=y>.
72. Garland DR, Edmonds JA. Family life of Baptists. Fam Community Ministries. 2007;21(1): 6-21.
73. Bonner MJ, Hardy KK, Guill AB, McLaughlin C, Schweitzer H, Carter K. Development and validation of the parent experience of child illness. J Pediatr Psychol. 2006;31(3): 310-321. doi: 10.1093/jpepsy/jsj034.
74. Beck AT, Steer RA. Beck Anxiety Inventory Manual. San Antonio (TX): The Psychological Corporation; 1990.
75. Beck AT, Steer RA, Ball R, Ranieri W. Comparison of Beck Depression Inventories -IA and -II in psychiatric outpatients. J Pers Assess. 1996;67(3): 588-597. doi: 10.1207/s15327752jpa6703_13.
76. Hautzinger M, Kuehner C, Keller F. BDI-II Beck-Depressions-Inventar [BDI-II Beck Depression Inventory]. Pearson Assessment & Information GmbH; 2006.
77. Derogatis LR. BSI-18: Administration, Scoring and Procedures Manual. New York (NY): NCS Pearson; 2001.
78. Radloff LS. The CES-D Scale: A Self-Report Depression Scale for Research in the General Population. Appl Psychol Meas. 1997;1(3): 385-401. doi: 10.1177/014662167700100306.
79. Haro JM, Arbabzadeh-Bouchez S, Brugha TS, de Girolamo G, Guyer ME, Jin R, et al. Concordance of the Composite International Diagnostic Interview Version 3.0 (CIDI 3.0) with standardized clinical assessments in the WHO World Mental Health surveys. Int J Methods Psychiatr Res. 2006;15(4): 167-180. doi: 10.1002/mpr.196.
80. Kessler RC, Ustun TB, editors. The WHO World Mental Health Surveys: global perspectives on the epidemiology of mental disorders. New York (NY): Cambridge University Press; 2008.
81. Lovibond SH, Lovibond PF. Manual for the Depression Anxiety Stress Scales. Sydney (AU): Psychology Foundation; 1995.
82. Gratz KL, Roemer L. Multidimensional Assessment of Emotion Regulation and Dysregulation: Development, Factor Structure, and Initial Validation of the Difficulties in Emotion Regulation Scale. J Psychopathol Behav Assess. 2004;26: 41-54.
83. Beecham J, Knapp M. Costing psychiatric interventions. In: Thornicroft G, editor. Measuring Mental Health Needs. 2nd ed. London (UK): Royal College of Psychiatrists; 2001. p. 200-224.
84. Schmitz N, Kruse J, Tress W. Psychometric properties of the General Health Questionnaire (GHQ-12) in a German primary care sample. Acta Psychiatr Scand. 1999;100(6): 462-468. doi: 10.1111/j.1600-0447.1999.tb10898.x.
85. Lobo A, Pérez-Echeverría MJ, Artal J. Validity of the scaled version of the General Health Questionnaire (GHQ-28) in a Spanish population. Psychol Med. 1986;16(1): 135-140. doi: 10.1017/s0033291700002579.
86. Garyfallos G, Karastergiou A, Adamopoulou, A, Moutzoukis, C, Alagiozidou, E, Mala, D, et al. Greek version of the General Health Questionnaire: Accuracy of translation and validity. Acta Psychiatr Scand. 1991;84(4): 371-378. doi: 10.1111/j.1600-0447.1991.tb03162.x.
87. Huppert FA, Walters DE, Day NE, Elliott BJ. The factor structure of the General Health Questionnaire (GHQ-30): A reliability study on 6317 community residents. Br J Psychiatry. 1989;155: 178–185. doi: 10.1192/bjp.155.2.178.
88. Fernandez JL, Mielgo N. Escala de appreciacion de estres [Scale of stress perception]. Madrid (ES): TEA Ediciones; 2001.
89. González de Rivera JL, De las Cuevas C, Rodríguez M, Rodríguez F. Cuestionario de 90 Síntomas [90 Symptom Questionnaire]. Madrid (ES): TEA; 2002.
90. Sheikh JI, Yesavage JA. Geriatric Depression Scale (GDS): Recent evidence and development of a shorter version. Clin Gerontol. 1986;5(1-2): 165-173. doi: 10.1300/J018v05n01_09.
91. Goldberg D, Bridges K, Duncan-Jones P, Grayson D. Detecting anxiety and depression in general medical settings. BMJ. 1988;297: 897. doi: 10.1136/bmj.297.6653.897.
92. Struening EL, Stueve A, Vine P, Kreisman DE, Link BG, Herman DB. Factors associated with grief and depressive symptoms in caregivers of people with serious mental illness. Res Commun Ment Health, 1995;8: 91-124.
93. Zigmond AS, Snaith RP. The hospital anxiety and depression scale. Acta Psychiatr Scand. 1983;67(6): 361-370. doi: 10.1111/j.1600-0447.1983.tb09716.x.
94. Weiss D, Marmar C. The impact of event scale- revised. In: Wilson JP, Keane TM, editors. Assessing psychological trauma and PTSD. New York (NY): The Guilford Press; 1997.
95. Kessler RC, Andrews G, Colpe LJ, Mroczek DK, Normand SLT, Walters EE, et al. Short screening scales to monitor population prevalences and trends in non-specific psychological distress. Psychol Med. 2002;32(6): 959-976. doi: 10.1017/s0033291702006074.
96. Maslach CJ, Jackson SE, Leiter MP. Maslach Burnout Inventory Manual. Palo Alto (CA): Consulting Psychologists Press; 1996.
97. Berwick DM, Murphy JM, Goldman PA, Ware Jr JE, Barsky AJ, Weinstein MC. Performance of a five-item mental health screening test. Med Care. 1991;29(2): 169-176. doi: 10.1097/00005650-199102000-00008.
98. Miller F, Dworkin J, Ward M, Barone D. A preliminary study of unresolved grief in families of seriously mentally ill patients. Hosp Community Psychiatry. 1990;41(12): 1321-1325. doi: 10.1176/ps.41.12.1321.
99. Mroczek DK, Kolarz CM. The effect of age on positive and negative affect: a developmental perspective on happiness. J Pers Soc Psychol. 1998;75(5): 1333-1349. doi: 10.1037//0022-3514.75.5.1333.
100. Meyer TJ, Miller ML, Metzger RL, Borkovec TD. Development and validation of the Penn State Worry Questionnaire. Behav Res Ther. 1990;28(6): 487-495. doi: 10.1016/0005-7967(90)90135-6.
101. Cohen S, Kamarck T, Mermelstein R. A Global Measure of Perceived Stress. J Health Soc Behav. 1983;24: 385-396. doi: 10.2307/2136404.
102. McNair DM, Lorr M, Droppleman LF. Profile of Mood States (POMS) Manual. San Diego (CA): Educational and Industrial Testing Service; 1981.
103. Crawford JR, Smith G, Maylor EA, Della Sala S, Logie RH. The Prospective and Retrospective Memory Questionnaire (PRMQ): Normative data and latent structure in a large non-clinical sample. Memory. 2003;11(3): 261-275. doi: 10.1080/09658210244000027.
104. Ryff CD. Happiness is everything, or is it? Explorations on the meaning of psychological well-being. J Pers Soc Psychol. 1989;57(6): 1069-1081. doi: 10.1037/0022-3514.57.6.1069.
105. Spielberger CD, Gorsuch RL, Lushene R, Vagg PR, Jacobs GA. Manual for the State-Trait Anxiety Inventory. Palo Alto (CA): Consulting Psychologists Press; 1983.
106. Arrindell WA, Ettema JH. Symptom Checklist SCL-90. Handleiding bij een multi-dimensionele psychopathologie-indicator [Symptom Checklist SCL-90. Hanbook for a multi-dimensional psychopathology indicator]. Lisse (NL): Swets Test Publishers; 2003.
107. Derogatis LR, Cleary PA. Factorial invariance across gender for the primary symptom dimensions of the SCL-90. Br J Soc Clin Psychol. 1977;16(4): 347-356.
108. Lindenbaum K, Stroka MA, Linder R. Informal caregiving for elderly people with mental illnesses and the mental health of the informal caregivers. J Ment Health Policy Econ. 2014;17(3): 99-105.
109. Tennant R, Hiller L, Fishwick R, Platt S, Joseph S, Weich S, et al. The Warwick-Edinburgh Mental Well-being Scale (WEMWBS): development and UK validation. Health Qual Life Outcomes. 2007;5: 63. doi: 10.1186/1477-7525-5-63.
110. World Health Organization, World Bank. World report on disability 2011. [Internet]. 2011. Available from: <https://apps.who.int/iris/handle/10665/44575>
111. Lelliott P, Beevor A, Hogman G, Hyslop J, Lathlean J, Ward M. Carers' and users' expectations of services - carer version (CUES-C): A new instrument to support the assessment of carers of people with a severe mental illness. J Ment Health. 2003;2: 143-152. doi: 10.1080/0963823031000103452.
112. Szmukler GI, Burgess P, Herrman H, Benson A, Colusa S, Bloch S. Caring for relatives with serious mental illness: the development of the Experience of Caregiving Inventory. Soc Psychiatry Psychiatr Epidemiol. 1996;31(3-4): 137-148. doi: 10.1007/BF00785760.
113. Schwarzer R, Jerusalem M. Generalized Self-Efficacy Scale. In: Weinman J, Wright S, Johnston M, editors. Measures in Health Psychology: A User’s Portfolio. Causal and Control Beliefs. Windsor (UK): NFER-NELSON; 1995. p. 35-37.
114. Tokyo Metropolitan Institute of Gerontology. Primary Research about Health and Welfare Policy among Elderly and Disabled Persons: Research of Needs in Mitaka-City. Tokyo (JP): Tokyo Metropolitan Institute of Gerontology; 1997.
115. Nijman H, Bowers L, Oud N, Jansen G. Psychiatric nurses' experiences with inpatient aggression. Aggress Behav. 2005;31(3): 217-227. doi: 10.1002/ab.20038.
116. Labrum T, Solomon P. Safety Fears Held by Caregivers about Relatives with Psychiatric Disorders. Health Soc Work. 2018;43(3): 165-174. doi: 10.1093/hsw/hly013.
117. Lawn S, McMahon J. Experiences of family carers of people diagnosed with borderline personality disorder. J Psychiatr Ment Health Nurs. 2015;22(4): 234-243. doi: 10.1111/jpm.12193.
118. Jewell TC. Adult siblings of people with serious mental illness: The relationship between self -and -sibling -care beliefs and psychological adjustment. Ann Arbor (MI): ProQuest Dissertations Publishing; 1999. Available from: <https://www.proquest.com/openview/c51d582b6137eff8b179d43940e364d3/1?pq-origsite=gscholar&cbl=18750&diss=y>.
119. Struening E, Vine P, Stueve A, Kreisman D, Link B, Ellis M, et al. The Family Impact Study. New York (NY): The New York State Psychiatric Institute; 1993.
120. Quirk A, Smith S, Hamilton S, Lamping D, Lelliott P, Stahl D, et al. Development of the carer well‐being and support (CWS) questionnaire. Ment Health Rev J. 2012;17(3): 128-138. doi: 10.1108/13619321211287184.
121. Brodman K. Tests of personality: questionnaires. B. Cornell Medical Index-Health Questionnaire. In Weider A, editor. Contributions Toward Medical Psychology, Theory and Psychodiagnostic Methods. New York (NY): Ronald Press; 1953. p. 568-576.
122. Stewart AL, Hays RD, Ware Jr JE. The MOS short-form general health survey. Reliability and validity in a patient population. Med Care. 1988;26(7): 724-735. doi: 10.1097/00005650-198807000-00007.
123. Ware Jr JE, Sherbourne CD. The MOS 36-item short-form health survey (SF-36). I. Conceptual framework and item selection. Med Care. 1992;30(6): 473-483.
124. Ware Jr JE, Kosinski M, Keller SD. A 12-Item Short-Form Health Survey: construction of scales and preliminary tests of reliability and validity. Med Care. 1996;34(3): 220-233. doi: 10.1097/00005650-199603000-00003.
125. Ali L, Krevers B, Skärsäter I. Caring Situation, Health, Self-efficacy, and Stress in Young Informal Carers of Family and Friends with Mental Illness in Sweden. Issues Ment Health Nurs. 2015;36(6): 407-415. doi: 10.3109/01612840.2014.1002644.
126. Csoboth C, Witt EA, Villa KF, O’Gorman C. The humanistic and economic burden of providing care for a patient with schizophrenia. Int J Soc Psychiatry. 2015;61(8): 754-761. doi: 10.1177/0020764015577844.
127. Hauser RM, Sewell WH, Logan JA, Hauser TS, Ryff C, Caspi A, et al. The Wisconsin Longitudinal Study: Adults As Parents And Children At Age 50. IASSIST Q. 1992;16(1-2): 23. doi: 10.29173/iq631.
128. Department of Health and Ageing. The Australian Type 2 Diabetes Risk Assessment Tool [Internet]. Canaberra (AU): Australian Government Department of Health; 2010. Available from: <https://www.health.gov.au/resources/apps-and-tools/the-australian-type-2-diabetes-risk-assessment-tool-ausdrisk>.
129. Pakenham KI, Dadds MR, Terry DJ. Relationships between adjustment to HIV and both social support and coping. J Consult Clin Psychol. 1994;62(6): 1194-1203. doi: 10.1037//0022-006x.62.6.1194.
130. Greenberg JS, Seltzer MM, Krauss MW, Chou RJA, Hong J. The effect of quality of the relationship between mothers and adult children with schizophrenia, autism, or down syndrome on maternal well-being: the mediating role of optimism. Am J Orthopsychiatry. 2004;74(1): 14-25. doi: 10.1037/0002-9432.74.1.14.
131. Sandin B, Chorot P. Escala de Síntomas Somáticos Revisada (ESS-R) [Somatic Symptom Scale- Revised (ESS-R)]. Madrid (ES): Universidad Nacional de Educación a Distancia; 1995.
132. World Health Organization, World Bank. World report on disability 2011. [Internet]. 2011. Available from: <https://apps.who.int/iris/handle/10665/44575>
133. Koyanagi A, DeVylder JE, Stubbs B, Carvalho AF, Veronese N, Haro JM, et al. Depression, sleep problems, and perceived stress among informal caregivers in 58 low-, middle-, and high-income countries: A cross-sectional analysis of community-based surveys. J Psychiatr Res. 2018;96: 115-123. doi: 10.1016/j.jpsychires.2017.10.001.
134. Brouwer WBF, van Exel NJA, van Gorp B, Redekop WK. The CarerQol instrument: a new instrument to measure care-related quality of life of informal caregivers for use in economic evaluations. Qual Life Res. 2006;15(6): 1005-1021. doi: 10.1007/s11136-005-5994-6.
135. Rabin R, de Charro F. EQ-5D: a measure of health status from the EuroQol Group. Ann Med. 2001;33(5): 337-343. doi: 10.3109/07853890109002087.
136. Horsman J, Furlong W, Feeny D, Torrance G. The Health Utilities Index (HUI®): concepts, measurement properties and applications. Health Qual Life Outcomes. 2003;1: 54. doi: 10.1186/1477-7525-1-54.
137. Ferrans CE, Powers MJ. Psychometric assessment of the Quality of Life Index. Res Nurs Health. 1992;15(1): 29-38. doi: 10.1002/nur.4770150106.
138. Herrema R, Garland D, Osborne M, Freeston M, Honey E, Rodgers J. Mental Wellbeing of Family Members of Autistic Adults. J Autism Dev Disord. 2017;47(11): 3589-3599. doi: 10.1007/s10803-017-3269-z.
139. Angermeyer MC, Kilian R, Matschinger H. WHOQOL-100 und WHOQOL-BREF [WHOQOL-100 and WHOQOL-BREF]. Göttingen (DE): Hogrefe; 2000.
140. Eser E, Fidaner H, Fidaner C, Eser SY, Elbi H, Göker E. WHOQOL-100 ve WHOQOL-BREF'in psikometrik özellikleri [Psychometric properties of the WHOQOL-100 and WHOQOL-BREF]. Psikiyatri Psikoloji Psikofarmakoloji (3P) Dergisi, 1999;7(Suppl 2): 23-40.
141. Hawthorne G, Herrman H, Murphy B. Interpreting the WHOQOL-Brèf: Preliminary Population Norms and Effect Sizes. Soc Indic Res. 2006;77: 37-59.
142. Lucas-Carrasco R. The WHO quality of life (WHOQOL) questionnaire: Spanish development and validation studies. Qual Life Res. 2012;21(1): 161-165. doi: 10.1007/s11136-011-9926-3.
143. Tazaki M, Noji A, Nakane Y. WHOQOL. Diagn Ther. 1995;83(12): 2183-2198.
144. Koutra K, Triliva S, Roumeliotaki T, Lionis C, Vgontzas AN. Cross-cultural adaptation and validation of the Greek version of the Family Adaptability and Cohesion Evaluation Scales IV Package (FACES IV Package). J Fam Issues. 2013;34(12): 1647-1672.
145. Lelliott P, Beevor A, Hogman G, Hyslop J, Lathlean J, Ward M. Carers' and users' expectations of services - carer version (CUES-C): A new instrument to support the assessment of carers of people with a severe mental illness. J Ment Health. 2003;2: 143-152. doi: 10.1080/0963823031000103452.
146. Locke HJ, Wallace KM. Short marital-adjustment and prediction tests: Their reliability and validity. Marriage Fam Living. 1959;21: 251-255. doi: 10.2307/348022.
147. Haynes SN, Floyd FJ, Lemsky C, Rogers E, Winemiller D, Heilman N, et al. The Marital Satisfaction Questionnaire for Older Persons. Psychol Assess. 1992;4(4): 473–482. doi: 10.1037/1040-3590.4.4.473.
148. Diener E, Emmons RA, Larsen RJ, Griffin S. The Satisfaction With Life Scale. J Pers Assess. 1985;49(1): 71-75. doi: 10.1207/s15327752jpa4901_13.
149. Mak WWS, Cheung RYM. Affiliate Stigma Among Caregivers of People with Intellectual Disability or Mental Illness. J Appl Res Intellect Disabil. 2008;21(6): 532-545. doi: 10.1111/j.1468-3148.2008.00426.x.
150. Hawthorne G. Measuring Social Isolation in Older Adults: Development and Initial Validation of the Friendship Scale. Soc Indic Res. 2006;77: 521-548. doi: 10.1007/s11205-005-7746-y.
151. Nojima S. Chronicity and family/patient interaction in a Japanese schizophrenic patient population [Internet]. San Francisco (CA): Unpublished Doctor of Nursing Science Dissertation; 1989. Available from: <https://www.proquest.com/openview/5cbba1f731346fe3b710c933b7617c22/1?pq-origsite=gscholar&cbl=18750&diss=y>.
152. Griffiths KM, Christensen H, Jorm AF, Evans K, Groves C. Effect of web-based depression literacy and cognitive-behavioural therapy interventions on stigmatising attitudes to depression: randomised controlled trial. Br J Psychiatry. 2004;185: 342-349. doi: 10.1192/bjp.185.4.342.
153. Reilly MC, Zbrozek AS, Dukes EM. The validity and reproducibility of a work productivity and activity impairment instrument. Pharmacoeconomics. 1993;4(5): 353-365. doi: 10.2165/00019053-199304050-00006.
154. Schene AH, van Wijngaarden B. The involvement evaluation questionnaire. Amsterdam (NL): Department of Psychiatry, University of Amsterdam; 1992.
155. Balducci C, Mnich E, McKee KJ, Lamura G, Beckmann A, Krevers B, et al. Negative impact and positive value in caregiving: validation of the COPE index in a six-country sample of carers. Gerontologist. 2008;48(3): 276-286. doi: 10.1093/geront/48.3.276.
156. McKee KJ, Philip I, Lamura G, Prouskas C, Oberg B, Krevers B, et al. The COPE index--a first stage assessment of negative impact, positive value and quality of support of caregiving in informal carers of older people. Aging Ment Health. 2003;7(1): 39-52. doi: 10.1080/1360786021000006956.
157. Schene AH, van Wijngaarden B, Koeter MW. Family caregiving in schizophrenia: domains and distress. Schizophr Bull. 1998;24(4): 609-618. doi: 10.1093/oxfordjournals.schbul.a033352.
158. van Wijngaarden B, Schene AH, Koeter M, Vázquez-Barquero JL, Knudsen HC, Lasalvia A, et al. Caregiving in schizophrenia: development, internal consistency and reliability of the Involvement Evaluation Questionnaire--European Version. EPSILON Study 4. European Psychiatric Services: Inputs Linked to Outcome Domains and Needs. Br J Psychiatry Suppl. 2000;39: s21-27. doi: 10.1192/bjp.177.39.s21.
159. Corsentino EA, Molinari V, Gum AM, Roscoe LA, Mills WL. Family Caregivers' Future Planning for Younger and Older Adults With Serious Mental Illness (SMI). J Appl Gerontol. 2008;27(4): 466-485. doi: 10.1177/0733464808315290.
160. Platt S, Weyman A, Hirsch S, Hewett S. The Social Behaviour Assessment Schedule (SBAS): Rationale, contents, scoring and reliability of a new interview schedule. Soc Psychiatry. 1980;15: 43-55. doi: 10.1007/BF00577960.
161. Armeli S, Gunthert KC, Cohen LH. Stressor appraisals, coping, and post-event outcomes: The dimensionality and antecedents of stress-related growth. J Soc Clin Psychol. 2001;20(3): 366-395. doi: 10.1521/jscp.20.3.366.22304.
162. Lohrer SP, Lukens EP, Thorning H. Economic expenditures associated with instrumental caregiving roles of adult siblings of persons with severe mental illness. Community Ment Health J. 2007;43(2): 129-151. doi: 10.1007/s10597-005-9026-3.
